# Supplementary material for: C-H-activated Csp2-Csp3 diastereoselective gridization enables ultraviolet-emitting stereo-molecular nanohydrocarbons with mulitple H···H interactions
Source: Nat Commun. 2024 Jun 27;15:5438. doi: 10.1038/s41467-024-48130-6 (PMC11211434; doi:10.1038/s41467-024-48130-6)
Supplement: Supplementary file 1 — Supplementary Information [file 41467_2024_48130_MOESM1_ESM.pdf]

Supplementary Information

C-H-activated Csp^2-Csp^3 diastereoselective gridization enables ultraviolet-emitting stereo-molecular nanohydrocarbons with mulitple H···H interactions

Wei et al.

Supplementary Methods

Supplementary Notes

Section 1. Characteristics of C-H gridization
Section 2. Theoretical calculation datas of TWG-TS1 and TWG-TS2
Section 3. Structural characterization of DWGs and TWGs
Section 4. The crystal data of single crystal of *meso*-DWG1 and *rac*-DWG1 and *cis-trans*-TWG1 and *cis-cis*-TWG1 and co-crystal of *cis-cis*-TWG1 and *cis-trans*-TWG1.
Section 5. The Calculation of strain energies of DWGs, TWGs, SWGs-F and TWGs-Th.
Section 6. Strain and aggregate effects of DWGs and TWGs.
Section 7. Chiroptical properties and UV OLED
Section 8. GC-MS, MALDI-TOF-MS and NMR spectra for all substrates and products.

Supplementary References

Supplementary Methods

Experimental Details and Characterization Data

Typical procedure for the preparation of 2-bromo-9-phenyl-9*H*-fluoren-9-ol from 2-bromo-9*H*-fluoren-9-one

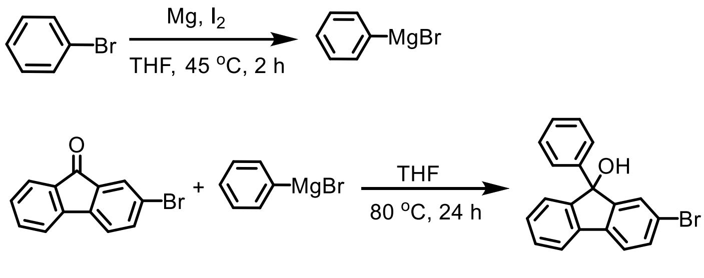

Put bromobenzene (0.236 mg, 1.5 mmol, 1.5 equiv), magnesium (0.036 mg, 1.5 mmol, 1.5 equiv), iodine (one pill) in tetrahydrofuran (2 ml) in a reaction flask. Then the reaction mixture was stirred at 50 ºC for 2 h. After that, dissolve 2-bromo-9*H*-fluoren-9-on (0.259 mg, 1 mmol) in tetrahydrofuran (8 ml) and transfer the reaction mixture to it. After reacting at 80 ºC for 24 h, the reaction was quenched with water and extracted with methylene chloride. The organic layers were then combined, dried (Na_2SO_4) and concentrated under reduced pressure. The residue was purified by flash column chromatography on silica gel to obtain the pure product. The products 2-bromo-9-phenyl-9*H*-fluoren-9-ol were obtained in 89% yield. (White powders, 0.3 g, 0.89 mmol). The NMR spectral data match the previously published data.[1]

2-bromo-9-phenyl-9*H*-fluoren-9-ol (**BrFOH**):

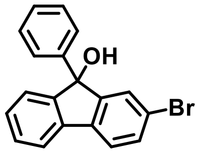

BrFOH

^{1}H NMR (400 MHz, $CDCl_3$) δ 7.66 – 7.64 (d, *J* = 7.6 Hz, 1H), 7.55 – 7.53 (d, *J* = 8.0 Hz, 1H), 7.50 – 7.48 (dd, *J*=8 Hz,2 Hz,1H), 7.45 (s, 1H), 7.41 – 7.35(m, 3H), 7.33 – 7.26 (m, 5H), 2.47 (s, 1H). ^{13}C NMR (100 MHz, $CDCl_3$) δ 150.4, 148.1, 140.4, 136.6, 136.5, 130.2, 127.4, 126.9, 126.4, 126.2, 125.5, 123.3, 122.9, 120.1, 119.5, 118.2, 81.5.

Typical procedure for the preparation of 2-bromo-9-phenyl-9-*H*-fluorene (**1a**) from 2-bromo-9-phenyl-9*H*-fluoren-9-ol

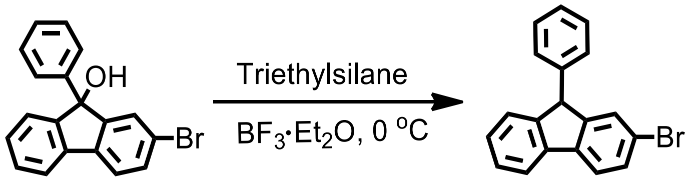

1a

Put 2-bromo-9-phenyl-9*H*-fluoren-9-ol (0.036 g, 1 mmol), triethylsilane (0.128 g, 1.1

mmol, 1.1 equiv), $BF_3 \cdot Et_2O$ (0.426 g, 3 mmol, 3 equiv) in methylene chloride in a reaction flask. Then the reaction mixture was stirred at 0 °C for a while. Upon completion, the reaction was quenched with water and extracted with methylene chloride. The organic layers were then combined, dried (Na_2SO_4) and concentrated under reduced pressure. The residue was purified by flash column chromatography on silica gel to obtain the pure product. The product **1a** was obtained in 95% yield. (White powders, 0.304 g, 0.95 mmol). ^{1}H NMR (400 MHz, $CDCl_3$): δ 7.80 - 7.78 (d, *J* = 7.2 Hz, 1H), 7.68 - 7.66(d, *J* = 8.0 Hz, 1H), 7.54 - 7.52 (d, *J* = 7.6 Hz, 1H), 7.47 (s, 1H), 7.43-7.40 (t, *J* = 6.0 Hz, 1H), 7.32 - 7.30 (m, 5H), 7.11 - 7.09 (d, *J* = 6.8 Hz, 2H), 5.04 (s,1H); ^{13}C NMR (100 MHz, $CDCl_3$): δ 149.9, 147.6, 140.7, 140.0, 130.6, 128.9, 128.6, 128.4, 127.8, 127.6, 127.2, 125.5, 121.3, 121.1, 120.0, 54.4; HRMS(ESI, m/z): calcd for $C_{19}H_{13ws}$Br $[M+H]^+$:321.0273, found:321.0273.

2-Bromo-9-(4-methoxyphenyl)-9H-fluorene (**1b**):

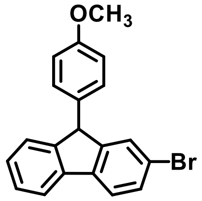

1b

^{1}H NMR (400 MHz, $CDCl_3$): δ 7.78 - 7.76 (d, *J* = 7.8 Hz, 1H), 7.66 - 7.64 (d, *J* = 8.4 Hz, 1H), 7.51 - 7.49 (dd, *J* = 8.4 Hz, 1H), 7.44 (s, 1H), 7.41 - 7.37 (t, *J* = 6.8 Hz, 1H), 7.31 - 7.27 (m, 2H), 7.01 - 6.99 (d, *J* = 8.8 Hz, 2H), 6.84-6.82 (d, *J* = 8.4 Hz, 2H), 4.98 (s, 1H), 3.79 (s, 3H); ^{13}C NMR (100 MHz, $CDCl_3$): δ 158.7, 150.3, 147.9, 139.9, 132.6. 130.4, 129.3, 128.5, 127.7, 125.4, 121.2, 121.0, 119.9, 114.3, 55.3, 53.6. HRMS(ESI, m/z): calcd for $C_{20}H_{15}BrO$ $[M+H]^+$:351.0379, found:351.0374.

2-Bromo-9-(p-tolyl)-9H-fluorene (**1c**):

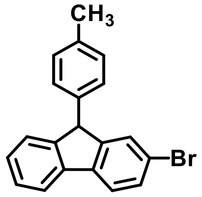

1c

^{1}H NMR (400 MHz, $CDCl_3$): δ 7.79 - 7.77 (d, *J* = 7.6 Hz, 1H), 7.67 - 7.65 (d, *J* = 8 Hz, 1H), 7.52 - 7.50 (dd, *J* = 8 Hz, 1H), 7.46 (s, 1H), 7.42 - 7.38 (t, *J* = 6.8 Hz, 1H), 7.33 - 7.27 (m, 2H), 7.13 - 7.11 (d, *J* = 8 Hz, 2H), 6.99-6.97 (d, *J* = 8 Hz, 2H), 5.01 (s, 1H), 2.35 (s, 3H); ^{13}C NMR (100 MHz, $CDCl_3$): δ 150.1, 147.8, 140.0, 137.6. 136.8, 130.5,

129.6, 128.6, 128.2, 127.8, 127.5, 127.3, 125.4, 121.2, 121.1, 119.9, 54.0, 21.2. HRMS(ESI, m/z): calcd for $C_{20}H_{15}Br$ $[M+H]^+$:335.0430, found:334.0420.

2-Bromo-9-(4-ethylphenyl)-9H-fluorene (**1d**):

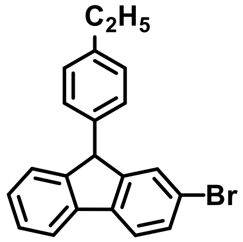

1d

^{1}H NMR (400 MHz, $CDCl_3$): δ 7.77 - 7.76 (d, *J* = 7.6 Hz, 1H), 7.65 - 7.63 (d, *J* = 8 Hz, 1H), 7.51 - 7.49 (d, *J* = 8 Hz, 1H), 7.46 (s, 1H), 7.40 - 7.37 (t, *J* = 8.4 Hz, 1H), 7.32 - 7.24 (m, 2H), 7.14 - 7.12 (d, *J* = 8 Hz, 2H), 7.00-6.98 (d, *J* = 8 Hz, 2H), 5.01 (s, 1H), 2.67-2.61 (m, 2H), 1.26-1.22 (m, 3H); ^{13}C NMR (100 MHz, $CDCl_3$): δ 148.2, 145.9, 141.2, 138.1, 135.9, 126.7, 126.3, 125.8, 125.6, 123.5, 119.3, 119.2, 118.0, 52.2, 26.6, 13.6. HRMS(ESI, m/z): calcd for $C_{21}H_{17}Br$ $[M+H]^+$:349.0586, found:349.0589.

2-Bromo-9-(4-hexylphenyl)-9H-fluorene (**1e**):

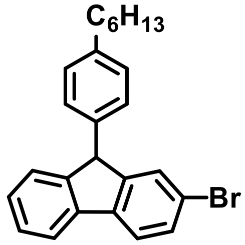

1e

^{1}H NMR (400 MHz, $CDCl_3$): δ 7.72- 7.70 (d, *J* = 7.6 Hz, 1H), 7.60 - 7.58 (d, *J* = 8 Hz, 1H), 7.46 - 7.44 (d, *J* = 9.2 Hz, 1H), 7.41 (s, 1H), 7.35 - 7.32 (t, *J* = 7.2 Hz, 1H), 7.23 - 7.19 (m, 2H), 7.07 - 7.05 (d, *J* = 8 Hz, 2H), 6.94-6.92 (d, *J* = 8 Hz, 2H), 4.96 (s, 1H), 2.56-2.52 (t, *J* = 7.6 Hz,2H), 1.60-1.52 (m, 2H), 1.28 (s, 6H),0.86 (s, 3H); ^{13}C NMR (100 MHz, $CDCl_3$): δ 148.2, 145.9, 140.0, 138.1, 135.8, 128.6, 127.0, 126.7, 126.3, 125.8, 125.6, 123.6, 119.3, 119.2, 118.0, 52.2, 33.8, 29.9, 29.5, 27.3, 20.8, 12.3. HRMS(ESI, m/z): calcd for $C_{25}H_{25}Br$ $[M+H]^+$:405.1212, found:405.1208.

2-Bromo-9-(4-(octyloxy)phenyl)-9H-fluorene (**1f**):

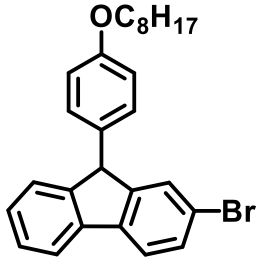

1f

^{1}H NMR (400 MHz, $CDCl_3$): δ 7.75- 7.73 (d, *J* = 7.2 Hz, 1H), 7.63 - 7.61 (d, *J* = 8 Hz, 1H), 7.48 - 7.46 (d, *J* = 8 Hz, 1H), 7.42 (s, 1H), 7.38 - 7.36 (t, *J* = 7.2 Hz, 1H), 7.29 - 7.23 (m, 2H), 6.97 – 6.95 (d, *J* = 8.4 Hz, 2H), 6.81-6.79 (d, *J* = 8.4 Hz, 2H), 4.95 (s, 1H), 3.92-3.89 (t, *J* = 12.8 Hz,2H), 1.79-1.72 (m, 2H), 1.52-1.28 (m, 10H),0.88 (s, 3H); ^{13}C NMR (100 MHz, $CDCl_3$): δ 158.4, 150.3, 148.0, 139.9, 132.3, 130.4, 129.3, 128.6, 127.7, 127.5, 125.4, 121.2, 121.1, 119.9, 114.8, 68.0, 53.7, 31.9, 29.4, 29.3, 26.1, 22.7, 14.2. HRMS(ESI, m/z): calcd for $C_{27}H_{29}BrO$ $[M+H]^+$:449.1475, found:449.1463.

2-(2-Bromo-9H-fluoren-9-yl)thiophene (**1g**):

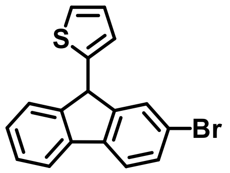

1g

^{1}H NMR (400 MHz, $CDCl_3$): δ 7.44- 7.42 (d, *J* = 7.6 Hz, 1H), 7.32 - 7.30 (d, *J* = 8 Hz, 1H), 7.27 (s, 1H), 7.21 - 7.18 (d, *J* = 9.6 Hz, 1H), 7.15 - 7.13 (d, *J* = 9.6 Hz, 1H), 7.10 – 7.07 (m, 1H), 7.02-6.98 (m, 1H), 6.83-6.85 (m, 1H), 6.66-6.65 (m,2H), 5.00 (s, 1H), ^{13}C NMR (100 MHz, $CDCl_3$): δ 146.4, 144.2, 140.6, 137.2, 128.5, 126.2, 125.6, 125.5, 124.6, 123.2, 123.0, 122.2, 119.0, 118.8, 117.7, 46.6. HRMS(ESI, m/z): calcd for $C_{17}H_{11}BrS$ $[M+H]^+$:326.9838, found:326.9840.

2-(3-Bromophenyl)-9-phenyl-9H-fluorene (**1h**)

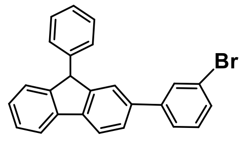

1h

^{1}H NMR (400 MHz, $CDCl_3$): δ 7.85 - 7.80 (m, 2H), 7.70 (s, 1H), 7.58 - 7.56 (dd, J = 8 Hz, 1H), 7.53 - 7.51 (m, 2H), 7.48 - 7.43 (m, 2H), 738 - 7.26 (m, 6H), 7.18 - 7.16 (d, J = 8.4 Hz, 2H), 5.14 (s, 1H); ^{13}C NMR (100 MHz, $CDCl_3$): δ 148.7, 148.3, 143.4, 141.3, 140.9, 138.9, 130.2, 130.1, 128.8, 128.4, 127.6, 127.5, 127.0, 126.6, 125.8, 122.9, 54.5.HRMS(ESI, m/z): calcd for $C_{25}H_{17}Br$ $[M+H]^+$:397.0589, found:397.0591.

2-(3-bromophenyl)-9-(4-(octyloxy)phenyl)-9H-fluorene (**1i**)

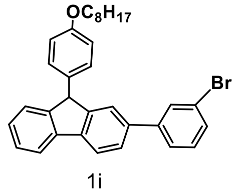

1i

^{1}H NMR (400 MHz, $CDCl_3$): δ 7.88 - 7.84 (t, J = 7.8 Hz, 2H), 7.78 (s, 1H), 7.62 - 7.60 (d, J = 8 Hz, 1H), 7.54 - 7.42 (m, 4H), 7.39 - 7.28 (m, 3H), 7.09 - 7.07 (d, J = 8 Hz, 2H), 6.88 - 6.86 (d, J = 8 Hz, 2H), 5.08 (s, 1H), 3.97 - 3.94 (t, J = 6.6 Hz, 2H), 1.84 - 1.77 (m, 2H), 1.51 - 1.47 (t, J = 7.4 Hz, 2H), 1.41 - 1.34 (m, 8H), 0.97 - 0.94 (t, J = 6.6 Hz, 3H); ^{13}C NMR (100 MHz, $CDCl_3$): δ 158.3, 149.1, 148.7, 143.5, 140.9, 140.4, 138.8, 133.2, 133.0, 130.3, 130.1, 129.4, 127.6, 127.4, 126.5, 125.8, 125.4, 124.0, 122.9, 120.3, 120.1, 114.8, 68.0, 53.9, 31.9, 29.5, 29.4, 26.2, 22.8, 14.3. HRMS(ESI, m/z): calcd for $C_{33}H_{33}BrO$ $[M+H]^+$:525.1788, found:525.1784.

Typical procedure for the preparation of windmill gridarene of phenyl-fluorene from 2-bromo-9-phenyl-9*H*-fluorene

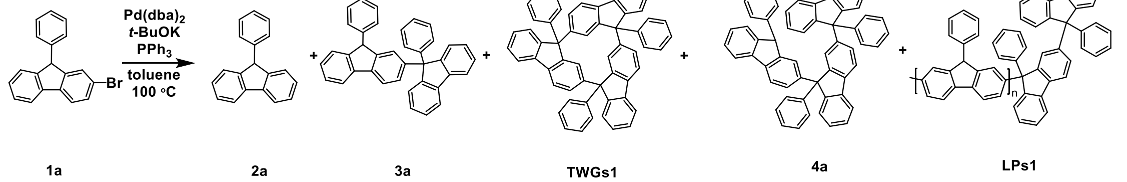

1a 2a 3a TWGs1 4a LPs1

To a solution of 2-bromo-9-phenyl-9*H*-fluorene (**1a**) (0.32 g, 1 mmol), $Pd(dba)_2$ (0.040 g, 0.07 mmol, 0.07 equiv), PPh_3 (0.037 g, 0.14 mmol, 0.14 equiv) in dry toluene (25 ml)

in a Schlenk tube, KO^{t}Bu (0.135 g, 1.2 mmol, 1.2 equiv) was added under argon atmosphere in a glove box. Then the reaction mixture was stirred at 100 °C for 56 h. Upon completion, the reaction was quenched with water and extracted with methylene chloride. The organic layers were then combined, dried (Na_2SO_4) and concentrated under reduced pressure. The residue was purified by flash column chromatography on silica gel to obtain the pure product. The products **TWGs** were obtained in 32% yield (White powders, 0.077 g, 0.107 mmol). The product **2a** was obtained in 17% yield (White powders, 0.041 g, 0.17 mmol). The product **3a** was obtained in 10% yield (White powders, 0.024 g, 0.05 mmol). The product **4a** was obtained in 7% yield (White powders, 0.017 g, 0.023 mmol). The product **LPs1** was obtained in 33% yield.

***cis-trans*-TWG1**

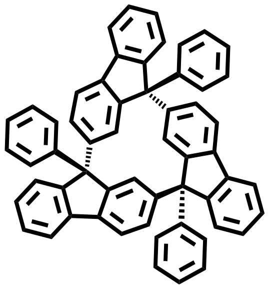

1H NMR (400 MHz, CDCl3): δ 7.76 – 7.72 (d, J = 7.6 Hz, 2H), 7.67 – 7.63 (d, J = 7.4 Hz, 1H), 7.57 – 7.52 (m, 4H), 7.49 – 7.45 (m, 3H), 7.43 – 7.34 (m, 6H), 7.33 – 7.26 (m, 9H), 7.23 – 7.16 (m, 5H), 7.04 – 6.94 (m, 3H), 6.69 – 6.64 (m, 4H), 6.02 – 6.00 (d, J = 1.6 Hz, 1H); 13C NMR (101 MHz, CDCl3) δ 157.41, 152.81, 151.50, 150.70, 149.93, 149.87, 147.13, 146.43, 146.29, 145.21, 144.22, 141.56, 140.54, 140.36, 140.31, 139.43, 138.12, 137.59, 130.62, 130.23, 129.64, 128.89, 128.83, 128.51, 128.23, 128.12, 127.94, 127.87, 127.76, 127.71, 127.70, 127.66, 127.64, 127.34, 127.30, 126.99, 126.87, 126.78, 126.41, 126.38, 126.22, 123.79, 120.67, 120.51, 120.37, 119.76, 119.37, 119.29, 67.38, 65.36, 65.30. HRMS(ESI, m/z): calcd for $C_{57}H_{36}$ $[M+Na]^+$: 743.2709, found: 743.2722.

***cis-cis*-TWG1**

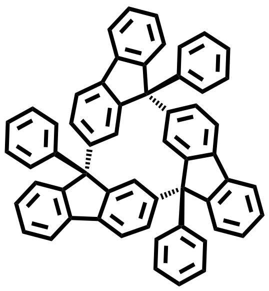

^{1}H NMR (400 MHz, $CDCl_3$): δ 8.14 (s, 3H), 7.70 – 7.61 (d, *J* = 7.0 Hz, 3H), 7.44 (s, 6H), 7.31 (s, 18H), 7.23 – 7.14 (d, *J* = 7.7 Hz, 3H), 6.74 – 6.55 (d, *J* = 8.3 Hz, 3H); ^{13}C NMR (101 MHz, $CDCl_3$): δ 153.93, 149.38, 146.11, 144.26, 139.59, 138.22, 129.93, 128.72, 128.48, 127.66, 127.23, 126.98, 126.72, 124.48, 120.56, 118.06, 66.77. HRMS(ESI, m/z): calcd for $C_{57}H_{36}$ $[M+H]^+$: 721.2890; Found: 721.2885.

9-Phenyl-fluorene (**2a**):

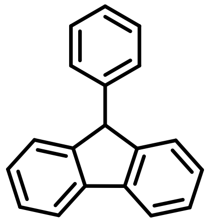

2a

^{1}H NMR (400 MHz, $CDCl_3$): δ 7.80 - 7.78 (d, J = 7.6 Hz, 2H), 7.39 - 7.35 (t, J = 7.2 Hz, 2H), 7.32- 7.30 (d, J = 7.2 Hz, 2H), 7.27 - 7.26 (m, 2H), 7.24 - 7.21 (m, 3H), 7.09 - 7.07 (d, J = 8.0 Hz, 2H), 5.00 (s, 1H); ^{13}C NMR (100 MHz, $CDCl_3$): δ 147.9, 141.6, 141.0, 128.7, 128.4, 127.4, 126.9, 125.4, 119.9, 54.5; [2]

9-9'-Diphenyl-9*H*,9'*H*-2,9'-bifluorene (**3a**):

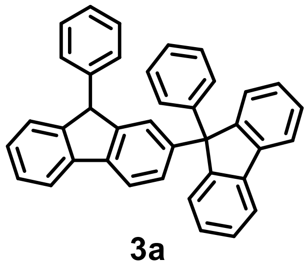

3a

^{1}H NMR (400 MHz, $CDCl_3$): δ 7.81 - 7.79 (d, J = 7.6 Hz, 2H), 7.76 - 7.74 (d, J = 7.6 Hz, 1H), 7.64 - 7.62 (d, J = 8.0 Hz, 1H), 7.44 - 7.42 (d, J = 7.6 Hz, 1H), 7.40 - 7.33 (m, 6H), 7.30 - 7.25 (m, 6H), 7.20 - 7.16 (m, 6H), 7.09 - 7.07 (dd, J = 8.0 Hz, 2.0 Hz, 2H), 5.05 (s, 1H); ^{13}C NMR (100 MHz, $CDCl_3$): δ 151.3 (d, J = 7.9 Hz), 148.0, 147.7, 146.3, 145.2, 141.6, 140.5, 140.2 (d, J = 5.3 Hz), 140.0, 128.6, 128.3, 128.1, 127.8, 127.5, 127.3, 127.2, 127.1, 126.8, 126.7,126.2, 125.9, 125.2, 120.3, 119.9, 119.5, 65.8, 54.3; HRMS(ESI, m/z): calcd for $C_{38}H_{26}$ $[M+H]^+$:483.2107, found:483.2098.

9,9',9''-Triphenyl-9*H*,9'*H*,9''*H*-2,9':2',9''-terfluorene (**Trimerized product, 4a)**:

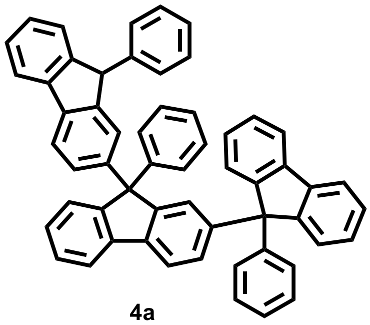

4a

^{1}H NMR (400 MHz, $CDCl_3$): δ 7.78 (3H), 7.70- 7.65 (m, 1H), 7.63- 7.57 (m, 1H), 7.53 - 7.52 (d, J = 6.4Hz, 2H), 7.36- 7.24 (m, 13H), 7.21 - 7.16 (m, 8H), 7.12 - 7.11 (4H), 7.06 - 7.05 (d, J = 6.8 Hz, 3H), 7.02 - 6.96 (m, 2H); 4.98 (s, 1H); ^{13}C NMR (100 MHz, $CDCl_3$): δ 151.3, 151.2, 151.1, 148.1, 147.9, 147.7, 147.4, 146.1, 145.9, 145.5, 145.3, 145.2, 141.6, 141.2, 140.4, 140.2, 140.1, 139.9, 139.8, 138.7, 138.6, 128.7,128.6, 128.2,128.1, 128.0, 127.8, 127.7, 127.5, 127.4, 127.3, 127.2,126.9, 126.8, 126.7, 126.6,

126.5, 126.2,126.1, 125.7, 125.3, 125.2, 125.1, 120.2, 119.8 (d, *J* = 3.1 Hz), 119.5, 119.4,65.8, 65.7, 54.3; HRMS(ESI, m/z): calcd for $C_{57}H_{38}$ $[M+H]^+$:723.3046, found:723.3055.

TWG2

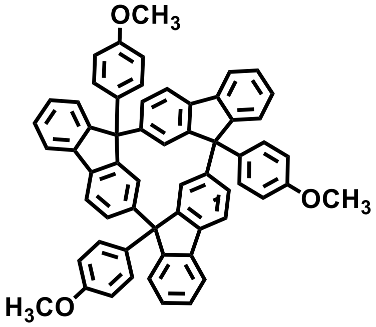

^{1}H NMR (400 MHz, $CDCl_3$): δ7.78 - 7.75 (m, 2H), 7.68 - 7.66 (d, *J* = 7.6 Hz, 1H), 7.60 - 7.56 (m, 4H), 7.50 - 7.48 (d, *J* = 7.6 Hz, 1H), 7.46 - 7.40 (m, 4H), 7.38 - 7.30 (m, 6H), 7.28 – 7.22 (m, 4H), 6.88 – 6.85 (d, *J* = 8.8 Hz, 2H), 6.75 – 6.73 (d, *J* = 8.8 Hz, 2H), 6.96 – 6.63 (m, 4H), 6.55 – 6.53 (d, *J* = 8.8 Hz, 2H), 6.01 (s, 1H), 3.83 (s, 3H), 3.75 (s, 3H), 3.65 (s, 3H); ^{13}C NMR (100 MHz, $CDCl_3$): δ158.5, 158.1, 157.7, 153.3, 152.7, 151.0, 150.2, 147.3, 146.6, 140.5, 140.3, 140.2, 139.3, 138.0, 137.4, 137.2, 136.2, 130.5, 130.1, 130.0, 129.5, 129.3, 128.8, 128.7, 127.7, 127.6, 127.3, 126.9, 126.3, 126.2, 120.6, 120.5, 120.4, 119.7, 119.3, 113.9, 113.5, 113.3, 31.6, 22.7, 14.6. HRMS(ESI, m/z): calcd for $C_{60}H_{42}O_3$ $[M+H]^+$:811.3207, found:811.3192.

TWG3

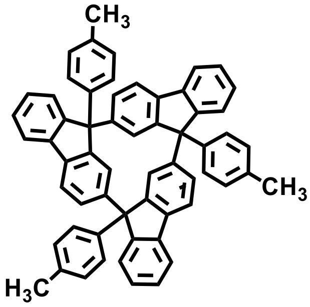

^{1}H NMR (400 MHz, $CDCl_3$): δ 8.17 (s, 3H), 7.74 - 7.72 (d, *J* = 7.6 Hz, 2H), 7.65 - 7.63 (d, *J* = 8.4 Hz, 1H), 7.60 - 7.58 (d, *J* = 6.8 Hz, 1H), 7.56 - 7.52 (m, 3H), 7.46 - 7.44 (d, *J* = 7.2 Hz, 1H), 7.39 - 7.37 (d, *J* = 8.0 Hz, 3H), 7.35 - 7.33 (d, *J* = 7.6 Hz, 3H), 7.31 - 7.28 (m, 5H), 7.23 - 7.18 (m, 4H), 7.11 - 7.09 (d, *J* = 8.0 Hz, 3H), 7.00 - 6.98 (d, *J* = 8.0 Hz, 2H), 6.79 - 6.77 (d, *J* = 7.6 Hz, 2H), 6.67 - 6.63 (m, 1H), 6.58 - 6.56 (d, *J* = 8.0 Hz, 1H), 6.00 (s, 1H), 2.28 – 2.35 (m, 9H);^{13}C NMR (100 MHz, $CDCl_3$): δ157.6, 154.1, 153.1, 151.7, 150.9, 150.2, 150.1, 147.0, 146.6, 146.3, 142.2, 141.2, 140.5, 140.4,140.2,139.6, 139.4, 138.5, 138.1, 137.5, 136.5, 136.3, 136.0,130.6, 130.2,129.9, 129.6, 129.4, 129.2, 128.8, 128.7, 128.4, 128.1, 127.8, 127.7, 127.6, 127.5, 127.2, 127.0, 126.6, 126.3, 126.2, 123.7, 120.6, 120.5, 120.3, 120.1, 119.7, 119.2, 117.9, 29.7, 21.0. HRMS(ESI, m/z): calcd for $C_{60}H_{42}O_3$ $[M+H]^+$:811.3207, found:811.3192.

TWG4

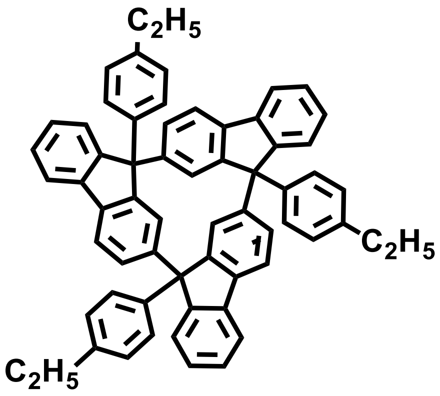

^{1}H NMR (400 MHz, $CDCl_3$): δ 8.21 (s, 3H), 7.73 - 7.71 (d, J = 7.2 Hz, 2H), 7.64 - 7.62 (d, J = 6.8 Hz, 1H), 7.58 - 7.52 (m, 4H), 7.46 - 7.44 (m, J = 8.0 Hz, 1H), 7.40 - 7.34 (m, 6H), 7.31 - 7.28 (m, 4H), 7.21 - 7.19 (m, 4H), 7.11 - 7.09 (d, J = 7.2 Hz, 2H), 7.00 – 6.988 (d, J = 7.6 Hz, 2H), 6.80 – 6.78 (d, J = 7.6 Hz, 2H), 6.67 (s, 1H), 6.00 (s, 1H), 3.65 - 3.59 (m, 8H), 2.66 -2.54 (m, 5H), 2.47 -2.41 (m, 2H); ^{13}C NMR (100 MHz, $CDCl_3$): δ157.5, 153.1, 151.7, 150.9, 150.2, 147.2, 146.6, 146.4, 142.8, 142.5, 142.4, 142.2, 141.3, 140.5, 10.4, 140.3, 139.4, 138.7, 138.1, 137.5, 130.7, 130.2, 129.6, 128.9, 128.8, 128.1, 127.9, 127.7, 127.6, 127.5, 127.4, 127.2, 127.0, 126.4, 126.3, 123.7, 120.6, 120.5, 120.3, 119.7, 119.2, 119.2, 28.4, 28.1, 15.5, 15.3, 15.2. HRMS(ESI, m/z): calcd for $C_{63}H_{48}$ $[M+H]^+$:805.3829, found:805.3831.

TWG5

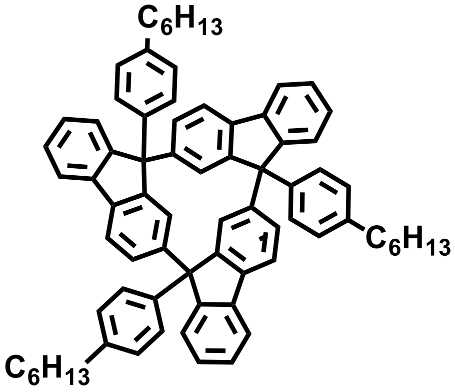

^{1}H NMR (400 MHz, $CDCl_3$): δ 7.74 - 7.72 (m, 2H), 7.65 - 7.63 (d, J = 7.2 Hz, 1H), 7.54 (s, 4H), 7.47 - 7.45 (d, J = 7.2 Hz, 1H), 7.41 - 7.34 (m, 6H), 7.30 - 7.28 (m, 4H), 7.22 - 7.18 (m, 4H), 7.08 - 7.06 (d, J = 8.0 Hz, 2H), 6.98 - 6.96 (d, J = 7.6 Hz, 2H), 6.78 - 6.76 (d, J = 8.0 Hz, 2H), 6.68 (s, 1H), 6.64 - 6.56 (m, 2H), 6.00 (s, 1H), 2.60 -2.50 (m, 4H), 2.40 -2.37 (m, 2H), 1.62 -1.55 (m, 7H), 1.32 - (m, 26H); ^{13}C NMR (100 MHz, $CDCl_3$): δ156.5, 149.1, 145.5, 140.3, 139.9, 139.3, 129.7, 129.2, 128.6, 127.8, 127.4, 127.0, 126.9, 126.8, 126.6, 126.5, 126.4, 126.2, 126.1, 126.0, 125.4, 125.3, 122.6, 119.5, 119.4, 119.3, 118.6, 118.2, 118.1, 66.1, 64.1, 64.0 ,34.5, 34.3, 30.7, 30.6, 30.3, 30.2, 30.1, 28.7, 28.1, 28.0, 21.6, 21.5, 13.1, 13.0. HRMS(ESI, m/z): calcd for $C_{75}H_{72}$ $[M+H]^+$:973.5707, found:973.5707.

TWG7

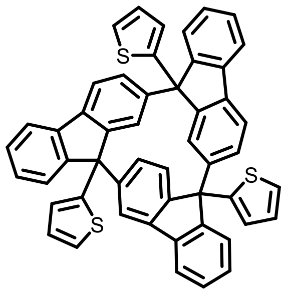

^{1}H NMR (400 MHz, $CDCl_3$): δ 8.51 (s, 1H) 7.74 -7.71 (m, 2H), 7.65 – 7.59 (m, 4H), 7.54 -7.52 (d, J = 8.0 Hz, 1H), 7.48 (m,1H), 7.42 - 7.39 (m, 3H), 7.35 - 7.30 (m, 4H), 7.25 - 7.23 (m, 3H), 7.15 -7.11 (d, J = 9.2 Hz, 2H), 7.09 - 7.06 (m, 1H), 7.00 - 6.98 (m, 1H), 6.93 -6.91 (d, J = 8.0 Hz, 1H), 6.87-6.85 (d, J = 7.2 Hz 1H), 6.67 (s, 1H), 6.60-6.56 (m, 2H), 6.45 (s, 1H), 6.00 (s, 1H); ^{13}C NMR (100 MHz, $CDCl_3$): δ156.1, 151.3, 150.3, 149.0, 147.8, 146.5, 145.9, 145.2, 144.8, 144.1, 138.9, 138.8, 138.7, 138.5, 136.1, 129.3, 128.7, 128.5, 127.6, 127.3, 127.1, 127.0, 126.8, 126.6, 126.5, 126.4, 126.3, 126.1, 125.7, 125.4, 125.2, 125.1, 124.7, 124.1, 124.0, 123.9, 123.3, 123.1, 122.2, 119.7, 119.6, 119.4, 118.7, 118.5. HRMS(ESI, m/z): calcd for $C_{51}H_{30}S_3$ $[M+H]^+$:739.1582, found:739.1589.

3h

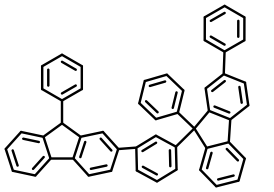

^{1}H NMR (400 MHz, CDCl3): δ 7.91 - 7.75 (m, 4H), 7.71 - 7.53 (m, 5H), 7.52 - 7.26 (m, 15H), 7.26 - 7.04 (m, 9H), 5.05 (s, 1H); 13C NMR (101 MHz, CDCl3): δ 151.84, 151.80, 151.44, 151.42, 148.64, 148.47, 148.45, 148.25, 148.07, 146.44, 145.85, 145.83, 141.94, 141.86, 141.85, 141.44, 141.39, 141.37, 141.32, 141.23, 141.20, 140.83, 140.80, 140.73, 140.65, 140.48, 140.42, 140.38, 140.15, 140.13, 139.86, 139.49, 129.18, 128.78, 128.73, 128.64, 128.43, 128.32, 128.24, 127.80, 127.77, 127.64, 127.44, 127.40, 127.35, 127.32, 127.23, 127.21, 127.20, 127.00, 126.93, 126.83, 126.79, 126.73, 126.55, 126.32, 126.26, 125.61, 125.59, 125.38, 124.97, 124.94, 124.26, 124.14, 124.09, 120.52, 120.49, 120.30, 120.27, 120.23, 120.07, 120.01, 119.94, 65.69, 54.57, 54.44. HRMS(ESI, m/z): calcd for $C_{50}H_{34}$ $[M+H]^+$:635.2733, found:635.2745.

***meso*-DWG1**

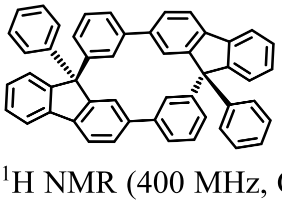

^{1}H NMR (400 MHz, CDCl3): δ 7.80 - 7.77 (d, J = 7.5 Hz, 2H), 7.77 -7.73 (d, J = 7.7

Hz, 2H), 7.67 (s, 2H), 7.64 - 7.60 (d, J = 7.7 Hz, 2H), 7.54 - 7.50 (dd, J = 7.8, 1.5 Hz, 2H), 7.47 - 7.43 (d, J = 7.4 Hz, 2H), 7.43 - 7.38 (td, J = 7.4, 1.1 Hz, 2H), 7.37 - 7.31 (m, 6H), 7.27 (s, 1H), 7.25 - 7.23 (d, J = 7.8 Hz, 1H), 7.16 - 7.10 (dd, J = 5.1, 1.7 Hz, 6H), 7.06 - 7.00 (dd, J = 6.9, 2.9 Hz, 4H); 13C NMR (101 MHz, CDCl3) δ 151.04, 149.92, 145.68, 143.37, 142.30, 141.84, 140.27, 139.12, 134.13, 129.46, 128.29, 128.26, 127.95, 127.75, 127.51, 126.72, 126.57, 126.16, 125.80, 123.50, 120.45, 119.92, 65.17. HRMS(ESI, m/z): calcd for $C_{50}H_{32}$ $[M+H]^+$: 633.2577, found: 633.2574.

***rac*-DWG1**

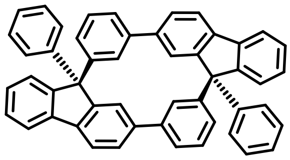

^{1}H NMR (400 MHz, $CDCl_3$): δ 7.85 - 7.78 (dd, *J* = 12.4, 5.3 Hz, 6H), 7.77 - 7.74 (d, *J* = 7.0 Hz, 2H), 7.72 - 7.67 (d, *J* = 7.9 Hz, 4H), 7.53 - 7.45 (m, 4H), 7.44 - 7.30 (m, 6H), 7.23 - 7.14 (d, *J* = 7.1 Hz, 6H), 7.08 - 6.99 (d, *J* = 8.1 Hz, 4H); ^{13}C NMR (101 MHz, $CDCl_3$): δ 150.36, 149.53, 146.03, 142.01, 141.70, 140.25, 138.59, 133.23, 130.48, 128.50, 128.16, 127.93, 127.47, 127.43, 127.41, 126.63, 126.17, 125.69, 123.31, 119.82, 119.51, 64.52. HRMS(ESI, m/z): calcd for $C_{50}H_{32}$ $[M+H]^+$: 633.2577, found: 633.2578.

***cis-trans*-TWG8**

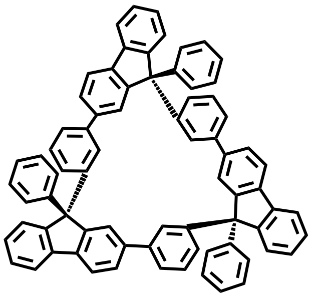

^{1}H NMR (400 MHz, $CDCl_3$): δ 7.81 - 7.74 (m, 5H),7.70 -7,69 (d, *J* = 7.6, 1H), 7.62 - 7.60 (d, *J* = 6.0, 2H), 7.47 - 7.41 (m, 2H), 7.38 - 7.33 (m, 9H),7.31 -7.27 (m, 8H), 7.23 - 7.20 (m, 8H),7.18 -7.14 (m, 9H), 7.02 - 7.00 (d, *J* = 8.8, 1H),6.89 - 6.86 (d, *J* = 9.6,1H), 6.65 - 6.63 (d, *J* = 8.8, 1H); ^{13}C NMR (100 MHz, $CDCl_3$): δ155.3, 152.2, 151.8, 151.4, 147.1, 146.9, 146.2, 145.2, 144.1, 142.2, 141.8, 141.6, 141.4, 140.0, 139.9, 139.3, 133.4, 128.4, 128.3, 128.1, 127.8, 127.0, 126.8, 126.5, 124.8, 120.3. HRMS(ESI, m/z): calcd for $C_{57}H_{36}$ $[M+H]^+$: 949.3829, found: 949.3843.

***cis-cis*-TWG8**

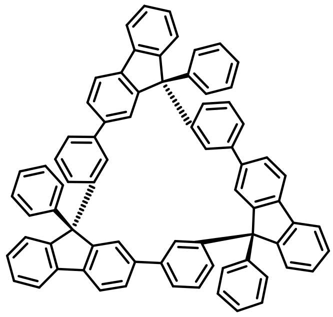

^{1}H NMR (400 MHz, $CDCl_3$): δ 7.75 - 7.73 (d, J = 7.6, 3H), 7.71 -7,69 (d, J = 8, 2H), 7.46 -7.44 (d, J = 7.2, 3H), 7.39 - 7.37 (d, J = 6.4, 3H), 7.36 - 7.34 (t, J = 7.6, 6H),7.31 -7.30 (t, J = 6.8, 10H), 7.27 - 7.25 (d, J = 8.4, 4H),7.22 -7.20 (d, J = 6.8, 5H), 7.12 (s, 3H), 7.09 - 7.05 (m, 4H), 7.02 (s, 2H), 6.92 - 6.90 (d, J = 8.0, 3H); ^{13}C NMR (100 MHz, $CDCl_3$): δ151.6, 146.4, 145.0, 142.5, 141.8, 139.9, 139.1, 128.3, 128.1, 127.7, 127.5, 127.4, 126.7, 126.1, 126.0, 125.8, 125.4, 120.2, 120.1. HRMS(ESI, m/z): calcd for $C_{57}H_{36}$ [M+Na] $^{+}$: 971.3648, found: 971.3647.

TWG9

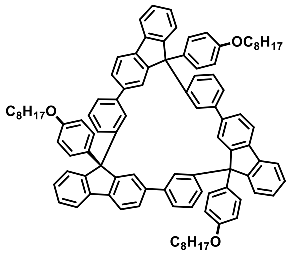

^{1}H NMR (400 MHz, $CDCl_3$): δ 7.82 - 7.80 (d, J = 8 Hz, 2H), 7.77 - 7.65 (m, 16H), 7.45 - 7.28 (m, 29H), 7.25 - 7.03 (m, 39H), 6.97 (S, 1H), 6.92 (s, 1H), 6.85- 6.80 (m, 8H), 6.70- 6.65 (m, 8H), 6.64 - 6.62 (d, J = 8 Hz, 2H), 3.99 - 3.79 (m, 15H), 1.83-1.76 (m, 15H), 1.52-1.43 (m, 15H), 1.38-1.26 (m, 61H), 0.91-0.83 (m, 22H); ^{13}C NMR (100 MHz, $CDCl_3$): δ 158.2, 158.0, 157.9, 157.7, 152.8, 152.6, 152.1, 151.9, 151.8, 147.4, 147.2, 146.4, 142.2, 141.8, 141.7, 141.6, 141.4, 139.9, 139.8, 139.8, 139.8, 139.6, 139.2, 139.2, 139.0, 138.9, 136.7, 135.9, 135.4, 129.3, 129.2, 128.5, 128.4, 128.1, 128.1, 127.8, 127.7, 127.6, 127.4, 127.3, 127.1, 126.8, 126.7, 126.4, 126.0, 125.9, 125.5, 125.4, 125.2, 124.7, 124.6, 120.2, 120.1, 120.0, 114.2, 114.1, 114.1, 68.0, 67.9, 65.0, 64.9, 64.4, 31.9, 31.9, 31.8, 31.8, 29.7, 29.5, 29.5, 29.5, 29.4, 29.4, 29.4, 29.3, 29.3, 26.2, 26.2, 26.1, 22.7, 22.7, 22.7, 14.1. HRMS(ESI, m/z): calcd for $C_{99}H_{96}O_3$ [M+Na] $^{+}$: 1355.7252, found: 1355.7253.

Procedure for the preparation of 2-bromo-9-octyl-9*H*-fluorene from 2-bromo-9*H*-fluorene.

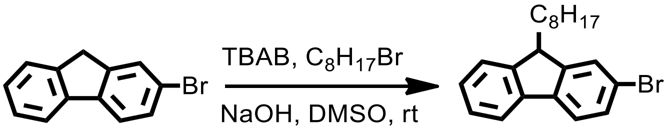

In a dried reaction flask (150 mL), add 2-Bromo-9*H*-fluorene (0.244 g, 1 mmol) and

tetrabutylammonium bromide (TBAB) (0.005 g, 0.016 mmol), evacuate and fill with nitrogen three times. Then inject 1-bromooctane (0.173 g, 0.9 mmol, 0.9 equiv) and DMSO (20 ml) into the reaction flask with a syringe and stir well, finally add sodium hydroxide solution (0.036 g, 0.9 mmol, 0.9 equiv) slowly. The mixture was stirred at room temperature for 5 hours. Upon completion, the reaction was quenched with water and extracted with methylene chloride. The organic layers were then combined, dried (Na_2SO_4) and concentrated under reduced pressure.[3] The residue was purified by flash column chromatography on silica gel to obtain the pure product. The product 2-bromo-9-octyl-9-H-fluorene was obtained in 50% yield. (White liquid, 0.178 g, 0.5 mmol). ^{1}H NMR (400 MHz, $CDCl_3$): δ 7.78-7.71 (d, J = 6.8 Hz, 1H), 7.69-7.65 (s, 1H), 7.65-7.60 (d, J = 8.0 Hz, 1H), 7.56-7.48 (t, J = 8.0 Hz, 2H), 7.43-7.32 (m, 2H), 4.02-3.95 (t, J = 5.6 Hz, 1H), 2.09-1.92 (m, 2H), 1.36-1.13 (m, 12H), 0.96-0.84 (t, J = 6.8 Hz, 3H); ^{13}C NMR (100 MHz, $CDCl_3$): δ 149.8, 147.3, 140.2, 130.0, 127.6, 127.2, 127.1, 124.4, 121.1, 119.9, 47.6, 32.9, 31.9, 29.9,29.3, 25.6, 22.7, 14.1; MALDI-TOF MS: m/z calcd for [M] $C_{21}H_{25}Br$: 356.11; found, 356.00.

Supplementary Notes

Section 1. Characteristics of C-H gridization

Supplementary Table 1 | Optimization studies for Pd-PPh_3-controlled reaction of 2-bromo-9-aryl-9*H*-fluorenes[a].

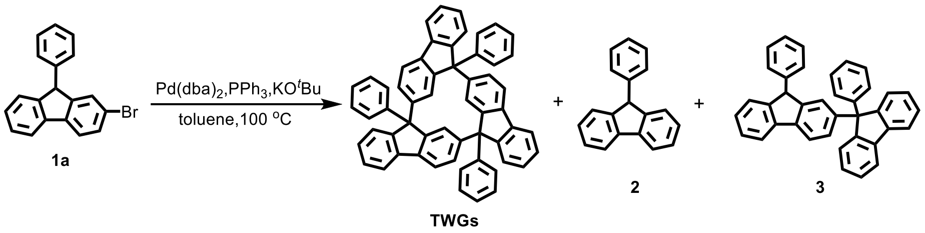

Entry	1	Ligand	Base	Conc. (mM)	Yield[b] (%)	*Dr*[e] *cis-trans/cis-cis*
1	**1a**	PPh_3	KO^{t}Bu	0.4	51[c] 49[d]	-
2	**1a**	PPh_3	KO^{t}Bu	10	10	-
3	**1a**	PPh_3	KO^{t}Bu	20	22	-
4	**1a**	PPh_3	KO^{t}Bu	60	20	-
5	**1a**	PPh_3	KO^{t}Bu	80	0	-
6	**1a**	PPh_3	KO^{t}Bu	100	0	-
7	**1a**	PPh_3	KO^{t}Bu	30	32	77:23
8	**1a**	PPh_3	KO^{t}Bu	40	32	82:18

9	**1a**	PPh_3	KO^{t}Bu	50	30	74:26
10	**1a**	Dppf	KO^{t}Bu	40	0	-
11	**1a**	PCy_3	KO^{t}Bu	40	0	-
12	**1a**	P^{t}Bu$_3$	KO^{t}Bu	40	0	-
13	**1a**	PPh_3	$KN(SiMe_3)_2$	40	33	76:24
14	**1a**	PPh3	KOH	40	0	-

[a] Conditions: **1** (0.1 mmol), Pd(dba)$_2$ (0.07 equiv), ligand (0.14 equiv), base (1.2 equiv), dry toluene, 100 °C, 56 h. [b] Yield of **TWGs** (mixed diastereoisomers). [c] Yield of debromination product **2**. [d] Yield of dimerization product **3**. [e] *Dr* ratio determined by ^{1}H NMR. [f] 1**b** is 2-bromo-9-octyl-9*H*-fluorene.

The stereoselectivity of TWGs was confirmed through two methods. Firstly, TWGs were formed with an 82:18 *cis-trans*-TWG1/*cis-cis*-TWG1 selectivity, as determined by 1H NMR (Supplementary Fig. 1). The ^{1}H NMR spectra of *cis-trans*-TWG1 and *cis-cis*-TWG1 in Supplementary Figure 2 indicated distinctive peaks. In the ^{1}H NMR spectra of *cis-trans*-TWG1, a singlet peak at 6.00 ppm corresponded to a single hydrogen, belonging to proton 8. Conversely, in the ^{1}H NMR spectra of *cis-cis*-TWG1, proton 8 resonated as a singlet at 8.19 ppm, integrating for three hydrogens. Comparing the ^{1}H NMR spectra of *cis-trans*-TWG1, TWGs, and *cis-cis*-TWG1 in Supplementary Fig. 3, the singular peaks at 6.00 ppm and 8.19 ppm in the ^{1}H NMR spectra of TWGs were characteristic of proton 8 in *cis-trans*-TWG1 and cis-cis-TWG1, respectively. In the ^{1}H NMR spectrum of TWGs, when the integral of the singlet at 8.19 ppm represented three hydrogens, the integral of the singlet at 6.00 ppm corresponded to 4.56 hydrogens. Consequently, the ratio of *cis-trans*-TWG1 to *cis-cis*-TWG1 existing in TWGs, calculated from the ^{1}H NMR of TWGs, was 4.56:1. Thus, the *cis-trans*-TWG1 selectivity was calculated as 4.56/ (1+4.56) = 0.82, and the *cis-cis*-TWG1 selectivity was calculated as 1/ (1+4.56) = 0.18. Hence, TWGs were formed with an 82:18 *cis-trans*-TWG1/*cis-cis*-TWG1 selectivity.

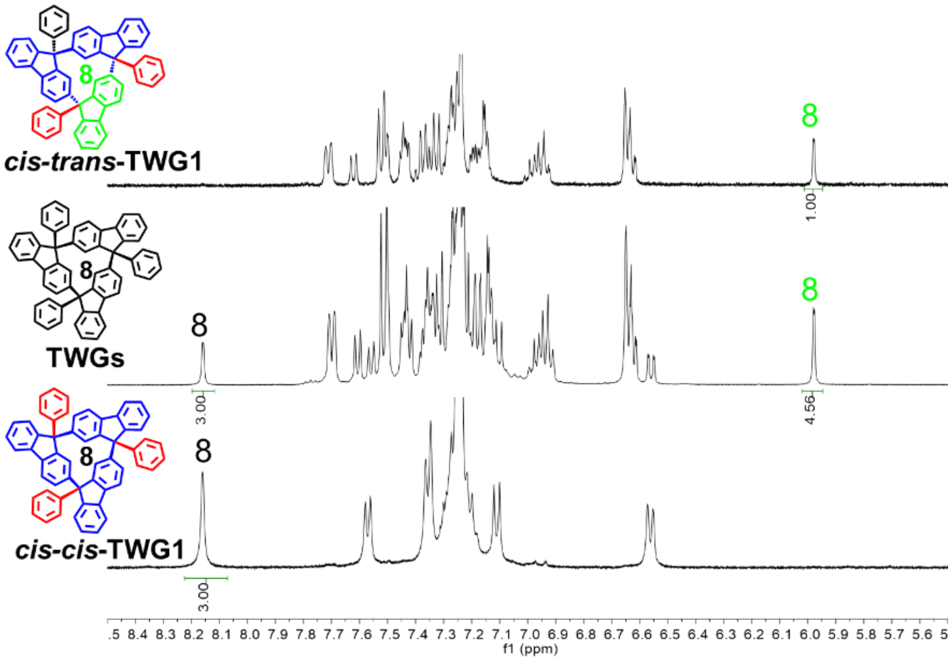

Supplementary Fig. 1 | ^{1}H NMR spectra of *cis-trans*-TWG1, TWGs and *cis-cis*-TWG1.

Secondly, TWGs were formed with an 87:13 *cis-trans*-TWG1/*cis-cis*-TWG1 selectivity, as determined by chiral HPLC (Supplementary Fig. 2). In the chiral HPLC spectrum of TWGs, it was evident that TWGs comprised (52.269+34.532):(6.347+6.652) = 87:13 *cis-trans*-TWG1/*cis-cis*-TWG1 selectivity. This selectivity closely mirrored the findings obtained through ^{1}H NMR analysis. Furthermore, the two enantiomers of *cis-trans*-TWG1 exhibited some enantioselectivity (52.269% and 34.532%, respectively), whereas the content of the two enantiomers of *cis-cis*-TWG1 remained essentially identical (6.347% and 6.852%, respectively).

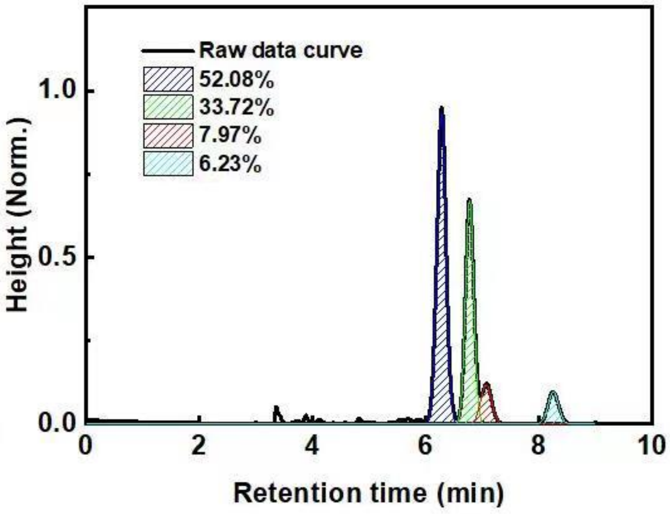

Supplementary Fig. 2 | Chiral HPLC chromatogram of TWGs.

Supplementary Table 2 | Separation method of TWGs in HPLC.

**Column**	**CHIRALPAK IG IG00CE-UC054**
**Column size**	**0.46 cm I.D. × 25 cm L**
**Injection**	**2 ul**
**Mobile phase**	**dichloromethane / methanol =20/80(V/V)**
**Flow rate**	**1.0 ml/min**
**Wave length**	**UV 254 nm**
**Temperature**	**room temperature**
**Sample name**	**mixture**
**Solution**	**1 mg/ml in dichloromethane**

Section 2. Theoretical calculation datas of TWG-TS1 and TWG-TS2

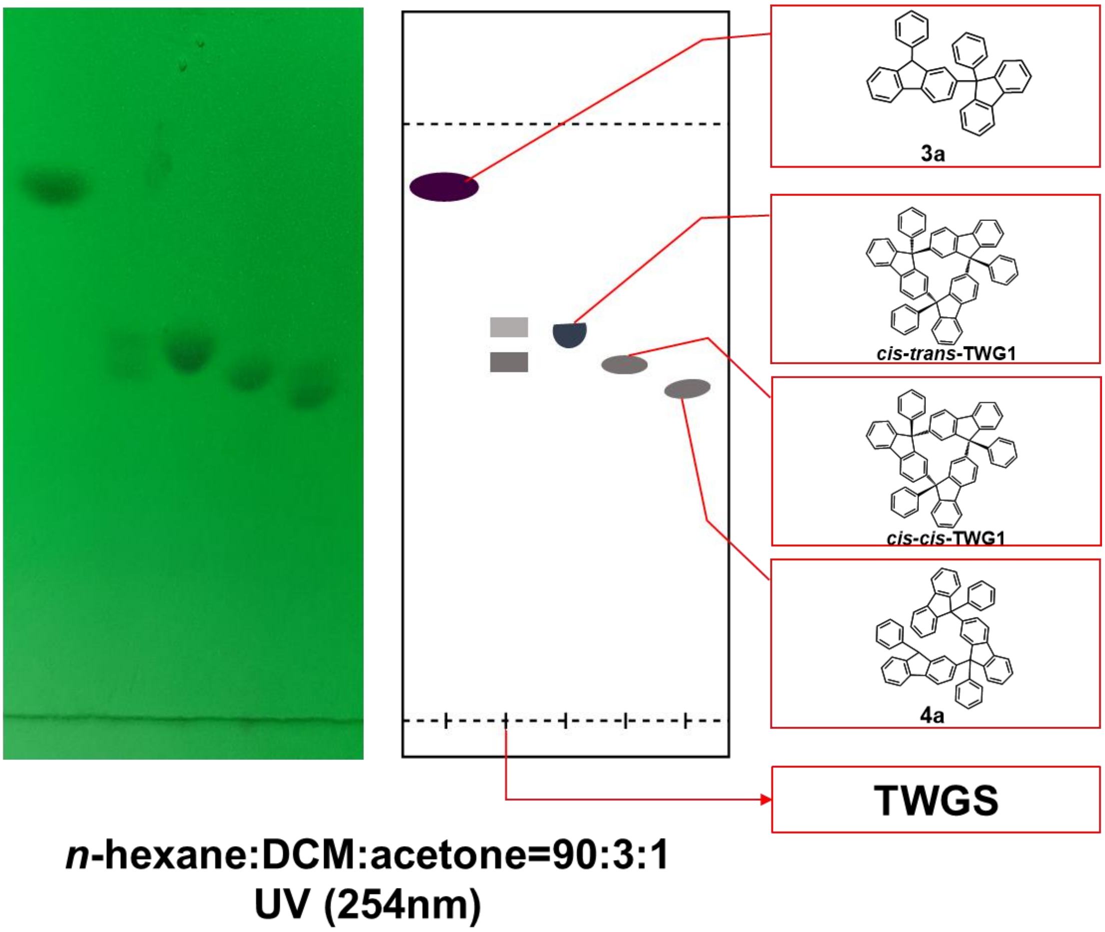

Supplementary Fig. 3 | The thin layer chromatography (TLC) of TWGs.

Initially, TWGs were purified via column chromatography [using silica gel with a petroleum to dichloromethane eluent ratio of 8:1]. Subsequently, *cis-trans*-TWG1 was obtained by recrystallizing TWGs with dichloromethane, followed by multiple washes of the recrystallized sample with n-hexane (5-8 times).

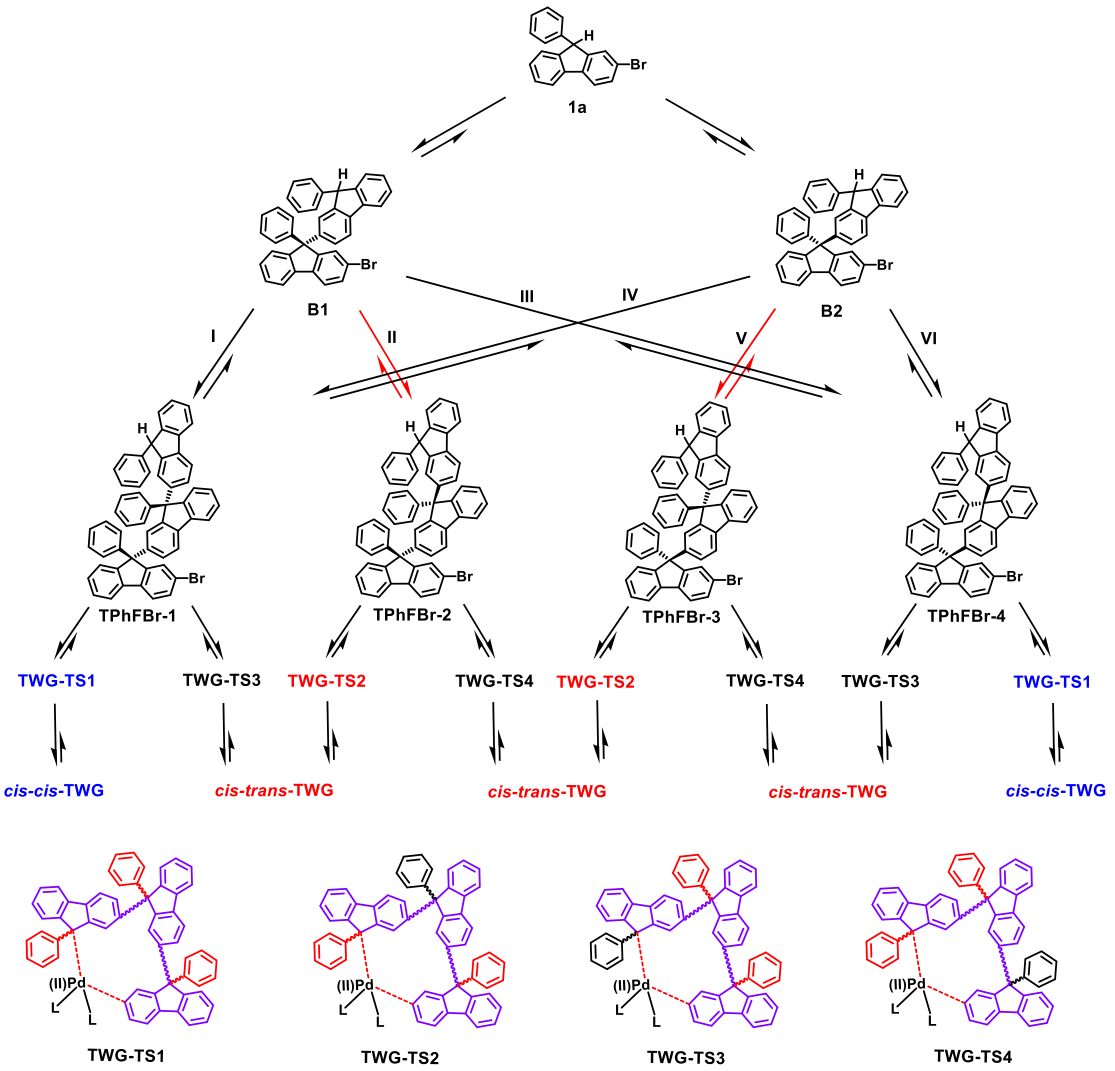

Supplementary Fig. 4 | The proposed buildup scheme of TWG and the structural formula of transition states.

Section 3. Structural characterization of DWGs and TWGs.

Elemental Composition Report **Page 1**

Single Mass Analysis
Tolerance = 5.0 mDa / DBE: min = -1.5, max = 50.0
Element prediction: Off
Number of isotope peaks used for i-FIT = 3

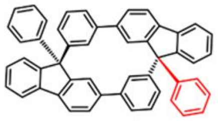

meso-DWG1

Monoisotopic Mass, Even Electron Ions
1 formula(e) evaluated with 1 results within limits (up to 50 best isotopic matches for each mas
Elements Used:
C: 31-58 H: 31-37
GR-3 371 (2.666)
1: TOF MS ES+

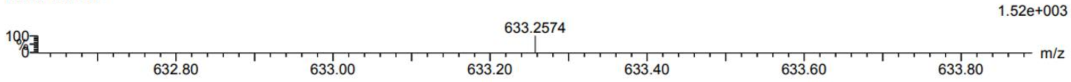

Minimum:				-1.5				
Maximum:		5.0	10.0	50.0				
Mass	Calc. Mass	mDa	PPM	DBE	i-FIT	Norm	Conf(%)	Formula
633.2574	633.2582	-0.8	-1.3	34.5	26.1	n/a	n/a	C50 H33

Elemental Composition Report **Page 1**

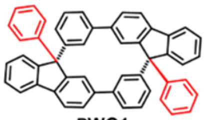

rac-DWG1

Single Mass Analysis
Tolerance = 5.0 mDa / DBE: min = -1.5, max = 50.0
Element prediction: Off
Number of isotope peaks used for i-FIT = 3

Monoisotopic Mass, Even Electron Ions
1 formula(e) evaluated with 1 results within limits (up to 50 best isotopic matches for each mass)
Elements Used:
C: 31-58 H: 31-37
GR-4 443 (3.179)
1: TOF MS ES+

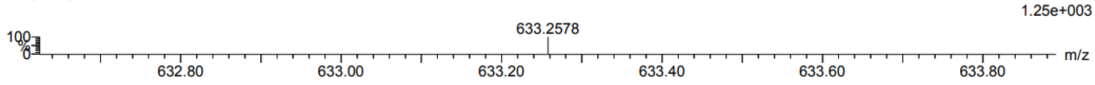

Minimum:				-1.5				
Maximum:		5.0	10.0	50.0				

Mass	Calc. Mass	mDa	PPM	DBE	i-FIT	Norm	Conf(%)	Formula
633.2578	633.2582	-0.4	-0.6	34.5	25.6	n/a	n/a	C50 H33

Supplementary Fig. 5 | HRMS spectra of *meso*-DWG1 and *rac*-DWG1.

Elemental Composition Report **Page 1**

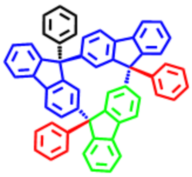

cis-*trans*-TWG1

Single Mass Analysis
Tolerance = 5.0 mDa / DBE: min = -1.5, max = 50.0
Element prediction: Off
Number of isotope peaks used for i-FIT = 3

Monoisotopic Mass, Even Electron Ions
1 formula(e) evaluated with 1 results within limits (up to 50 best isotopic matches for each mass)
Elements Used:
C: 31-58 H: 31-37 Na: 1-1

GR-1 259 (1.867)
1: TOF MS ES+

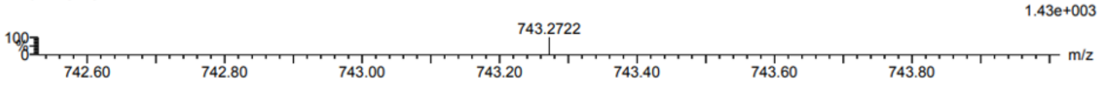

Minimum:				-1.5				
Maximum:		5.0	10.0	50.0				

Mass	Calc. Mass	mDa	PPM	DBE	i-FIT	Norm	Conf(%)	Formula
743.2722	743.2715	0.7	0.9	39.5	26.2	n/a	n/a	C57 H36 Na

Elemental Composition Report **Page 1**

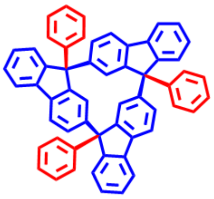

cis-*cis*-TWG1

Single Mass Analysis
Tolerance = 5.0 mDa / DBE: min = -1.5, max = 50.0
Element prediction: Off
Number of isotope peaks used for i-FIT = 3

Monoisotopic Mass, Even Electron Ions
1 formula(e) evaluated with 1 results within limits (up to 50 best isotopic matches for each mass)
Elements Used:
C: 31-58 H: 31-37
GR-2 435 (3.122)
1: TOF MS ES+

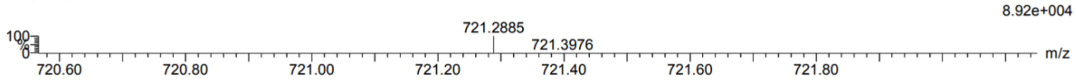

Minimum:				-1.5				
Maximum:		5.0	10.0	50.0				

Mass	Calc. Mass	mDa	PPM	DBE	i-FIT	Norm	Conf(%)	Formula
721.2885	721.2895	-1.0	-1.4	39.5	51.5	n/a	n/a	C57 H37

Supplementary Fig. 6 | HRMS spectra of *cis*-*trans*-TWG1 and *cis*-*cis*-TWG1.

Elemental Composition Report **Page 1**

Single Mass Analysis
Tolerance = 5.0 mDa / DBE: min = -1.5, max = 50.0
Element prediction: Off
Number of isotope peaks used for i-FIT = 3

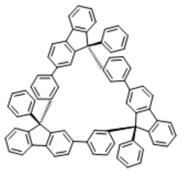

cis-trans-PhTWG8

Monoisotopic Mass, Even Electron Ions
9 formula(e) evaluated with 0 results within limits (up to 50 best isotopic matches for each mass)
Elements Used:
C: 0-100 H: 0-100
GR-5 284 (2.045)
1: TOF MS ES+

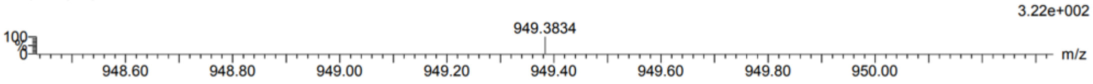

Minimum:				-1.5				
Maximum:		5.0	10.0	50.0				
Mass	Calc. Mass	mDa	PPM	DBE	i-FIT	Norm	Conf(%)	Formula
949.3834	---							

Elemental Composition Report **Page 1**

Single Mass Analysis
Tolerance = 5.0 mDa / DBE: min = -1.5, max = 50.0
Element prediction: Off
Number of isotope peaks used for i-FIT = 3

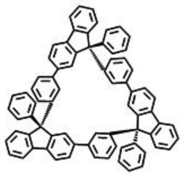

cis-cis-PhTWG8

Monoisotopic Mass, Even Electron Ions
9 formula(e) evaluated with 0 results within limits (up to 50 best isotopic matches for each mass)
Elements Used:
C: 0-100 H: 0-100 Na: 1-1

GR-6 346 (2.487)
1: TOF MS ES+

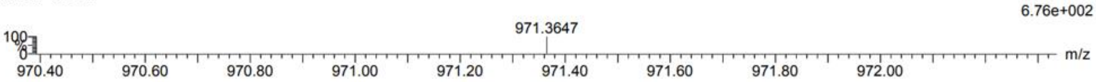

Minimum:				-1.5				
Maximum:		5.0	10.0	50.0				
Mass	Calc. Mass	mDa	PPM	DBE	i-FIT	Norm	Conf(%)	Formula
971.3647	---							

Supplementary Fig. 7 | HRMS spectra of *cis-trans*-TWG8 and *cis-cis*-TWG8.

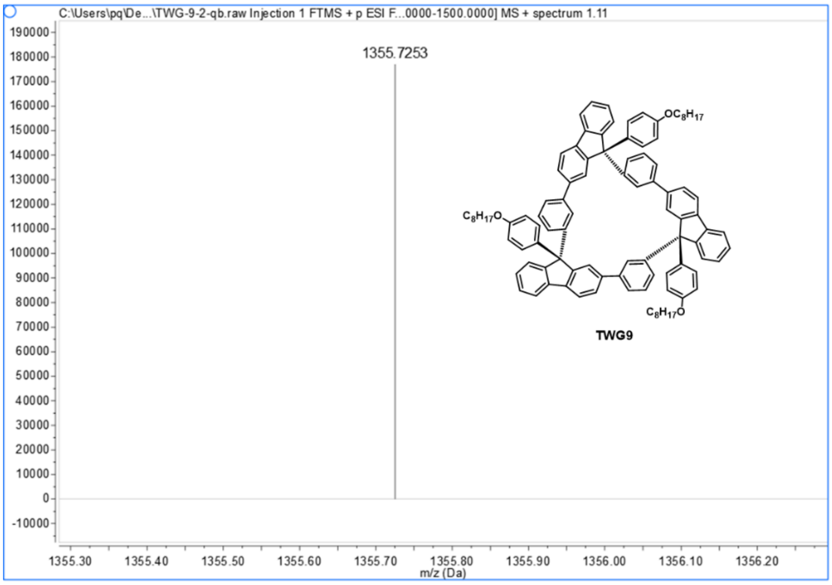

Supplementary Fig. 8 | HRMS spectrum of TWG9.

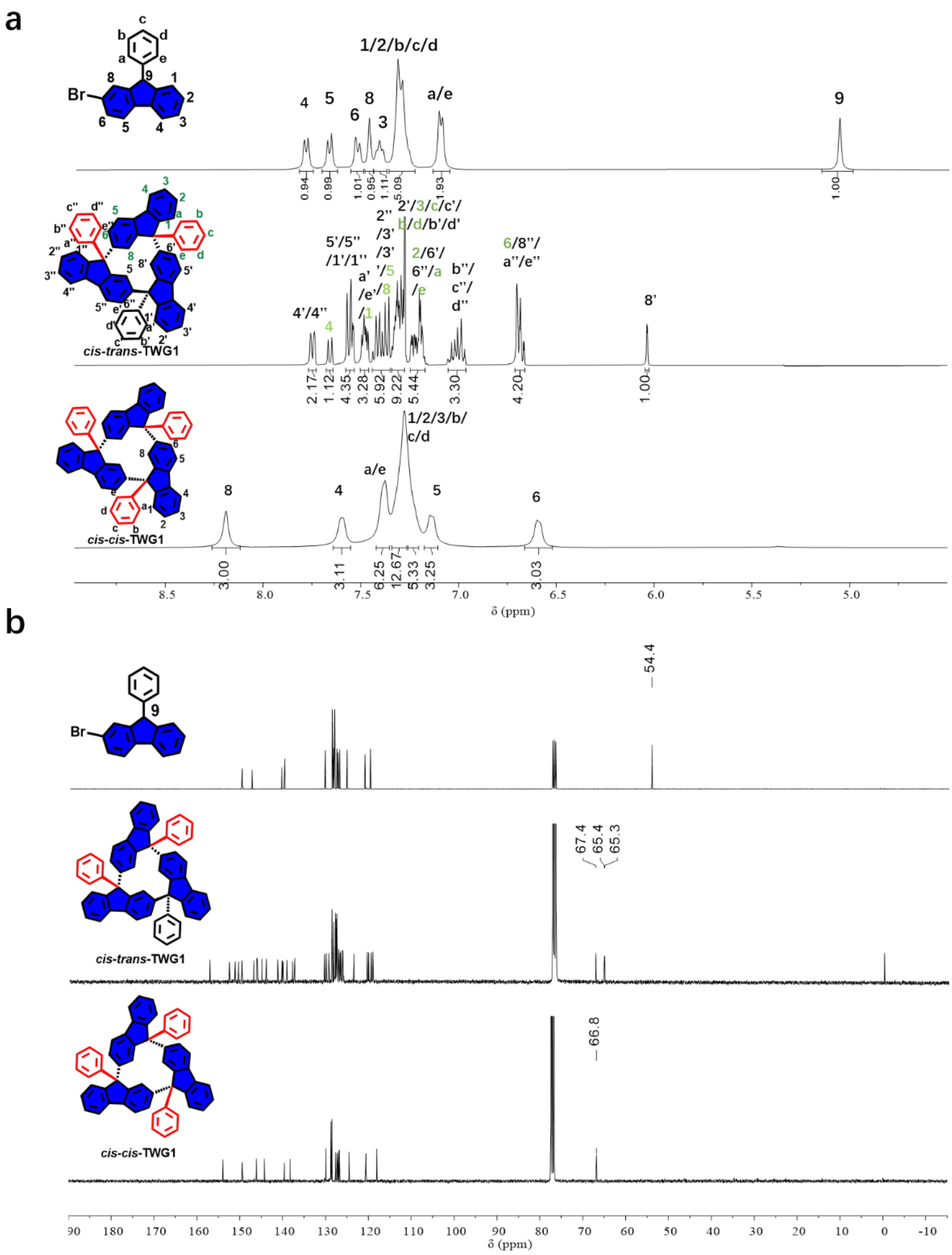

Supplementary Fig. 9 | (a) ^{1}H NMR spectra of 1a, *cis-trans*-TWG1 and *cis-cis*-TWG1; (b) ^{13}C NMR spectra of 1a, *cis-trans*-TWG1 and *cis-cis*-TWG1.

The ^{1}H NMR spectrum of *cis-trans*-TWG1, being an asymmetrical structure, exhibits complex resonances. In detail, in the ^{1}H NMR spectrum of **1a**, a doublet at 7.78-7.76 ppm corresponds to one hydrogen and is attributed to proton **4** of the fluorene moiety. Consequently, in the ^{1}H NMR spectrum of *cis-trans*-TWG1, protons **4'** and **4''** of the fluorenes resonate as a doublet at 7.75-7.7 ppm, integrating for two hydrogens. Moreover, based on the observed cross-peaks at (7.77 ppm, 7.44 ppm) and (7.76 ppm, 7.39 ppm) in the ^{1}H-^{1}H COSY spectrum of *cis-trans*-TWG1, we infer that protons **3'** and **3''** are positioned within the 7.42-7.34 ppm range. Similarly, using cross-peaks at

(7.33 ppm, 7.44 ppm) and (7.40 ppm, 7.38 ppm), protons **2'** and **2''** are located at 7.32-7.28 ppm and 7.42-7.34 ppm, respectively. Further, the cross-peaks at (7.34 ppm, 7.59 ppm) and (7.39 ppm, 7.56 ppm) indicate that protons **1'** and **1''** are situated within the 7.56-7.52 ppm region. Additionally, proton **4** resonates as a doublet at 7.66-7.64 ppm, integrating for one hydrogen due to the reduced deshielding from the fluorene's bending mode. This suggests that proton **3** is located at 7.32-7.28 ppm, as supported by the cross-peak at (7.31 ppm, 7.67 ppm). Further assignment indicates proton **2** is positioned at 7.23-7.17 ppm based on the cross-peak at (7.30 ppm, 7.23 ppm), and proton **1** is found at 7.48-7.45 ppm due to the cross-peak at (7.49 ppm, 7.24 ppm). Additionally, protons **5'** and **5''** are assigned to the region of 7.56-7.52 ppm. Consequently, proton **6'** and **6''** are located at 7.23-7.17 ppm based on the cross-peak at (7.25 ppm, 7.58 ppm). However, due to the shielding effect by adjacent fluorene, proton **6** is assigned to the 6.68-6.64 ppm region, confirmed by the cross-peak at (6.69 ppm, 7.38 ppm) placing proton **5** at 7.42-7.34 ppm. For the phenyl connected at the 9-position of fluorene, protons **a'** and **e'** are assigned to the 6.68-6.64 ppm region due to shielding, while the multiplet at 7.03-6.95 ppm (integrating to three hydrogens) corresponds to protons **b''**, **c''**, and **d''**. Conversely, protons **a** and **e** are placed in the 7.48-7.45 ppm region, being deshielded by fluorene. Hence, proton **b'**, **c'**, and **d'** are positioned at 7.32-7.28 ppm based on the cross-peak at (7.51 ppm, 7.30 ppm). Moreover, protons **a''** and **e''** are found at 7.23-7.17 ppm. Additionally, protons **b**, **c**, and **d** are located in the 7.32-7.28 ppm region based on the cross-peak at 7.33-7.21 ppm. Lastly, in the ^{1}H NMR spectrum of **1a**, a singlet at 7.43 ppm corresponds to one hydrogen and is assigned to proton **8** of the fluorene. Consequently, in the ^{1}H NMR spectrum of *cis-trans*-TWG1, we attribute proton **8** to the 7.42-7.34 ppm region. Then, proton **8''** is assigned to the 6.68-6.64 ppm region due to shielding by the adjacent fluorene. Notably, the chemical shift of proton **8'** shifts to a highfield (singlet peak at 6.00 ppm) due to the strong shielding effect from the adjacent fluorene.

The ^{1}H NMR spectrum of *cis-cis*-TWG1 (C_3 symmetry) exhibits a single set of resonances for the phenyl fluorene units due to its symmetrical structure. Specifically, the doublet (representing three hydrogens) at 7.60-7.59 ppm corresponds to proton 4, owing to the strong deshielding effect from the adjacent phenyl group. Additionally, the region at 7.34-7.20 ppm is assigned to proton 3, based on the observed cross-peak at (7.34 ppm, 7.62 ppm) in the ^{1}H-^{1}H COSY spectrum of *cis-cis*-TWG1. Moreover, the doublet (six hydrogens) at 7.39-7.37 ppm is attributed to protons a and e, influenced by the deshielding effects of fluorene and the adjacent fluorene moiety. Consequently, protons b and d are assigned to the region at 7.34-7.20 ppm, as supported by the cross-peaks at (7.33 ppm, 7.41 ppm) and (7.30 ppm, 7.40 ppm). The doublet (comprising three hydrogens) at 6.60-6.57 ppm is assigned to proton 6, primarily due to the shielding effect from the adjacent fluorene. Furthermore, proton 5 is placed in the 7.14-7.12 ppm region based on the observed cross-peak at (7.16 ppm, 6.62 ppm). Notably, due to the double deshielding effects caused by two adjacent fluorene and two phenyl groups linked at the 9-position, the chemical shift value of proton 8 significantly shifts to 8.19 ppm. Finally, protons 1, 2, and c are associated with the region at 7.34-7.20 ppm in the spectrum.

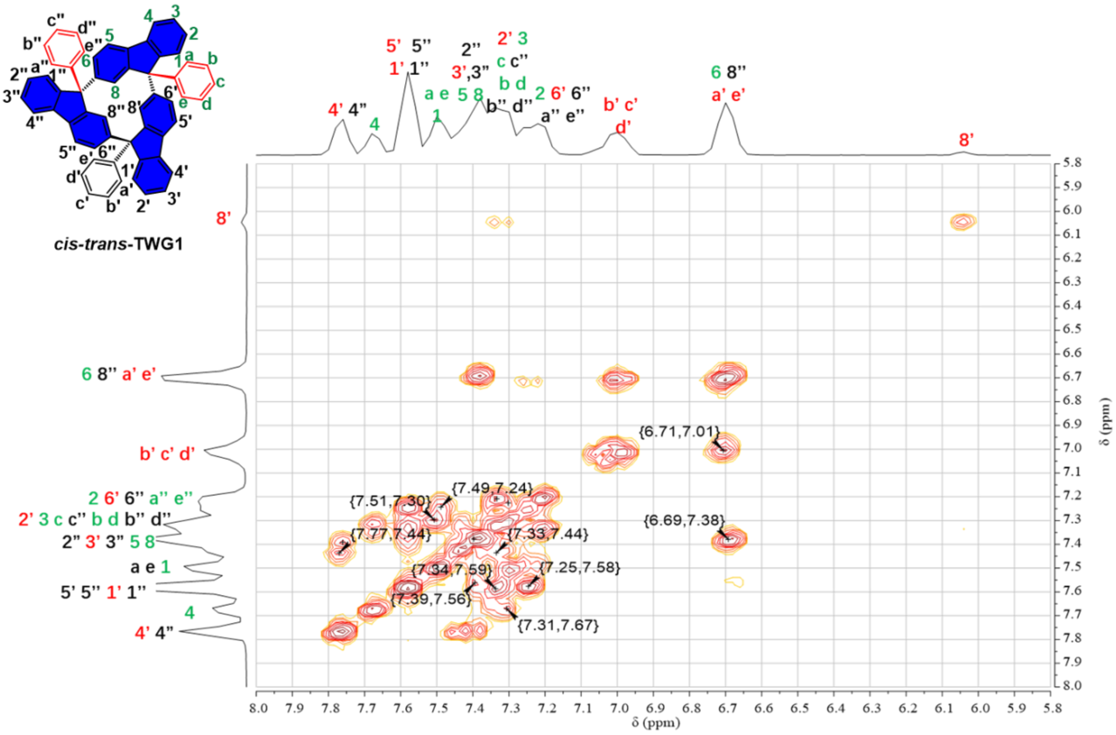

Supplementary Fig. 10 ｜^{1}H-^{1}H COSY spectrum of *cis-trans*-TWG1.

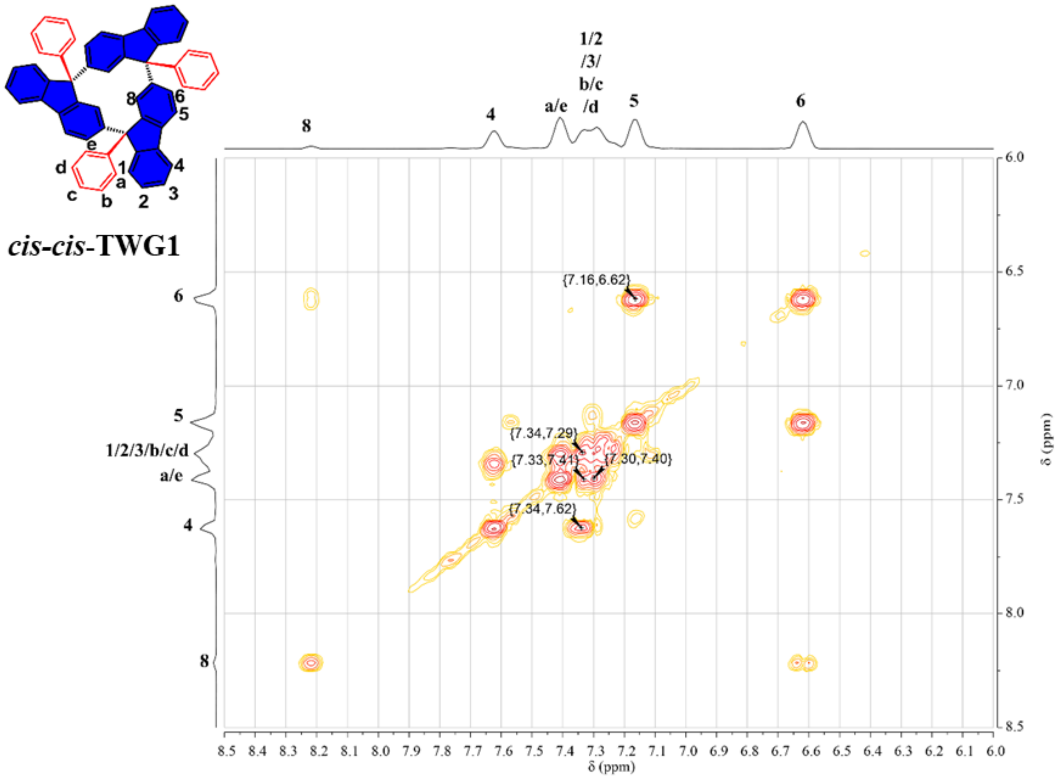

Supplementary Fig. 11 ｜^{1}H-^{1}H COSY spectrum of *cis-cis*-TWG1.

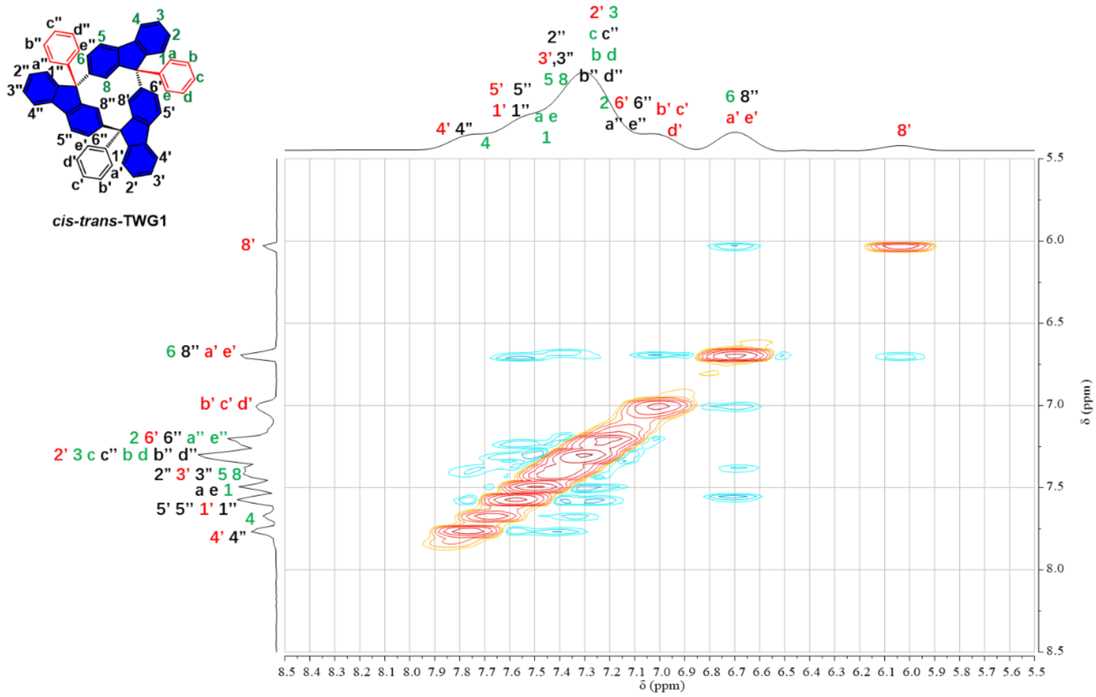

Supplementary Fig. 12 ｜ ^{1}H-^{1}H NOESY spectrum of *cis-trans*-TWG1.

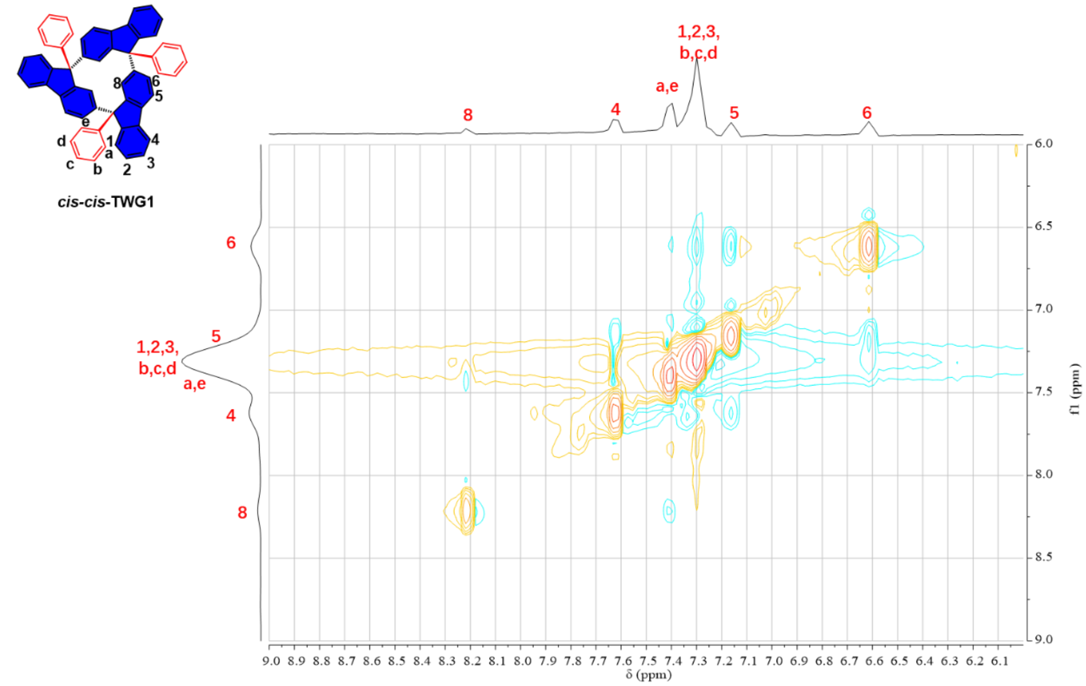

Supplementary Fig. 13 ｜ ^{1}H-^{1}H NOESY spectrum of *cis-cis*-TWG1.

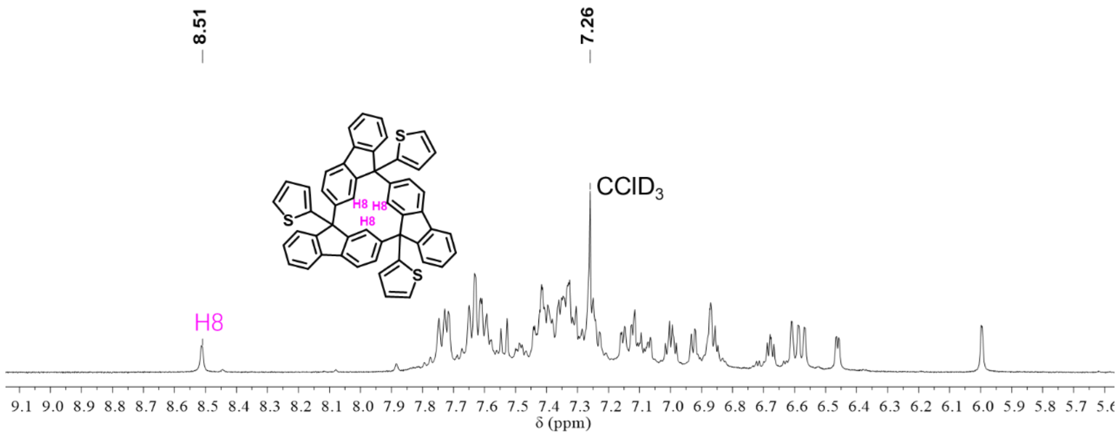

Supplementary Fig. 14 | ^{1}H NMR Spectra of TWG6.

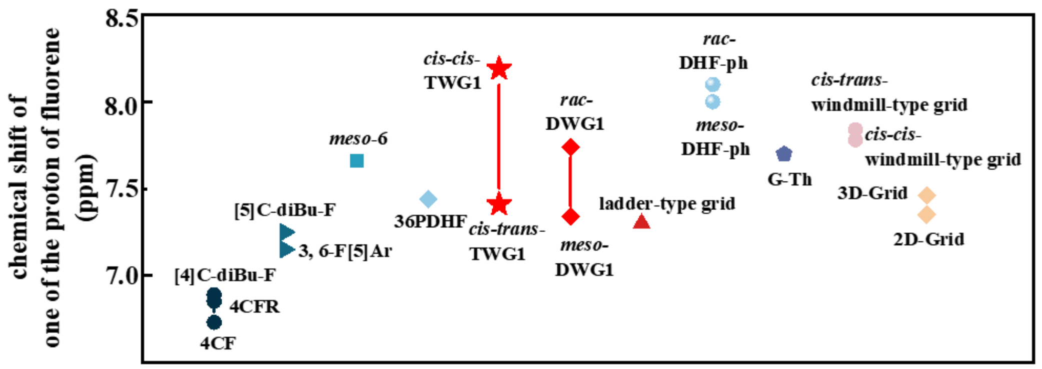

Supplementary Fig. 15 | Chemical shift of one of the proton of fluorene. [4]Cyclofluorenequare (4CF)[36]; [4]C-diBu-F[35]; [5]C-diBu-F[35] [2]spirobifluorenylenes (*meso*-6)[37]; tetrofluorene (36PDHF)[38]; 3D Grid;[39] 2D Grid;[39] windmill-type nanogrids[16]; ladder-type nanogrids[15]; fluorenes-based DHGs (DHGs-F)[17].

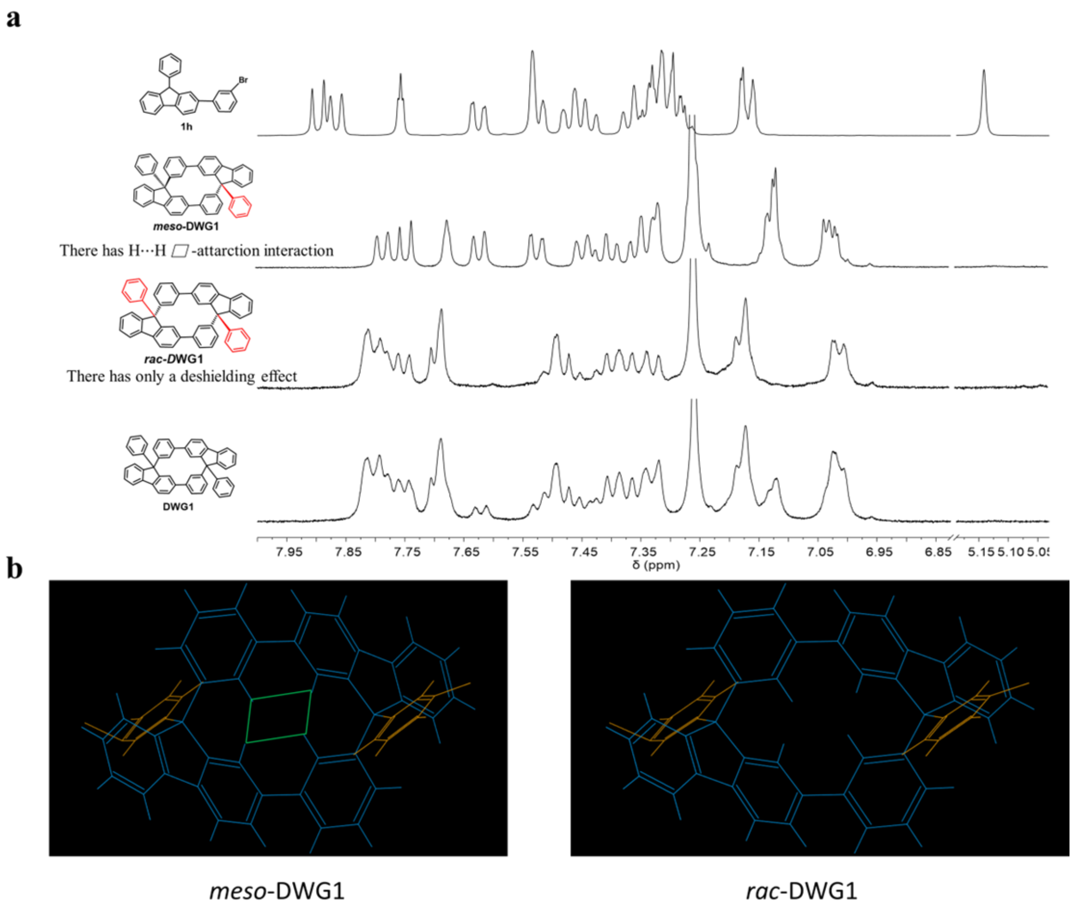

Supplementary Fig. 16 | (a) :^{1}H NMR spectra of 1h, *meso*-DWG1, *rac*-DWG1; (b) H···H ▱-attraction interaction (green) of *meso*-DWG1 and *rac*-DWG1. H···H ▱-attraction interattractionaction: we define a triangle formed by four stacking interactions as H···H ▱-attraction interaction. The structure of *meso*-DWG1 has only an H···H ▱-attraction interaction, while the structure of *rac*-DWG1 has only a deshielding effect.

Section 4. The crystal data of single crystal of *meso*-DWG1 and *rac*-DWG1 and *cis-trans*-TWG1 and *cis-cis*-TWG1 and co-crystal of *cis-cis*-TWG1 and *cis-trans*-TWG1.

Supplementary Table 3 | The displacement ellipsoids are drawn at the 50% probability level. Single crystals suitable for X-ray analysis were obtained by slow evaporation of dichloromethane mixed with isopropanol solvent.

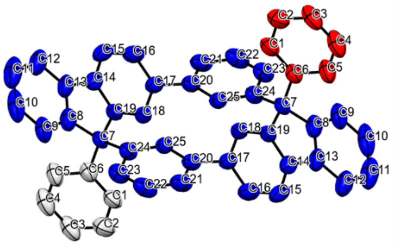

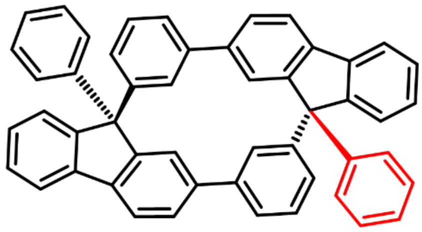

***meso*-DWG1**

Moiety formula	$C_{50}H_{32}$
CCDC	2248637
Bond precision	C-C = 0.0124 Å
Wavelength	1.34139
Cell	a=17.542(5) b=27.270(8) c=6.5789(19)<br>alpha=90 beta=90 gamma=90
Temperature	193K
Volume	3147.2(16)
Space group	Pccn
Hall group	-P 2ab 2ac
$D_{x,g\ cm-3}$	1.335
Z	4
M_u (mm-1)	0.365
F000	1328.0
h,k,l_{max}	21,32,7
Nref	2898
Tmin,Tmax	0.957,0.964
Correction method=<br># Reported T Limits	Tmin=0.465<br>Tmax=0.751
AbsCorr	MULTI-SCAN
Data completeness	0.994
Theta(max)	54.202

R(reflections)	0.1517(1937)
wR2(reflections)	0.3489(2882)
S	1.107
Npar	226

Supplementary Table 4 | The displacement ellipsoids are drawn at the 50% probability level. Single crystals suitable for X-ray analysis were obtained by slow evaporation of dichloromethane mixed with isopropanol solvent.

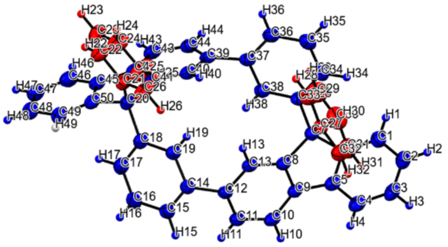

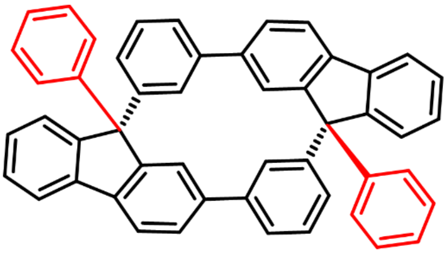

***rac*-DWG1**

Moiety formula	$C_{50}H_{32}$
CCDC	2248641
Bond precision	C-C = 0.052Å
Wavelength	1.54178
Cell	a=11.3411(2) b=12.2038(3) c=15.3609(3) alpha=108.401(1) beta=98.498(1) gamma=108.4621(1)
Temperature	193K
Volume	1839.58(7)
Space group	P-1
Hall group	-P -
$D_{x,g\ cm-3}$	1.296
Z	2
M_u (mm-1)	1.858
F000	748.0
h,k,l_{max}	13,14,18
Nref	6764
Tmin,Tmax	0.748,0.800
Correction method= # Reported T Limits	Tmin=0.377 Tmax=0.0.467
AbsCorr	MULTI-SCAN
Data completeness	0.994

Theta(max)	68.402
R(reflections)	0.0281(5077)
wR2(reflections)	0.2445(6722)
S	1.079
Npar	478

Supplementary Table 5 | The displacement ellipsoids are drawn at the 50% probability level. Single crystals suitable for X-ray analysis were obtained by slow evaporation of dichloromethane mixed with isopropanol solvent.

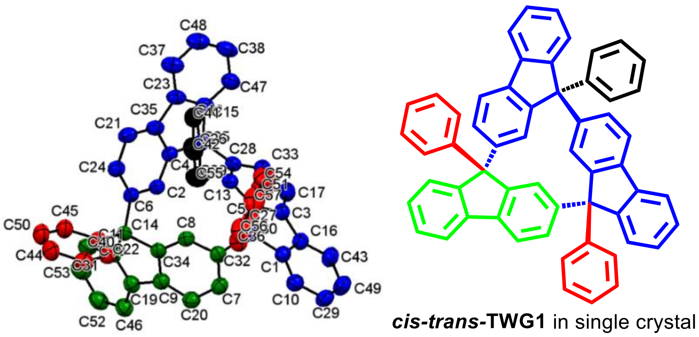

***cis-trans*-TWG1** in single crystal

Moiety formula	$C_{57}H_{36}$
CCDC	2052303
Bond precision	C-C = 0.0037 Å
Wavelength	1.34138
Cell	a=26.3387(7) b=20.6806(7) c=17.3390(5)<br>alpha=90 beta=110.254(2) gamma=90
Temperature	193K
Volume	8860.6(5)
Space group	C12/c1
Hall group	-C2yc
$D_{x,g\ cm-3}$	1.081
Z	8
M_u (mm-1)	0.299
F000	3024.0
h,k,l_{max}	31,24,20
Nref	8125
Tmin,Tmax	0.200,0.301
Correction method=<br># Reported T Limits	Tmin=0.200<br>Tmax=0.301
AbsCorr	MULTI-SCAN

Data completeness	0.994
Theta(max)	54.079
R(reflections)	0.0634(5887)
wR2(reflections)	0.1846(8125)
S	1.035
Npar	514

Supplementary Table 6 | The displacement ellipsoids are drawn at the 50% probability level. Single crystals suitable for X-ray analysis were obtained by slow evaporation of dichloromethane mixed with *n*-hexane solvent.

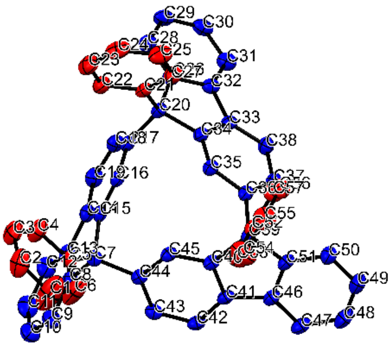

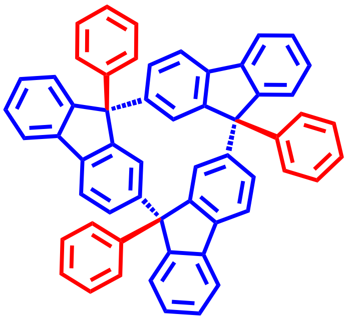

***cis*-*cis*-TWG1** in single crystal

Moiety formula	$C_{57}H_{36}$
CCDC	2124396
Bond precision	C-C = 0.0048 Å
Wavelength	0.71073
Cell	a=14.3732(7) b=11.6437(5) c=27.9984(15)<br>alpha=90 beta=103.558(2) gamma=90
Temperature	191K
Volume	4555.2(4)
Space group	P121/n1
Hall group	-P2yn
$D_{x,g\ cm-3}$	1.238
Z	4
M_u (mm-1)	0.183
F000	1780.0
h,k,l_{max}	18, 15, 36
Nref	10472
Tmin,Tmax	0.667, 0.746
Correction method=<br># Reported T Limits	Tmin= 0.667<br>Tmax=0.746

AbsCorr	MULTI-SCAN
Data completeness	0.998
Theta(max)	27.529
R(reflections)	0.0817 (6763)
wR2(reflections)	0.2367 (10472)
S	1.060
Npar	570

Supplementary Table 7 | The displacement ellipsoids are drawn at the 50% probability level. Single crystals suitable for X-ray analysis were obtained by slow evaporation of tetrahydrofuran mixed with water solvent.

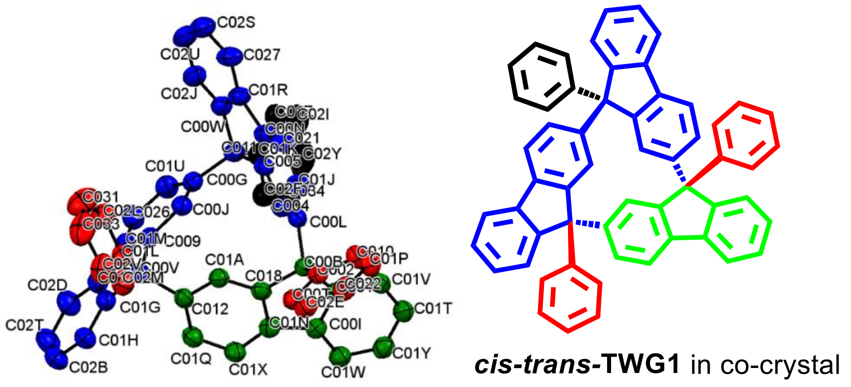

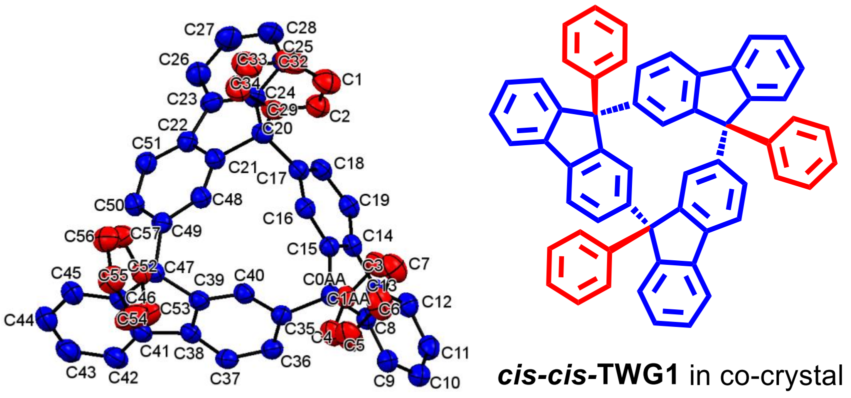

Moiety formula	$C_{114}H_{72}$
CCDC	2052304
Bond precision	C-C = 0.0031 Å
Wavelength	1.34138
Cell	a=26.9960(11) b=20.4249(9) c=17.8631(7)<br>alpha=90 beta=93.411(2) gamma=90
Temperature	193K
Volume	9832.1(7)
Space group	P121/c1

Hall group	-P 2ybc
$D_{x,g\ cm-3}$	0.974
Z	8
M_u (mm-1)	0.269
F000	3024.0
h,k,l_{max}	32,24,21
Nref	18003
Tmin,Tmax	0.213,0.301
Correction method= # Reported T Limits	Tmin=0.213 Tmax=0.301
AbsCorr	NONE
Data completeness	0.996
Theta(max)	53.994
R(reflections)	0.0522(11040)
wR2(reflections)	0.1500(18003)
S	0.958
Npar	1027

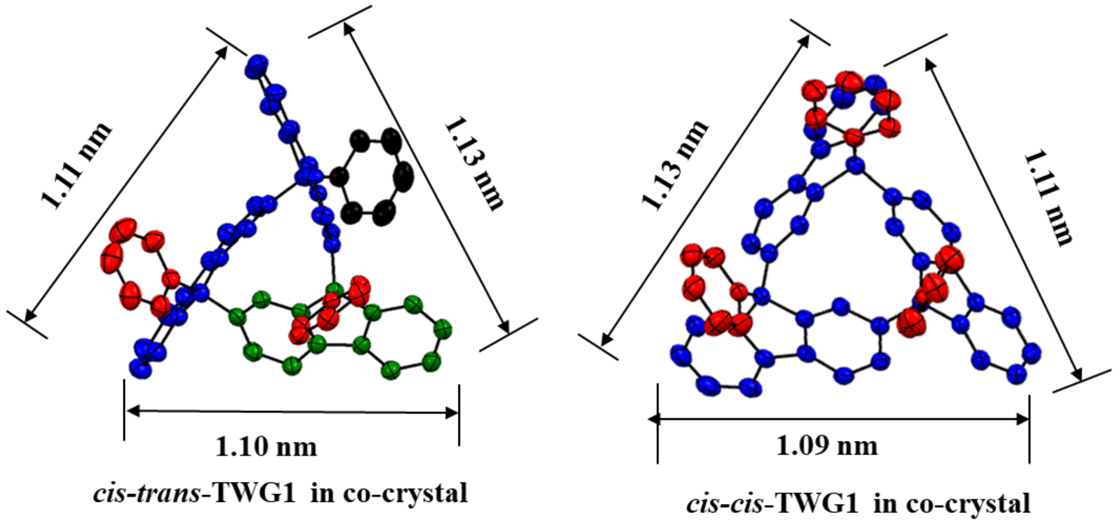

Supplementary Fig. 17 | co-crystallography of *cis-cis*-TWG1 and *cis-trans*-TWG1. (The red and black lines represent phenyl at the same side or the other side, respectively. The green lines exhibit the bent skeleton and blue lines exhibits the stretched skeleton.).

Supplementary Table 8 | Relevant H-H distances of the X-Ray structures of *cis-trans*-TWG1 and *cis-cis*-TWG1.

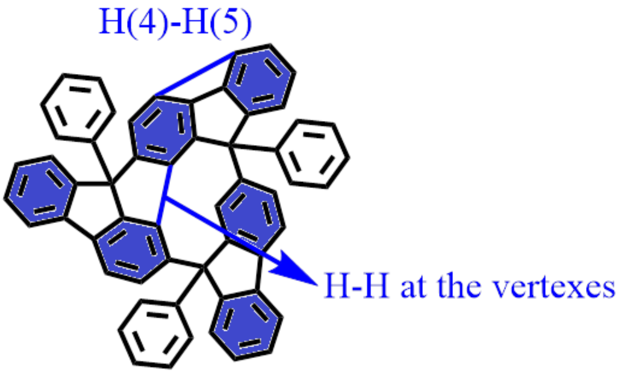

	H(4)-H(5)	H-H at the vertexes
***cis-trans*-TWG1 (single crystal)**	2.69 Å, 2.70 Å, 2.71 Å	3.10 Å, 2.18 Å, 3.55 Å
***cis-trans*-TWG1 (co-crystal)**	2.72 Å,2.70 Å, 2.67 Å	3.30 Å, 2.33 Å 3.60 Å
***cis-cis*-TWG1 (single crystal)**	2.72 Å，2.75 Å 2.70 Å	2.19 Å,2.19 Å, 2.31 Å
***cis-cis*-TWG1 (co-crystal)**	2.67 Å, 2.70 Å, 2.74 Å	2.15 Å, 2.19 Å, 2.34 Å

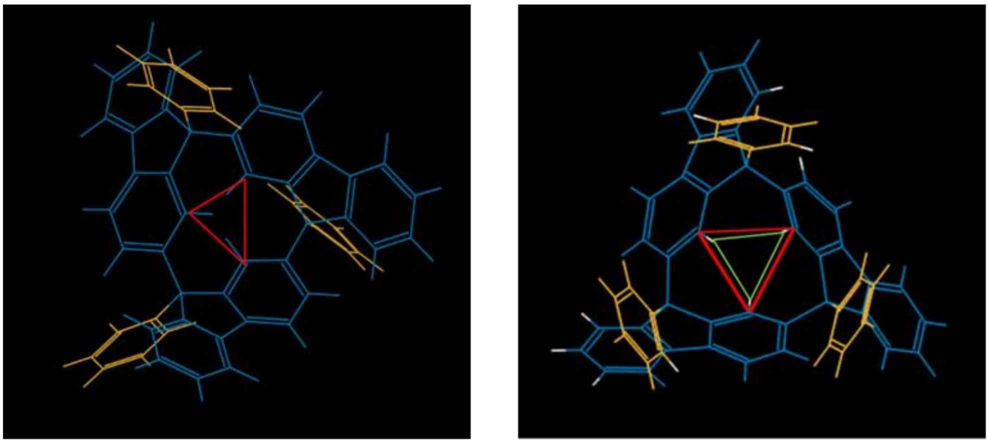

cis-trans-TWG1 *cis-cis*-TWG1

Supplementary Fig. 18 | H···H *Δ*-attraction interaction (green) and π···π *Δ*-repulsion interaction (red) of TWGs. Delta (*Δ*)-stacking motif: we define a triangle formed by three stacking interactions as delta-stacked motif, as shown in the figure. A symmetric H···H delta (*Δ*)-attraction interaction can be extracted with mutually three H···H interactions among the adjacent hydrogen at the 8-position of fluorenes. π···π *Δ*-repulsion interaction can be extracted with mutually three π···π stacking interactions on the vertexes between adjacent fluorenes.

Supplementary Table 9 | Relevant H-H distances of the X-Ray structures of *meso*-DWG1 and *rac*-DWG1.

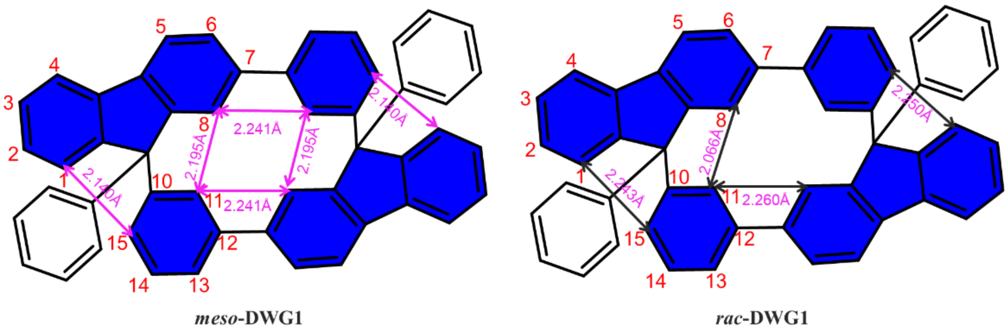

	H(1)-H(15)	H(8)-H(11)
***meso*-DWG1** **(single crystal)**	2.140 Å,2.140 Å	2.195 Å,2.195 Å, 2.241 Å,2.241 Å
***rac*-DWG1** **(single crystal)**	2.243 Å,2.250 Å	2.066 Å,2.260 Å

Supplementary Table 10 | Relevant C-H···π and π···π distances of the X-Ray structures of *cis-trans*-TWG1 and *cis-cis*-TWG1.

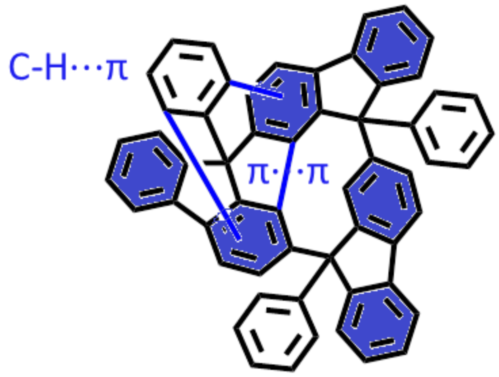

	C-H···π	π···π
***cis-trans*-TWG1** **(single crystal)**	3.18 Å, 3.59 Å, 3.38 Å, 3.37 Å, 4.83 Å, 4.06 Å	3.04 Å, 3.08 Å, 3.18 Å
***cis-trans*-TWG1** **(co-crystal)**	3.33 Å, 3.66 Å 3.31 Å, 4.74 Å 3.98 Å, 3.17 Å	3.05 Å, 3.05 Å, 3.24 Å

***cis-cis*-TWG1** (single crystal)	3.44 Å, 3.44 Å, 3.44 Å, 3.47 Å 3.47 Å, 3.37 Å	3.06 Å, 3.06 Å, 3.05 Å
***cis-cis*-TWG1** (co-crystal)	3.39 Å, 3.47 Å, 3.49 Å, 3.37 Å, 3.45 Å, 3.50 Å	3.04 Å, 3.05 Å, 3.08 Å

Supplementary Table 11 | Relevant C-H···π distances of the X-Ray structures of *meso*-DWG1 *and rac*-DWG1.

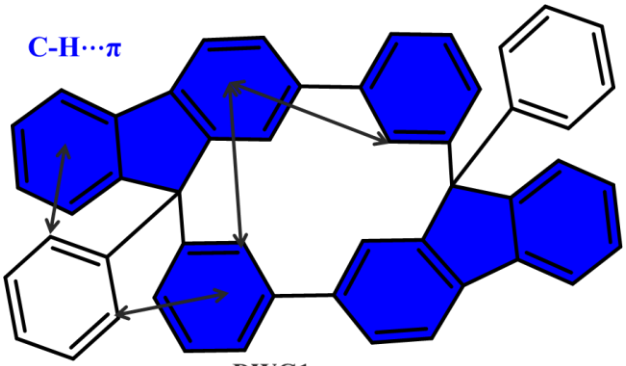

DWG1

	C-H···π
***meso*-DWG1** (single crystal)	2.57 Å，2.51 Å，2.78 Å, 2.55 Å 2.80 Å，2.77 Å，2.90 Å，2.50 Å，2.24 Å，2.56 Å，2.76 Å
***rac*- DWG1** (single crystal)	2.57 Å, 2.51 Å, 2.78 Å,2.64 Å,2.56 Å, 2.62 Å, 2.78 Å, 2.89 Å, 2.45 Å, 2.38 Å, 2.69 Å, 2.51 Å, 2.23 Å, 2.28 Å, 2.72 Å,2.88 Å，2.74 Å

Supplementary Table 12 | Relevant π···π distances of the X-Ray structures of *meso*-DWG1 *and rac*-DWG1.

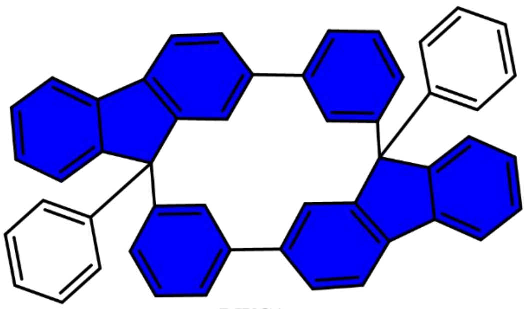

meso-DWG1

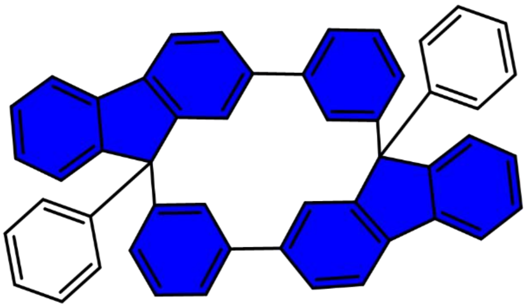

rac-DWG1

	π···π
***meso*-DWG1 (single crystal)**	3.326 Å， 3.028 Å,3.326 Å,3.028 Å
***rac*- DWG1 (single crystal)**	3.27 Å,.33 Å,3.39 Å,3.03 Å,3.06 Å 3.27 Å,3.33 Å,3.39 Å 3.40 Å,3.39 Å

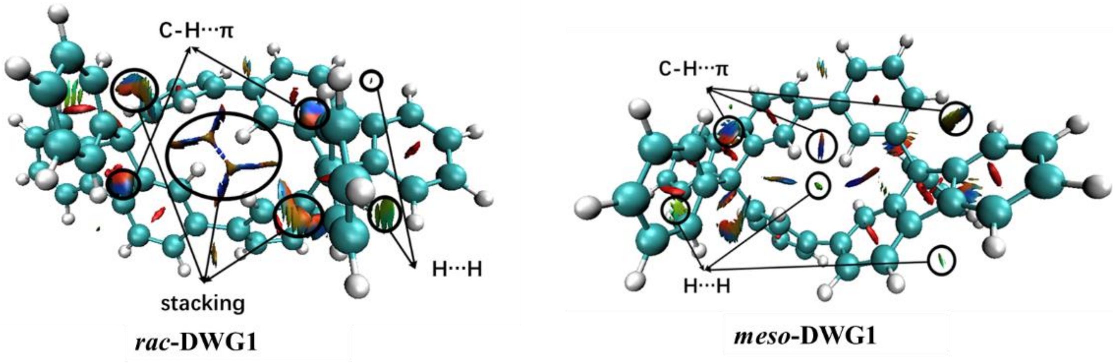

Supplementary Fig. 19 ｜The non-covalent interactions (NCI) isosurfaces for the *rac*-DWG1 and *meso*-DWG1.

All the quantum chemical calculations were performed using Gaussian 09 Program package[4]. The interactions encompass H-bonds (highlighted in blue) as strong interactions, van der Waals interactions (highlighted in green), and steric repulsions (highlighted in red) within the fluorenes, along with H···H interactions between the benzene ring and fluorene molecules. In the scatter plot of rac-DWG1, the spikes corresponding to the two H···H interactions were approximately around ±0.005 a.u., while those of meso-DWG1 were approximately around ±0.006 a.u. Similarly, C-H···π interactions were categorized into two types: interactions between fluorene and the benzene ring, and interactions between two benzene rings. In the scatter plot of *rac*-DWG1, the corresponding spikes were primarily located around ±0.012 a.u., while for *meso*-DWG1, they were mainly around ±0.011 a.u. Furthermore, the stacking motif includes H···H interaction and C-H···π interaction. The scatter plots of *rac*-DWG1 indicate that the spike positions corresponding to stacking motifs were approximately around ±0.011 a.u.

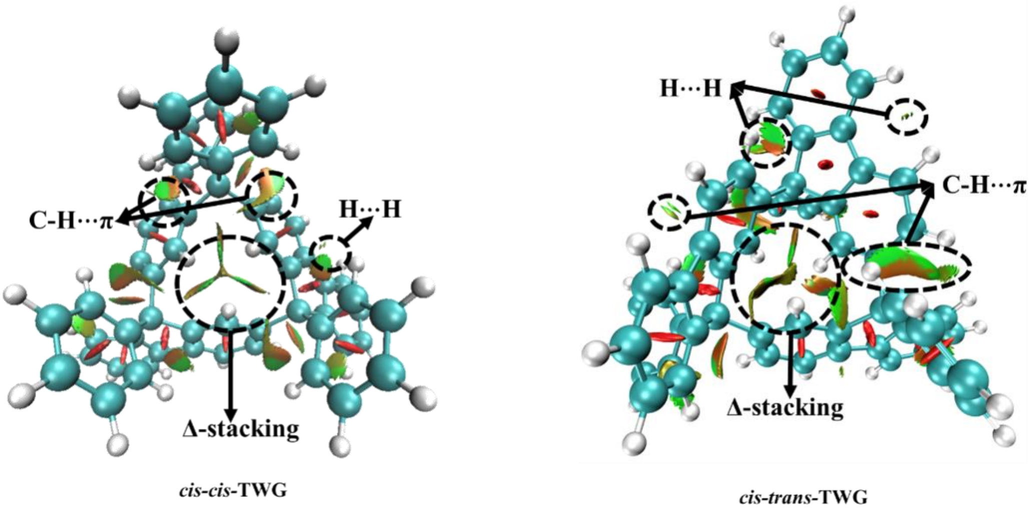

Supplementary Fig. 20 | The non-covalent interactions (NCI) isosurfaces for the *cis-trans*-TWG1 and *cis-cis*-TWG1.

The interactions depicted encompass H-bonds (highlighted in blue) as strong interactions, van der Waals interactions (highlighted in green), and steric repulsions (highlighted in red) within the fluorenes, along with H···H interactions between fluorene molecules. In the scatter plot of *cis-cis*-TWG1, the spikes representing all three H···H interactions were approximately around ±0.04 a.u., whereas those of *cis-trans*-TWG1 were around ±0.05 a.u.. Similarly, C-H···π interactions were categorized into two types: interactions between fluorene and the benzene ring, and interactions between two fluorene molecules. In the scatter plot of *cis-cis*-TWG1, the corresponding spikes primarily appeared around ±0.012 a.u., while for *cis-trans*-TWG1, they were mainly located around ±0.017 a.u.. Additionally, the *Δ*-stacking motif includes H···H *Δ*-attraction interaction and π···π *Δ*-repulsion interaction. The scatter plots of *cis-cis*-TWG1 and *cis-trans*-TWG1 indicate that the spike positions corresponding to *Δ*-stacked motifs were approximately around ±0.01 a.u..

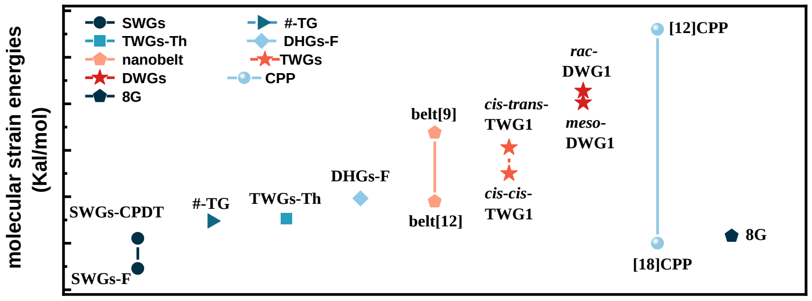

Supplementary Fig. 21 | Summary of MSE for other nanogrids nanorbelt and CPPs.

Supplementary Table 13 | Angle strain of DWGs1 is defined as relevant angles of SP^3 including the measurements of X-Ray structures of *meso*-DWG1and *rac*-

DWG1.

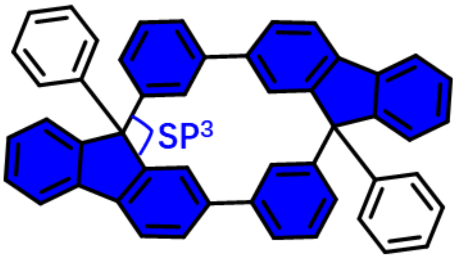

	SP^3	Average angle strain
***meso*-DWG1 (single crystal)**	113.04°	3.54°
***rac*-DWG1 (single crystal)**	110.29°<br>112.22°	1.76°

Supplementary Table 14 | Angle strain of TWGs is defined as relevant angles of SP^3 including the measurements of X-Ray structures of *cis-trans*-TWG1 and *cis-cis*-TWG1.

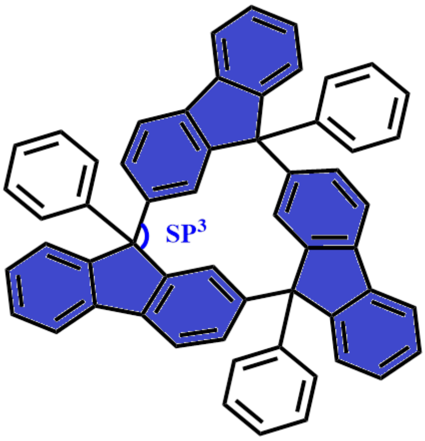

	SP^3	Average angle strain
***cis-trans*-TWG1 (single crystal)**	106.05°, 108.46°, 109.56°	1.44°
***cis-trans*-TWG1 (co-crystal)**	105.28°, 107.21°, 108.82°	2.36°
***cis-cis*-TWG1 (single crystal)**	106.75°, 106.22°, 104.13°	3.77°
***cis-cis*-TWG1 (co-crystal)**	104.71°, 105.96°, 107.01°	3.57°

Definition of bending strain: The bending strain is defined as the absolute difference in the C2-C11 distance between DWG1 and normal m-BrPhF, as determined by single crystal XRD. Meanwhile, the out-of-plane distortion denotes the distance between carbon atoms C2 and C11 situated within the same m-BrPhF unit.

Supplementary Table 15 | Relevant C2-C11 distances of the X-Ray structures of DWG1

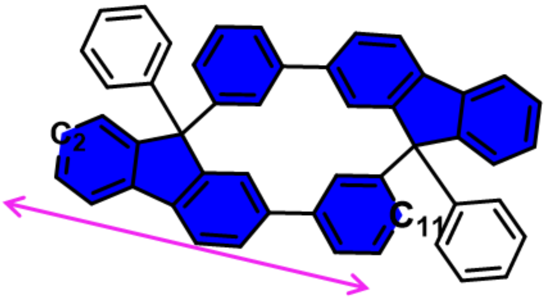

	C2-C11	average	Bending strain
***meso*-DWG1 (single crystal)**	10.914 Å<br>10.914 Å	10.914 Å	0.16 Å
***rac*-DWG1 (single crystal)**	10.946 Å,<br>10.942 Å	10.944 Å	0.19 Å

Definition of bending strain: The bending strain refers to the absolute difference in the C2-C7 distance between TWGs and normal fluorene, as measured by single crystal XRD. The out-of-plane distortion represents the distance between carbon atoms C2 and C7 localized on the same fluorene unit.

Supplementary Table 16 | Relevant C2-C7 distances of the X-Ray structures of *cis-trans*-TWG1 and *cis-cis*-TWG1.

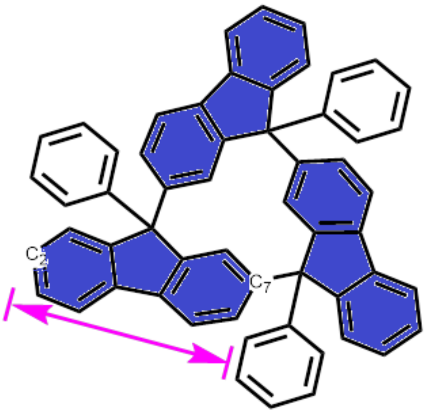

	**C2-C7**	**average**	**Bending strain**
***cis-trans*-TWG1 (single crystal)**	6.89 Å, 6.87 Å, 6.76 Å	6.84 Å	0.01 Å
***cis-trans*-TWG1 (co-crystal)**	6.88 Å, 6.84 Å, 6.89 Å	6.87 Å	0.02 Å

cis-cis-TWG1 (single crystal)	6.90 Å, 6.91 Å, 6.93 Å	6.91 Å	0.06 Å
cis-cis-TWG1 (co-crystal)	6.90 Å, 6.90 Å, 6.91 Å	6.90 Å	0.05 Å

Definition of a torsion angle: The external torsion angle (θ1 and θ2) is the dihedral angle between fluorene and two adjacent backbone phenyl.

Supplementary Table 17 | Relevant external torsion angles of the X-Ray structures of DWG1.

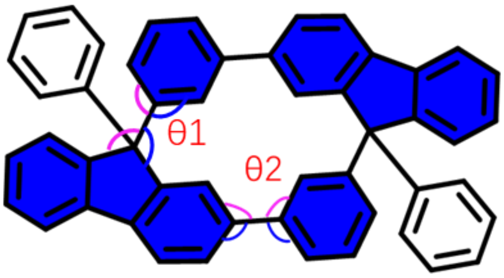

	θ1	Average (°)	θ2	Average (°)
***meso*-DWG1 (single crystal)**	57.77°,17.39°, 57.77°,17.39°,	37.58	33.74°,36.49°, 36.49°,33.74°,	35.115
***rac*-DWG1 (single crystal)**	40.23,31.28, 64.94，5.01	35.37	60.65°,49.07°, 34.33°,39.68°,	35.15

Definition of a torsion angle: The external torsion angle (θ_{ext}) is the dihedral angle between two fluorene building units. Two angles are measured for each C7-C9 link.

Supplementary Table 18 | Relevant external torsion angles of the X-Ray structures of *cis-trans*-TWG1 and *cis-cis*-TWG1.

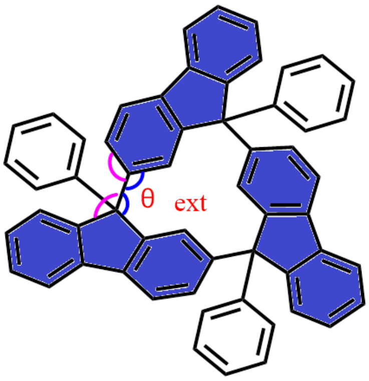

	θ_{ext}	average

***cis-trans*-TWG1<br>(single crystal)**	49.57°<br>67.11°<br>21.75°<br>127.52°<br>9.52°<br>95.38°	61.82°
***cis-trans*-TWG1<br>(co-crystal)**	17.49°<br>51.57°<br>70.22°<br>91.89°<br>132.47°<br>13.28°	62.82°
***cis-cis*-TWG1<br>(single crystal)**	65.42°<br>3.87°<br>57.54°<br>6.98°<br>64.24°<br>0.72°	33.13°
***cis-cis*-TWG1<br>(co-crystal)**	55.49°<br>63.77°<br>65.05°<br>8.67°<br>1.59°<br>0.92°	32.58

Section 5. The Calculation of strain energies of DWGs, TWGs, SWGs-F and TWGs-Th

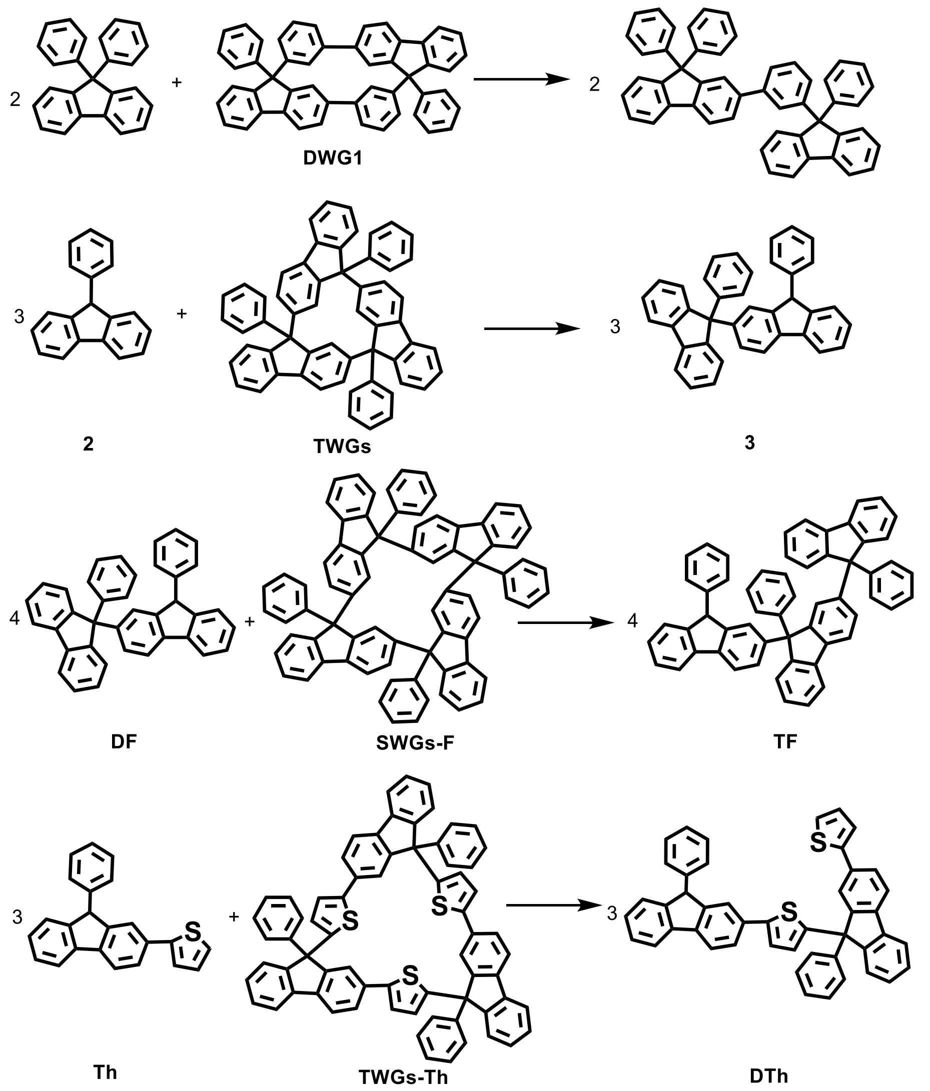

Supplementary Fig. 22 | Calculation formula of strain energies of DWGs, TWGs, SWGs-F and TWGs-Th.

Energy (**Ph**) = -963.8190078 a.u., Energy (***rac*-DWG1**) = -1925.238837 a.u., Energy (***meso*-DWG1**) = -1925.240847 a.u., Energy (**DPh**) = -1926.455465 a.u.,

Molecular strain energies (MSE, ***rac*-DWG1**) = 2*(energy of **Ph**)+(energy of ***rac*-DWG1**)-2*(energy of **DPh**) = 2*(-963.8190078)+(-1925.238837)-2*(-1926.455465）= 0.0340772 a.u.

MSE (*rac*-DWG1) =0.0340772*627.51 = 21.38378377 kcal/mol

MSE (***meso*-DWG1**) = 2*(energy of **Ph**)+(energy of ***meso*-DWG1**)-2*(energy of **DPh**) = 2*(-963.8190078)+(-1925.240847)-2*(-1926.455465）= 0.03206701 a.u.

MSE (*meso*-DWG1) = 0.03206701*627.51 = 20.12236945 kcal/mol

Energy (**2**) = -732.4727164 a.u., Energy (***cis-cis*-TWG1**) = -2193.7729125 a.u., Energy (***cis-trans*-TWG1**) = -2193.7684481 a.u., Energy (**3**) = -1463.7369963 a.u.

Molecular strain energies (MSE, ***cis-cis*-TWG1**) = 3*(energy of **2**)+(energy of ***cis-cis*-TWG1**)-3*(energy of **3**）= 3*(-732.4727164)+(-2193.7729125)-3*(-1463.7369963）

=0.019927 a.u.
MSE (***cis-cis*-TWG1**) =0.019927*627.51 = 12.49854 kcal/mol
MSE (***cis-trans*-TWG1**) = 3*(energy of **2**)+(energy of ***cis-trans*-TWG1**)-3*(energy of **3**) = 3*(-732.4727164)+(-2193.7684481)-3*(-1463.7369963) =0.024392 a.u.
MSE (*cis-trans*-TWG1) = 0.024392*627.51 = 15.29866 kcal/mol

Energy (**DF**) = -1463.7369963 a.u., Energy (**SWGs-F**) = -2925.0446609 a.u., Energy (**TF**) = -2194.9990723. a.u.
MSE (**SWGs-F**) = 4*(energy of **DF**)+(energy of **SWGs-F**)-4*(energy of **TF**) = =4*(-1463.7369963)+(-2925.0446609)-4*(-2194.9990723) = 0.0036431 a.u.
MSE (**SWGs-F**) = 0.0036431*627.51 = 2.284988751 kcal/mol

Energy (**Th**) = -1284.28601943 a.u., Energy (**SWGs-Th**) = -3849.22100722 a.u., Energy (**DTh**) = -2567.36377595 a.u.
MSE (**SWGs-Th**) = 3*(energy of **Th**)+(energy of **SWGs-Th**)-3*(energy of **DTh**) = 3*(-1284.28601943)+(-3849.22100722)-3*(-2567.36377595) = 0.01226234 a.u.
MSE (*cis-trans*-TWG1) = 0.01226234*627.51 = 7.691062273 kcal/mol

Section 6. Strain and aggregate effects of DWGs and TWGs.

Supplementary Table 19 | Summary of physical for all substrates and products.

λ: First absorption peak in solution (1*10^{-5}M in THF). $\lambda_{max,sol}$, PL: PL peaks in solutions (1*10^{-5}M in THF). $\lambda_{max,crystal}$, PL: PL peaks in film (5 mg/ml in THF). QY_{sol}: quantum yield in solution (1*10^{-5}M in THF). QY_{film}: quantum yield in film (5 mg/ml in THF).

sample	λ	$\lambda_{max,sol}$, PL	$\lambda_{max,film}$, PL	$\lambda_{max,crystal}$, PL	QY_{sol}	QY_{film}	$QY_{crystal}$	CIE_{film}	$CIE_{crystal}$
3h	317	334	358		86	8			
rac-DWG1	315	328	372	371	88	17	28	(0.16, 0.01)	(0.16, 0.01)
meso-DWG1	320	353	361	373	69	21	26	(0.17, 0.05)	(0.16, 0.09)
4a	313	328	335		83	9	-		
cis-cis-TWG1	323	358	393	373	26	25	8	(0.16, 0.02)	(0.16, 0.03)
cis-trans-TWG1	320	349	372	356	36	17	13	(0.16, 0.03)	(0.16, 0.05)

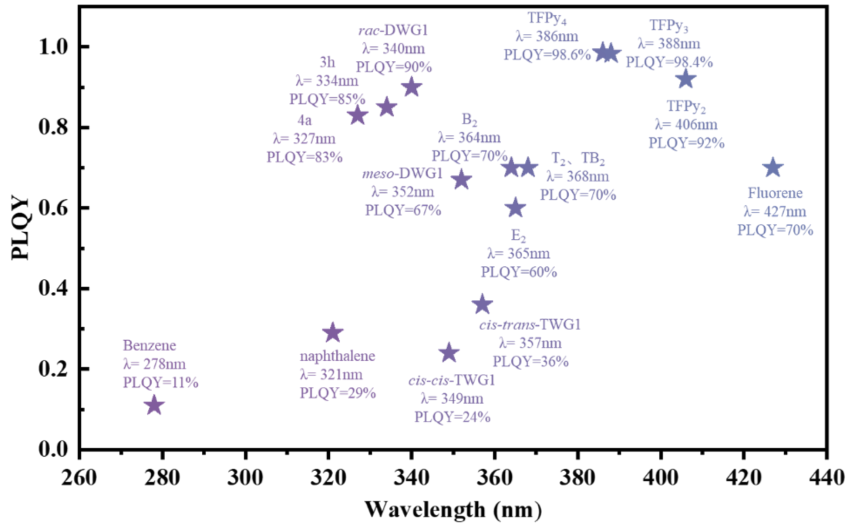

Supplementary Fig. 23 | Summary of physical for all substrates, products and other ultraviolet luminescent material. (Benzene, naphthalene and fluorene[1]; B_2, T_2, TB_2 and E_2[3]; $TFPy_2$, $TFPy_3$ and $TFPy_4$[5].)

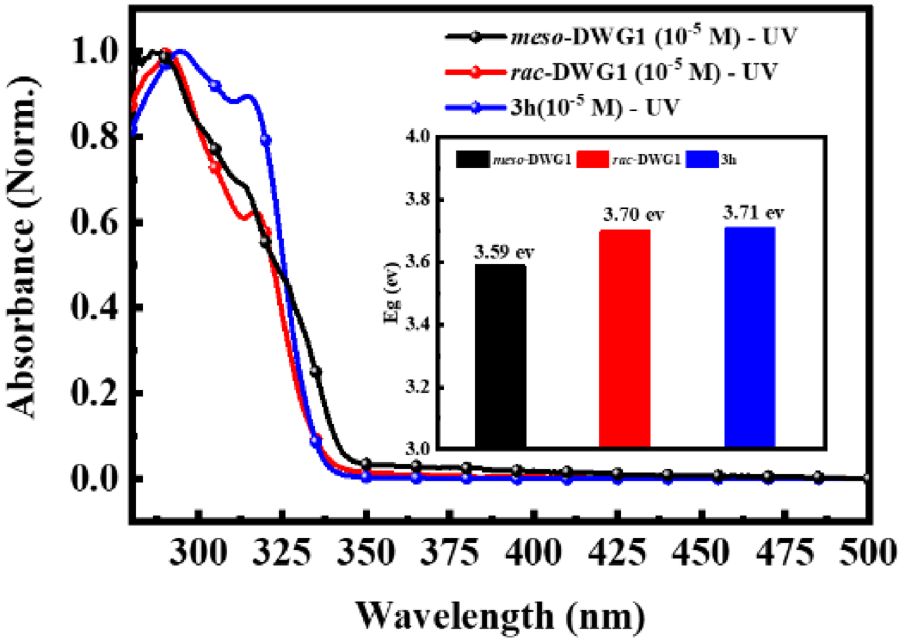

Supplementary Fig. 24 | UV-vis absorbance spectra of *meso*-DWG1, *rac*-DWG1 and 3h in THF solution.

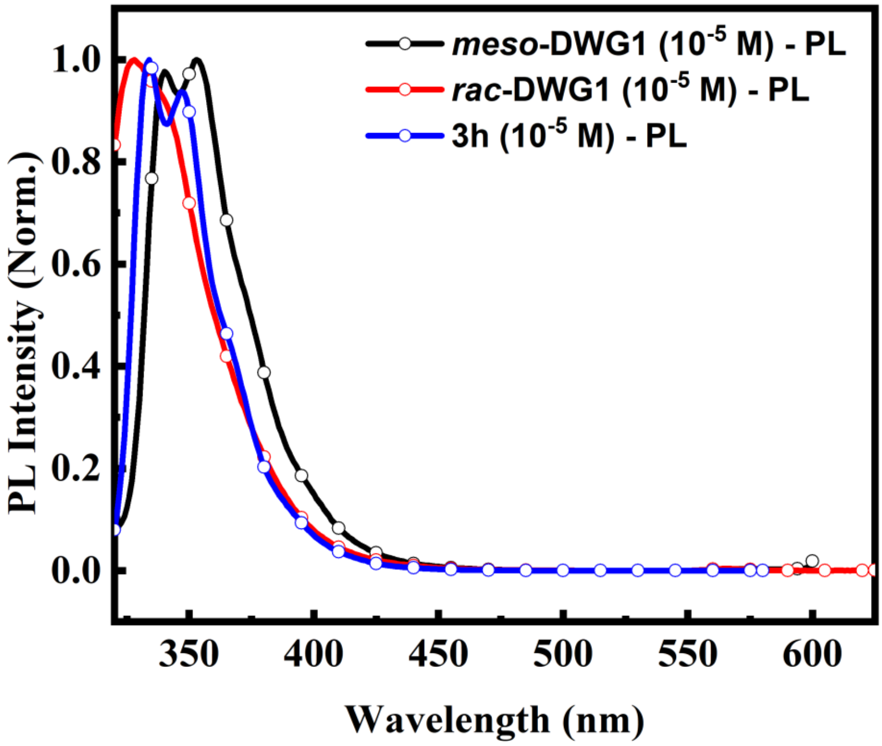

Supplementary Fig. 25 | PL spectra of *meso*-DWG1, *rac*-DWG1 and 3h in THF solution.

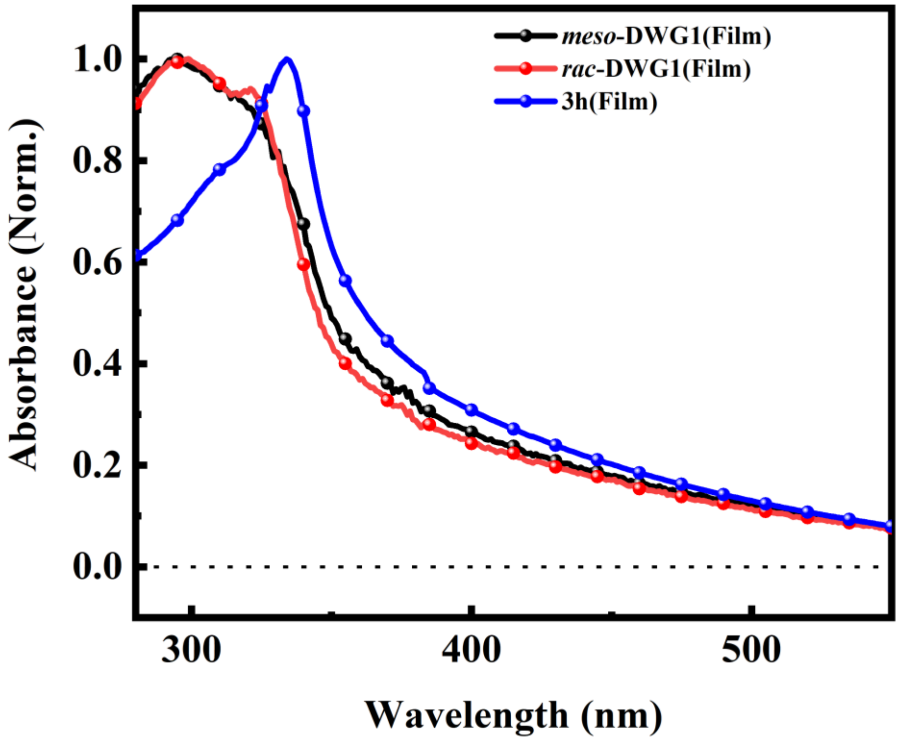

Supplementary Fig. 26 | UV-vis absorbance spectra of *meso*-DWG1, *rac*-DWG1

and 3h in film.

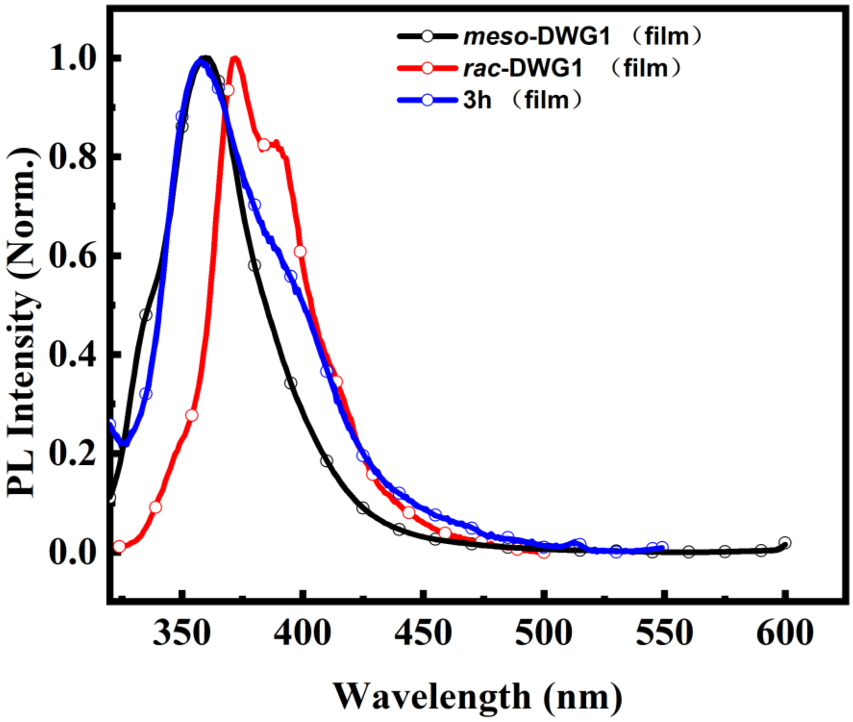

Supplementary Fig. 27 | PL spectra of *meso*-DWG1, *rac*-DWG1 and 3h in film.

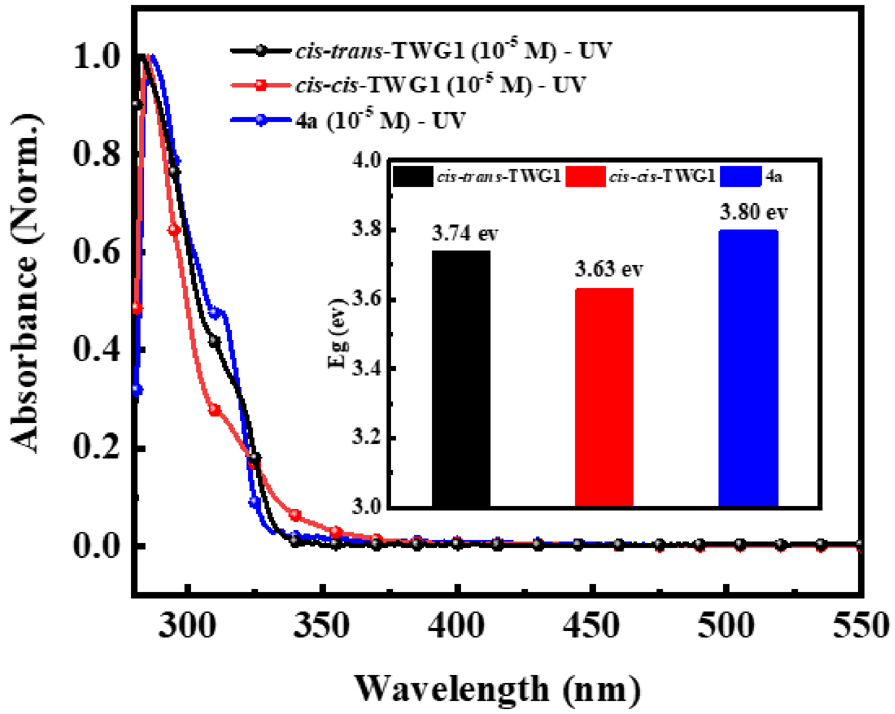

Supplementary Fig. 28 | UV-vis absorbance spectra of *cis-trans*-TWG1, *cis-cis*-TWG1 and 4a in THF solution.

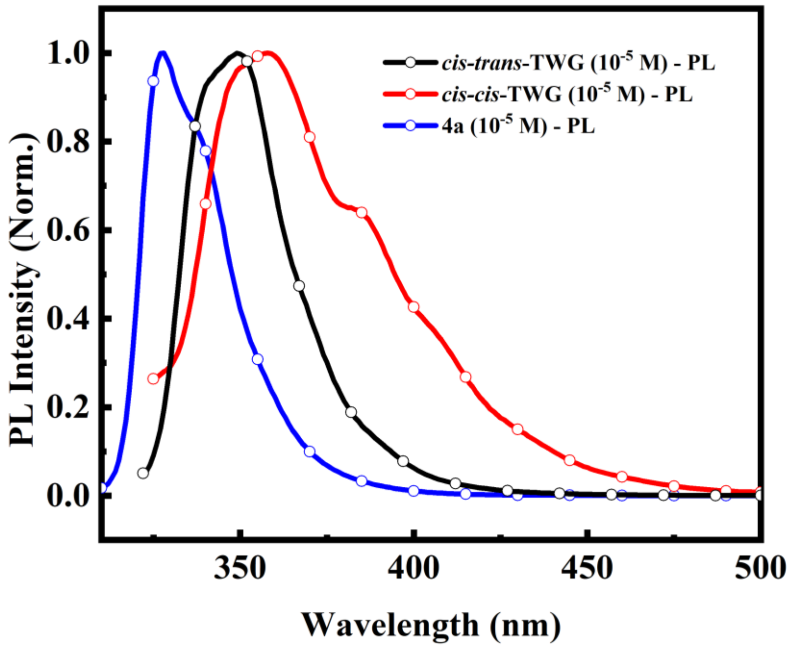

Supplementary Fig. 29 | PL spectra of of *cis-trans*-TWG1, *cis-cis*-TWG1 and 4a

in THF solution.

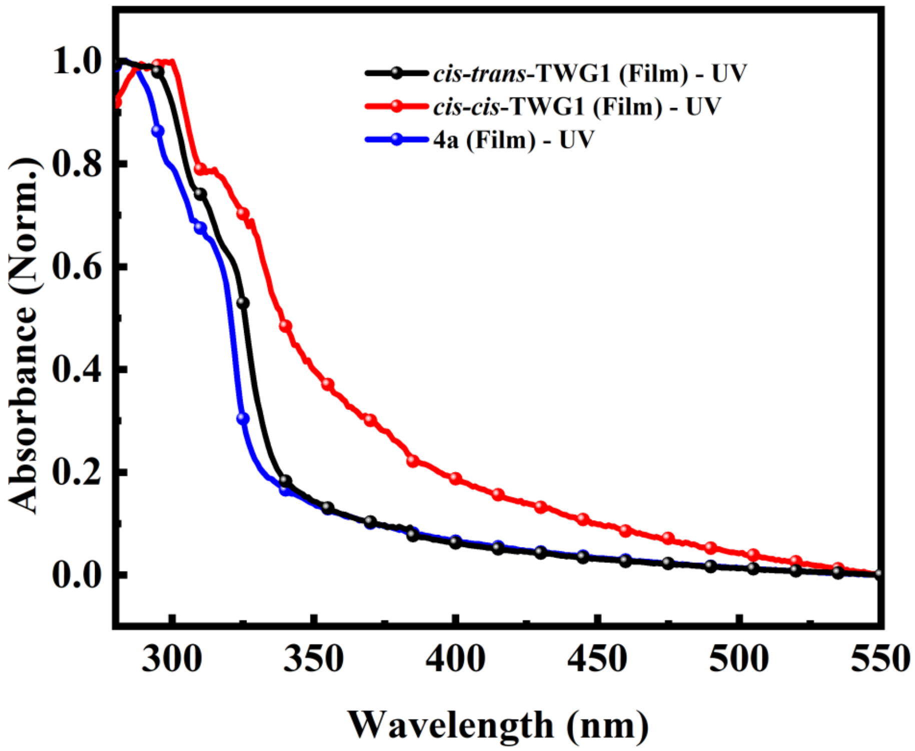

Supplementary Fig. 30 | UV-vis absorbance spectra of *cis-trans*-TWG1, *cis-cis*-TWG1 and 4a in film.

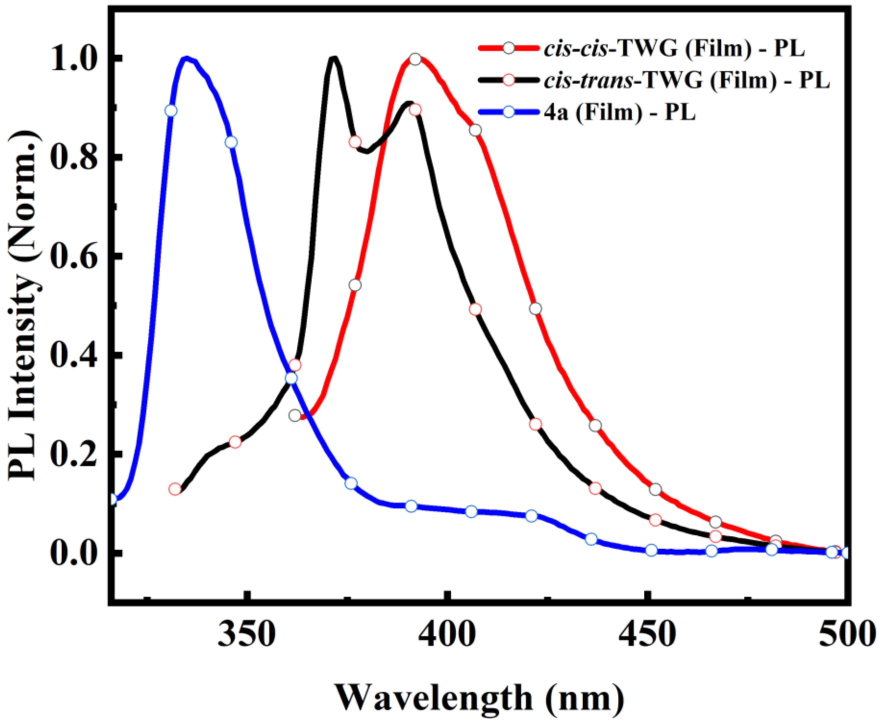

Supplementary Fig. 31 | PL spectra of *cis-trans*-TWG1, *cis-cis*-TWG1 and 4a in film.

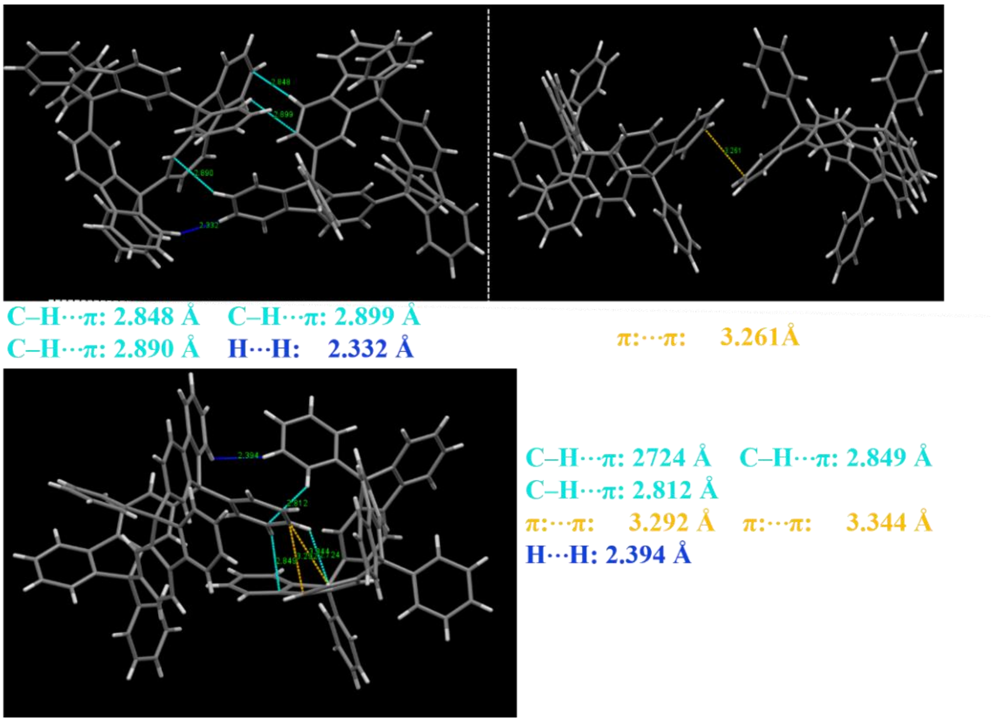

Supplementary Fig. 32 | The intermolecular interactions for *cis-trans*-TWG1. In the intermolecular interactions of *cis-trans*-TWG1, there are several types of interactions, including C–H··π interactions (2.724 Å, 2.812 Å, 2.848 Å, 2.849 Å, 2.890 Å and 2.899 Å), π··π interactions (3.261 Å, 3.292 Å and 3.344 Å) and H··H (2.332 Å and 2.394 Å) interactions within repeating units of octamers. C–H··π interactions predominantly occur between phenyl and fluorene (2.899 Å, 2.724 Å, 2.849 Å, 2.812 Å) and the vertexes between adjacent fluorenes (2.848 Å, 2.890 Å). Additionally, π··π stacking is observed at the vertexes between adjacent fluorenes (3.261 Å) and between fluorene and phenyl (292 Å, 3.344 Å). One H··H interaction is identified between H(1) and H(3) of a fluorene unit (2.332 Å), and another H··H interaction occurs between fluorene and phenyl with a distance of 2.394 Å.

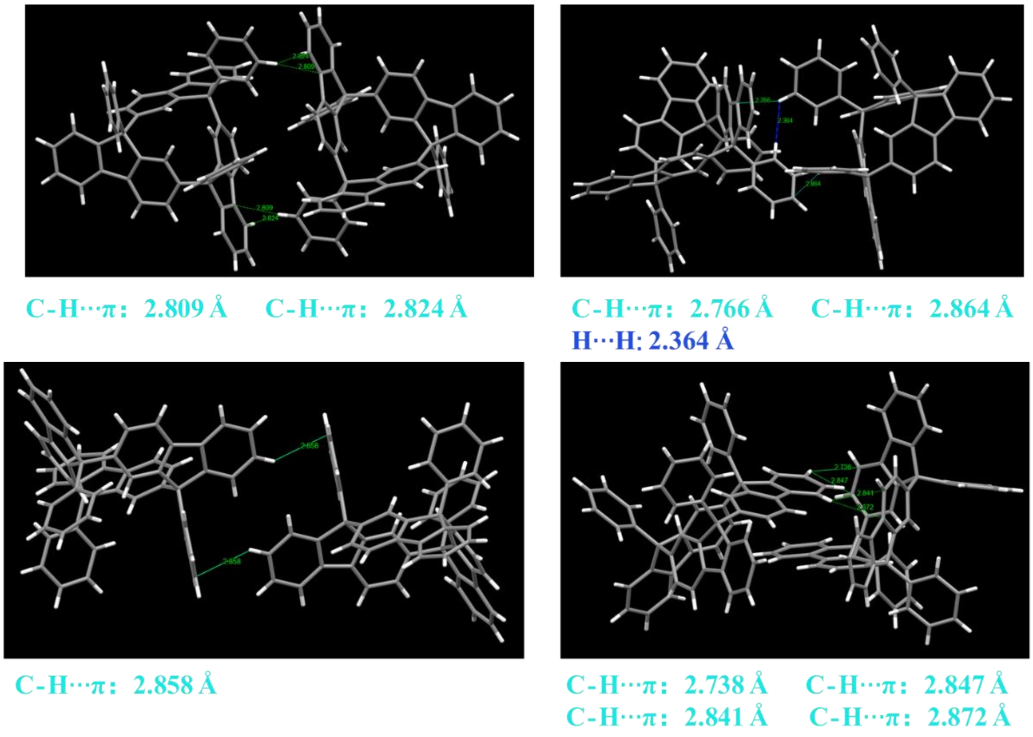

Supplementary Fig. 33 | The intermolecular interactions for *cis-cis*-TWG1. In the intermolecular interactions for *cis-cis*-TWG1, various interactions are observed, including C–H···π interactions (2.809 Å, 2.824 Å, 2.766 Å, 2.864 Å, 2.858 Å, 2.738 Å, 2.847 Å, 2.841 Å, 2.872 Å) and H···H interaction (2.364 Å). C–H···π interactions are predominantly present between phenyl and fluorene (2.808Å, 2.824 Å, 2.766 Å, 2.864 Å, 2.858 Å) and at the vertexes between adjacent fluorenes (2.738Å, 2.847 Å, 2.841 Å, 2.872 Å). The H···H interaction is specifically located within the phenyl unit with a distance of 2.364 Å.

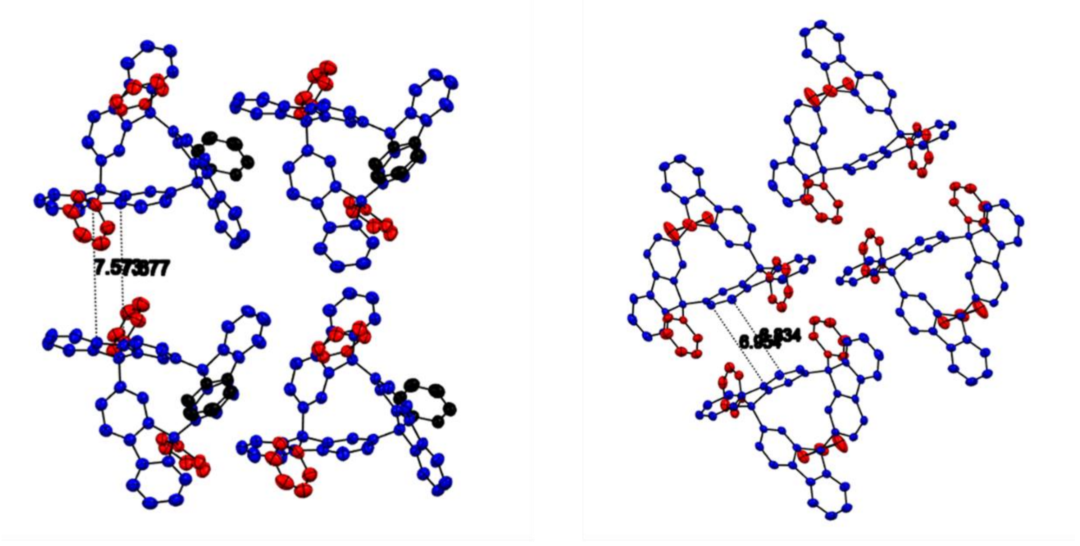

***cis-trans*-TWG1** ***cis-cis*-TWG1**

Supplementary Fig. 34 | The distances between the overlapping fluorene units of

TWGs. These distance are7.5~7.7 Å in *cis-trans*-TWG1, and 6.6~7.0 Å in *cis-cis*-TWG1.

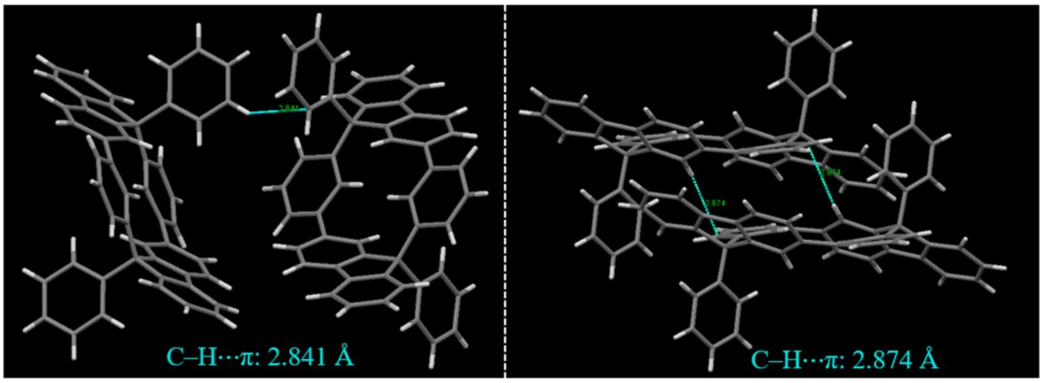

Supplementary Fig. 35 | The intermolecular interactions for *meso*-DWG1. In the intermolecular interactions of *meso*-DWG, a specific type of C–H···π interaction is observed, with distances of 2.841 Å and 2.874 Å. These interactions are primarily between the branched phenyl and phenyl (2.841 Å) and at the vertexes between adjacent fluorenes and phenyl (2.847 Å).

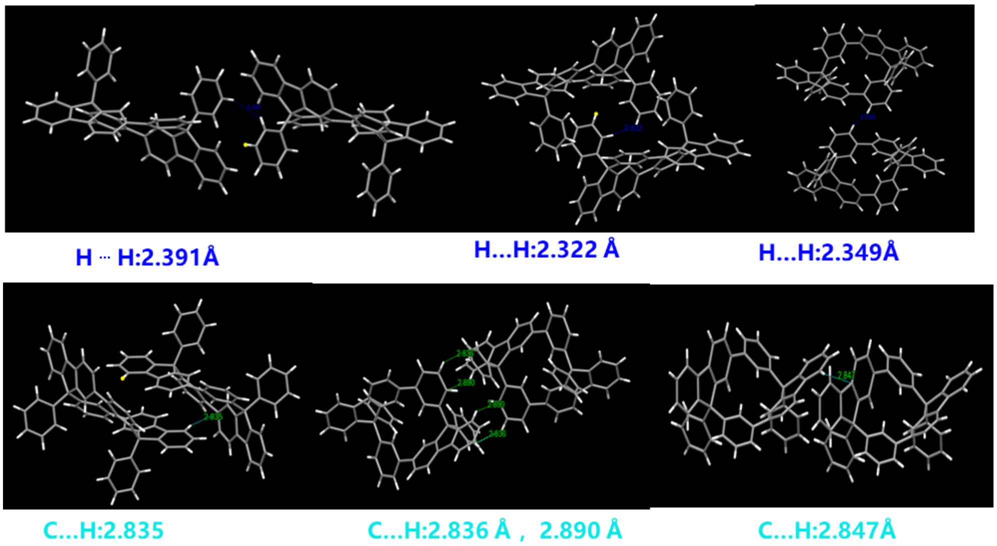

Supplementary Fig. 36 | The intermolecular interactions for *rac*-DWG1. In the intermolecular interactions of *rac*-DWG1, various interactions are identified, including C–H···π interactions (2.835Å, 2.836 Å, 2.890 Å, 2.847 Å) and H···H interaction (2.391 Å, 2.322 Å, 2.394 Å). C–H···π interactions predominantly occur between fluorene and fluorene (2.835Å, 2.847 Å), branched phenyl and backbone phenyl (2.836 Å), and between backbone phenyl and fluorenes (2.349 Å). H···H interaction are present in branched phenyl and phenyl (2.391 Å, 2.322 Å) and between backbone phenyl and phenyl (2.349 Å).

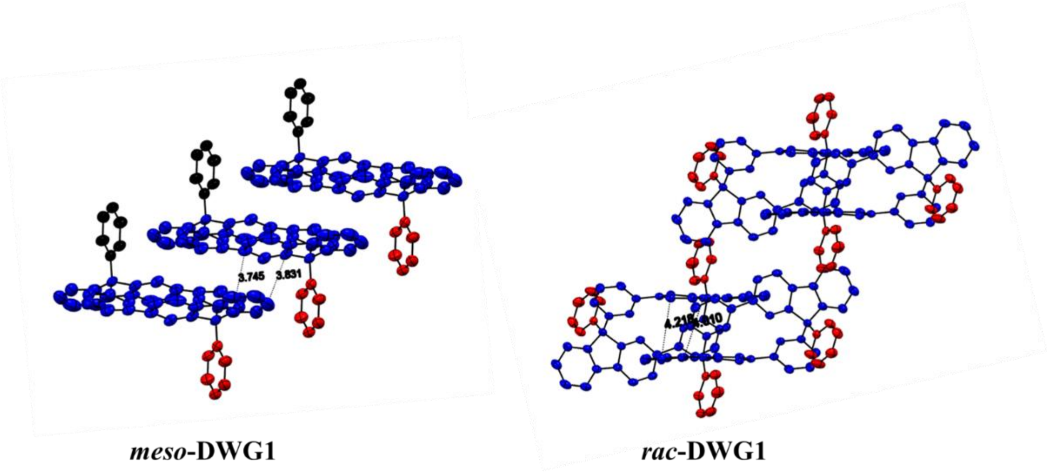

Supplementary Fig. 37 | The distances between the overlapping fluorene units of DWGs. these distance are 3.7~3.8 Å in *meso*-DWG1, 4.0~4.2Å in *rac*-DWG1.

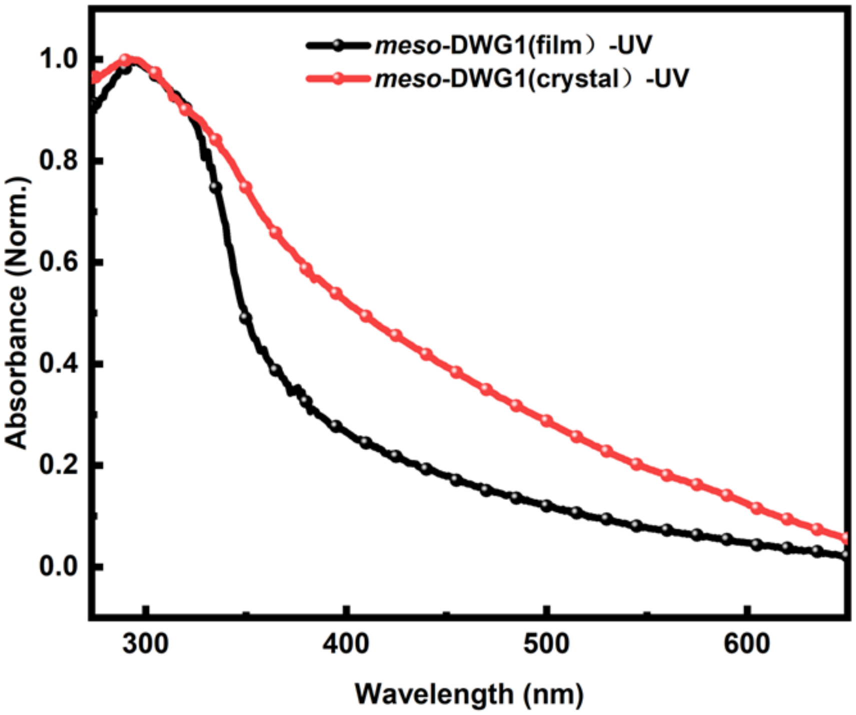

Supplementary Fig. 38 | UV-vis absorbance spectra of *meso*-DWG1 in single crystal and film.

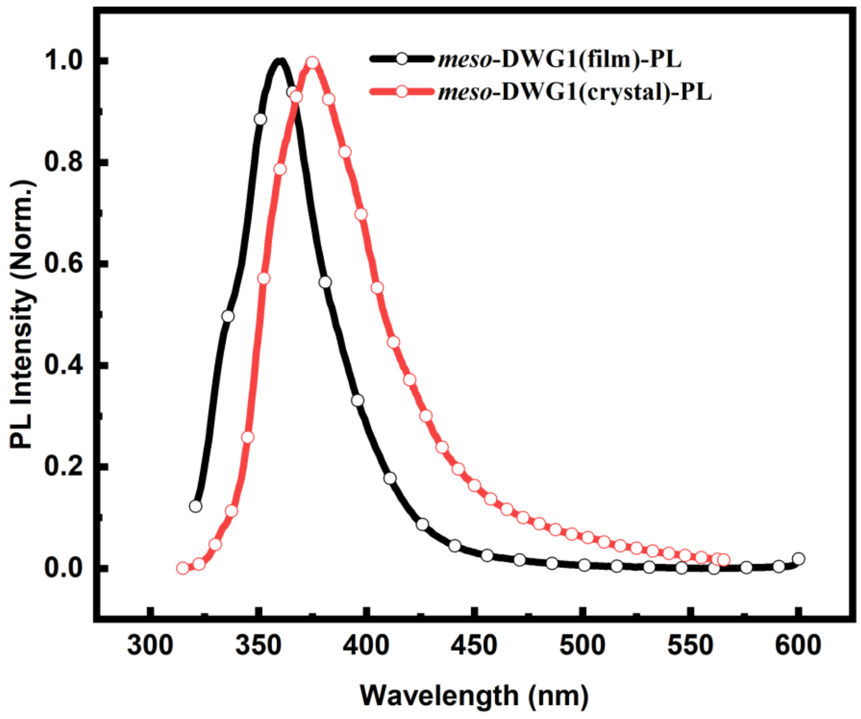

Supplementary Fig. 39 ｜PL spectra of *meso*-DWG1 in single crystal and film.

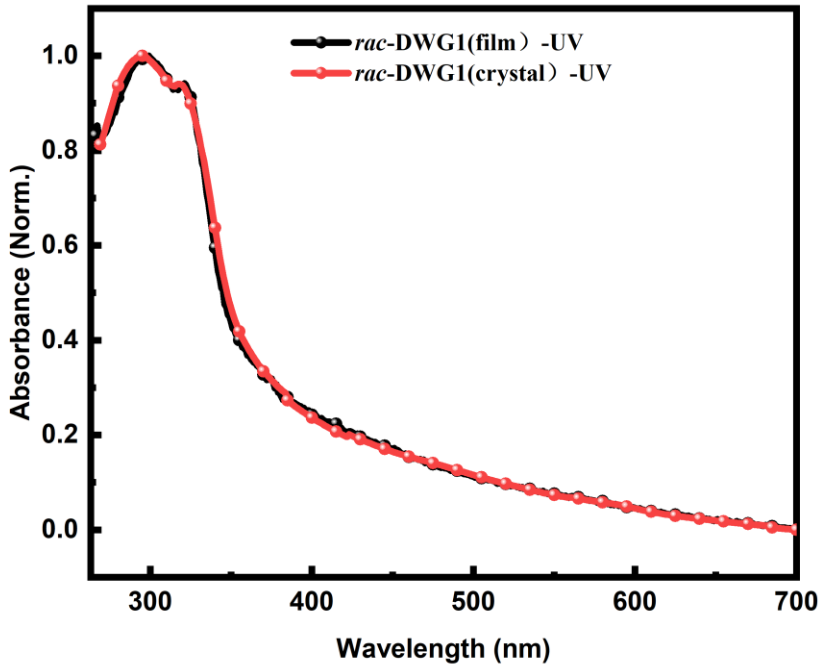

Supplementary Fig. 40 ｜UV-vis absorbance spectra of *rac*-DWG1 in single crystal and film.

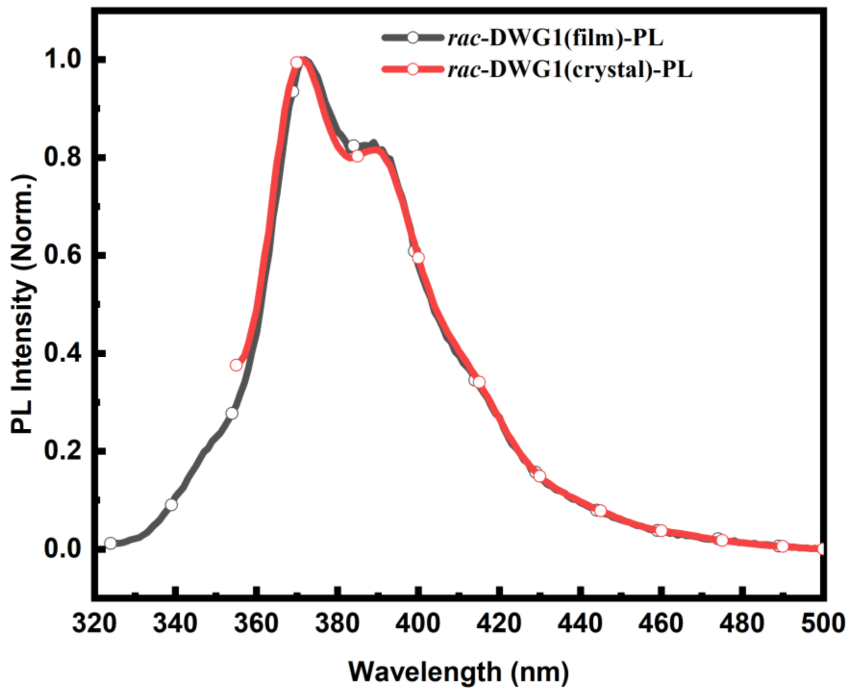

Supplementary Fig. 41 | PL spectra of *rac*-DWG1 in single crystal and film.

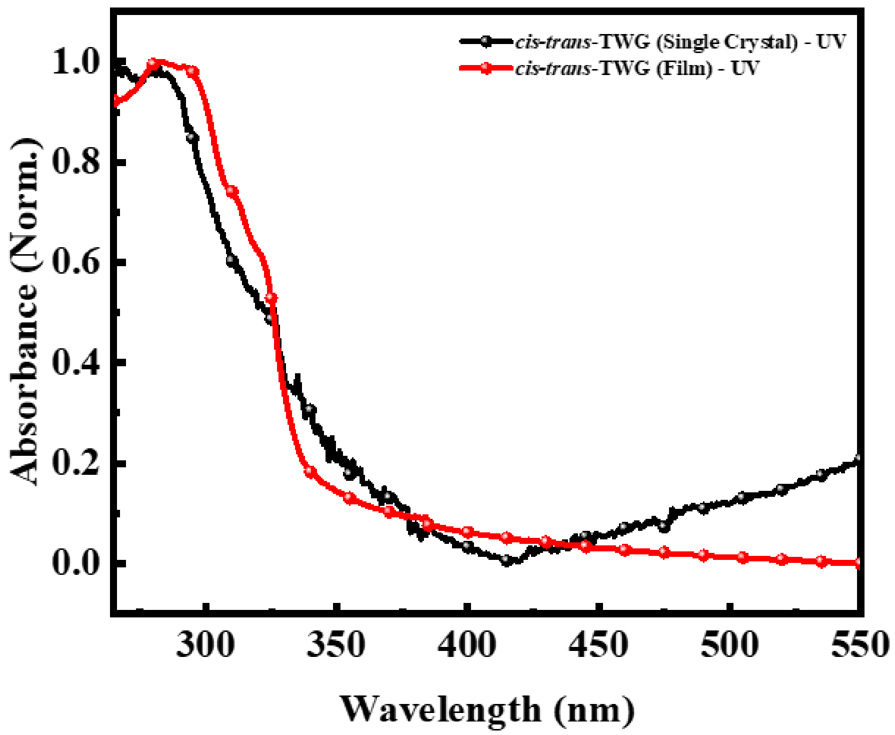

Supplementary Fig. 42 | UV-vis absorbance spectra of *cis-trans*-TWG1 in single crystal and film.

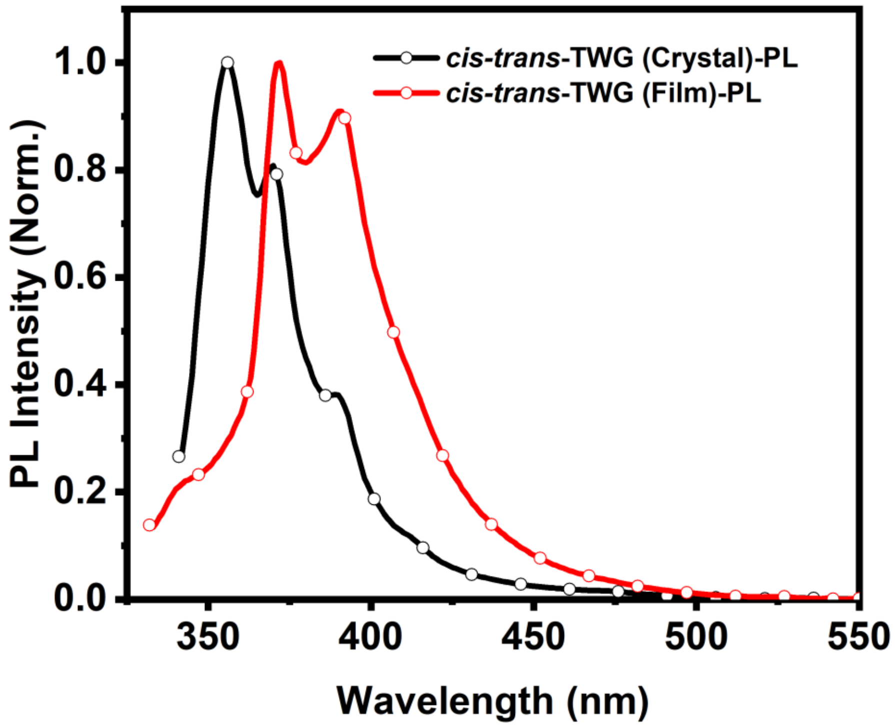

Supplementary Fig. 43 ｜PL spectra of *cis-trans*-TWG1 in single crystal and film.

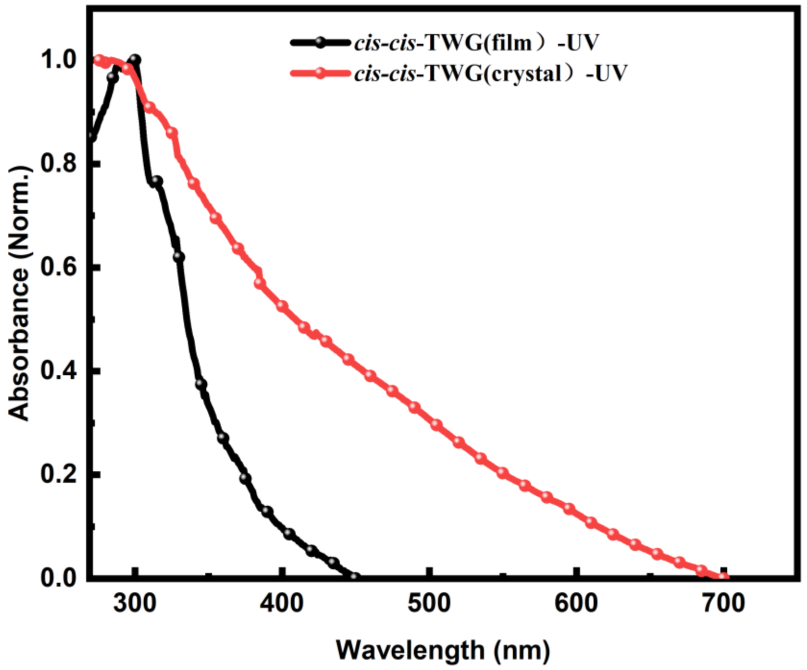

Supplementary Fig. 44 ｜UV-vis absorbance spectra of *cis-cis*-TWG1 in single crystal and film.

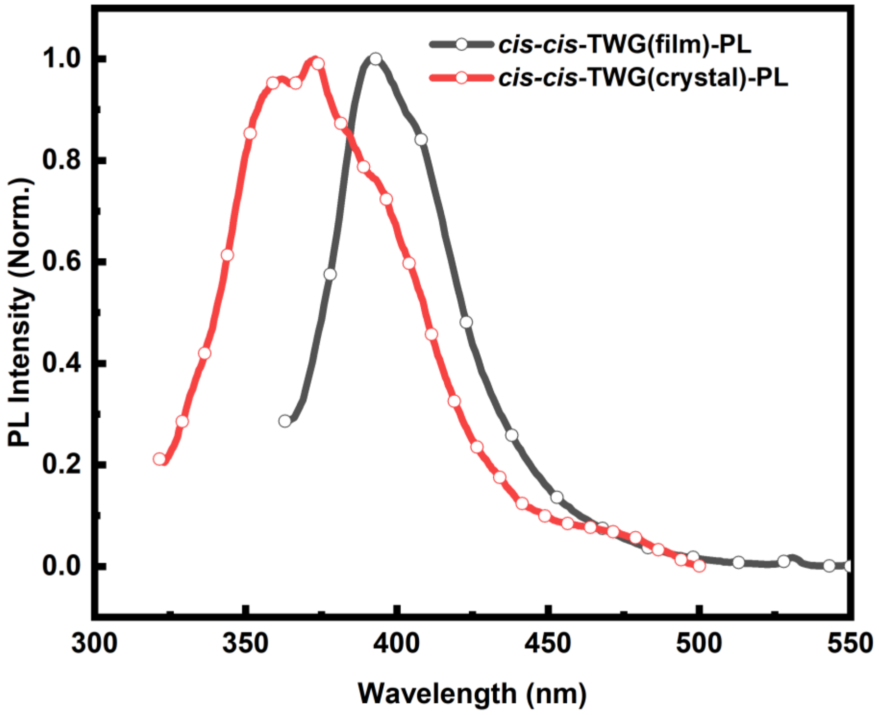

Supplementary Fig. 45 | PL spectra of *cis-cis*-TWG1 in single crystal and film.

Section 7. chiroptical properties and UV OLED

Supplementary Table 20 | Separation method of *cis-trans*-TWGs in HPLC.

Column	:	CHIRALPAK IJ
Column size	:	5.0 cm I.D. × 25 cm L, 10µm
Mobile phase	:	MeOH/DCM=80/20(V/V)
Flow rate	:	30 ml/min
Wave length	:	UV 254 nm
Temperature	:	38 °C

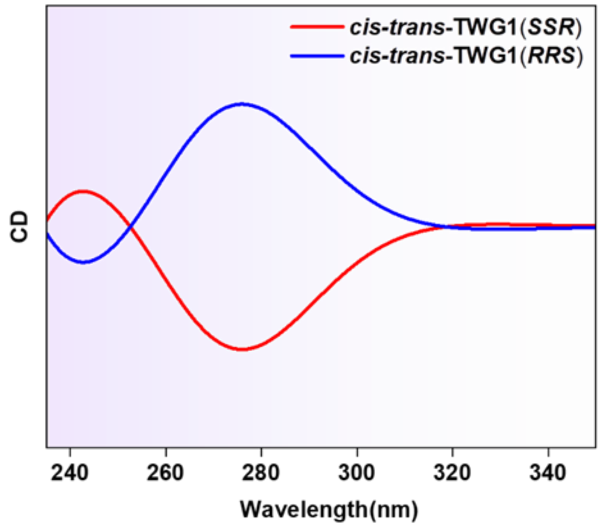

Supplementary Fig. 46 | Simulated circular dichroism spectra of *cis-trans*-TWG1(*SSR*) and *cis-trans*-TWG1(*RRS*) by TD- All the quantum chemical **at the CAM-B3LYP/TZVP level.**

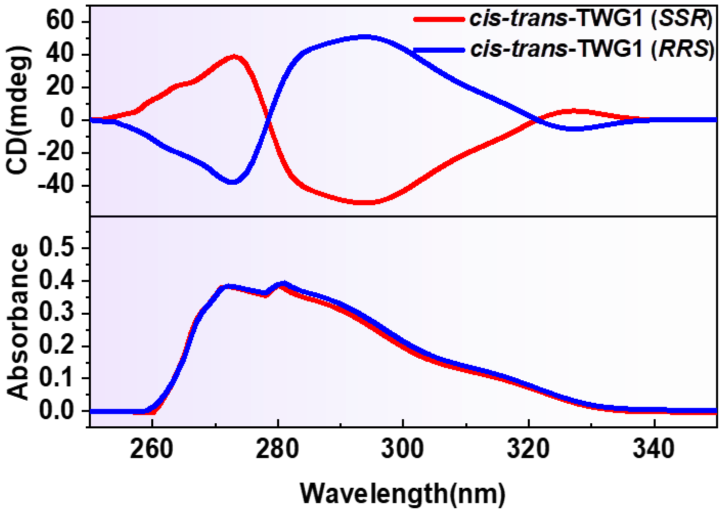

Supplementary Fig. 47 | The UV-vis absorption and CD spectra of *cis-trans*-TWG1 (*SSR*) and *cis-trans*-TWG1 (*RRS*) in toluene solution ($C = 5\times10^{-6}$ M).

Supplementary Table 21 | EL performances of device1 and device2 based on *cis-trans*-TWG1 as emitter.

	λ_{EL} (nm)	V_{on} (V)	L (cd/m^2)	η_C (cd/A)	η_P (lm/W)	EQEmax(%)	CIE (x, y)
device1	370	4.4	189	0.23	0.16	2.32	0.166,0.047
device2	386	4.4	248	0.40	0.28	4.17	0.173,0.043

Supplementary Table 22 | Summary of representative performances of UV OLEDs (EL Peak ≤400 nm)

UV emitter	λ_{EL} (nm)	EQE_{max} (%)	FWHM (nm)	CIE (x, y)	Ref.
PBD	394	0.1			[6]
PBD	376	4.1			[7]
PBD	400	2.3	47		[8]
TAZ	376	4.4	34		[9]
TAZ	376	4.14	37		[10]
TAZ	377	4.6	35		[11]
TAZ	384	3.65			[12]
OXD-7	388	1.43			[13]
mCP:Purine 2	393	1.6			[14]
PS-4	357	0.012		0.36, 0.35	[15]
B2	392	3.6			[4]
TB2	396	2.7			[4]
1SBFN	385	2.9			[16]
2SBFN	382	2.2			[16]
BSBFB	388	1.6			[16]
TSBFB	395	2.1			[16]
CBP	390	1.25			[17]
DPPP	396	2.2	50	0.16, 0.041	[18]
SSS	393	2.6			[19]
CBP:3a	396	2.8	52	0.16, 0.05	[20]
Tol−PPI	366.4	1.58	15.2		[21]
2Na-CzCN (HLCT)	398	5.92	53	0.15,0.06	[22]
CBP:CTPPI (HLCT)	396	7.9	55	0.16, 0.04	[23]
PPF:C2MPI (HLCT)	396	3.3		0.179, 0.086	[24]
DCz-pPy (HLCT)	397	3.6	22		[25]
CzSi:2BuCz-CNCz (HLCT)	396	10.79	33	0.161, 0.031	[26]
PPF:POPCN-2CP (HLCT)	388	5.6	45	0.161, 0.028	[27]
PPF:ICZ-TAZ (TADF)	388	3.26	32	0.172, 0.069	[27]
CzSi:CZ-MPS (TADF)	389	9.3	67	0.175, 0.065	[28]
mCP:tBCzP2PO (TADF)	384	15.1	32	0.14, 0.04	[29]
cis-trans-TWG1 (Device 1)	370	2.32	34	0.166, 0.047	This work
cis-trans-TWG1 (Device 2)	386	4.17	49	0.173, 0.043	This work

In the last several decades, there is rare report to prepare organic molecules for the fabrication of the efficient ultraviolet OLEDs with peak wavelength below 400 nm, due to their high energy of ultraviolet photon and wide bandgap. Recently, multiple resonances planar organic molecules for TADF devices are attracted more attentions for efficient and narrowband doped ultraviolet OLEDs, associated with low vibration relaxation and molecular perturbation, stable molecular configuration between ground and excited states (*Nature Photonics* **13** (2019): 678-682. Angewandte Chemie International Edition, 2022, **61**(48): e202209425. Aggregate, 2022, **3**(2): e144. Angewandte Chemie International Edition, 2023, **62**(46): e202312666. Advanced Optical Materials, 2022, 10(22): 2201714.). Besides, TAZ type molecules is few ultraviolet fluorescent emitter for the efficient non-doped OLED (EQE > 3.6%, CIE: 0.17, 0.04) (Organic Electronics 2020, **82**, 105718), which can further improve to EQE > 4.14% need to introduce novel hole transport layers for the realization of the balance between hole and electron injection/transpoortation (*Journal of Materials Chemistry C* 2019, **7**, 926-936. *Applied Physical Letters* 2017, 110, 043301. *Organic Electronics* 2017, **46**, 7-13). In fact, it is reasonably predicated that the fragile C-N, C-P and C-S bonds in the organic conjugated molecules may result into their and OLEDs instability (*Angew. Chem.* 2022*,* **134***,* e202207204. *J. Appl. Phys.* 2007, **101**, 024512. *J. Phys. Chem. C* 2012, **116**, 19451–19457. *J. Phys. Chem. C* 2014, **118**, 7569–7578.), especial for the ultraviolet and blue OLEDs. Then, hydrocarbons aromatic emitters also had a intrinsically stability to resist high band exiton for stable ultraviolet and blue OLEDs, benefits of the removal of heteroatoms to avoid the weak and chemical bond in the chromophore, similar to the commercialized anthracene (*Nat Commun* 2023, **14**, 3927. *Angew. Chem.* 2022*,* **134***,* e202207204). To the best of our knowledge about SPPP reported narrowband non-doped ultraviolet OLEDp (CIE_y < 0.04), the great majority showed the EQE lower than 4%. In addition, compared to the planar and fused aromatic emitters, non-planar conjugated molecules with a multi-dimensional topical structure showed an extremely weak intermolecular aggregation and interaction to obtain single-molecular emission behavior with low ratio of non-radiative transitions (*NPG Asia Materials*, 2021, **13**(1): 53.). Therefore, it is very excited to obtain a narrowband and efficient ultraviolet non-doped OLEDs based on the novel steric hydrocarbon TWG. Therefore, it is reasonably believed that our novel steric nanohydrocarbons have a promising application in the efficient and stable ultraviolet OLEDs.

Section 7. GC-MS, MALDI-TOF-MS and NMR spectra for all pbstrates and products.

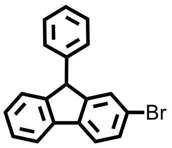

1a

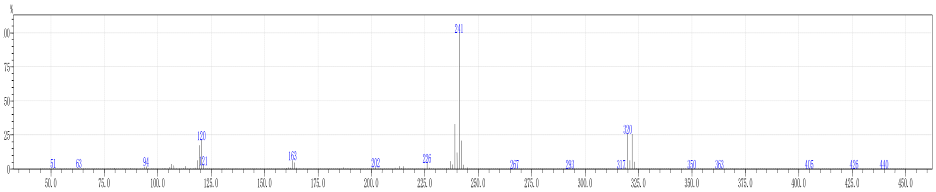

Supplementary Figure. 48 | GC-MS spectrum of 1a.

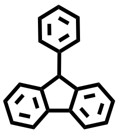

2a

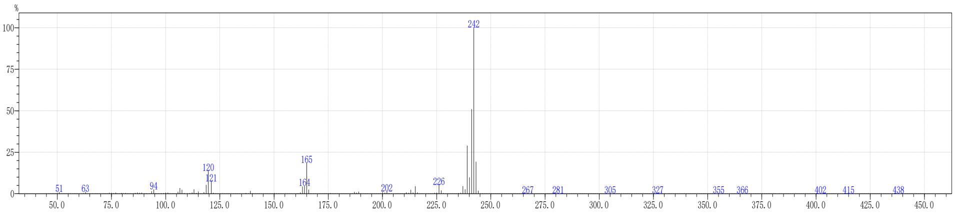

Supplementary Figure. 49 | GC-MS spectrum of 9-phenyl-fluorene.

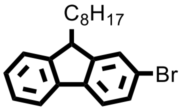

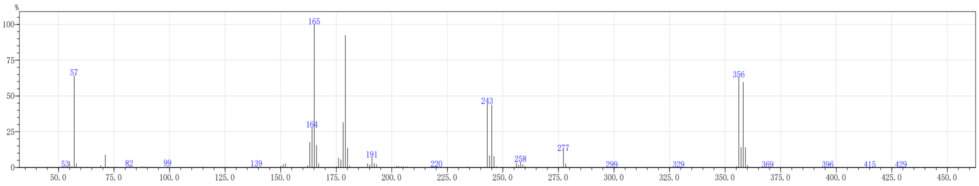

Supplementary Supplementary Fig. 50 | GC-MS spectrum of 2-bromo-9-octylfluorene**.**

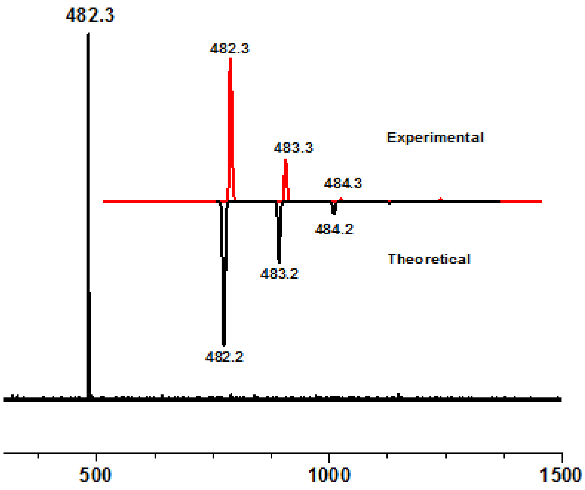

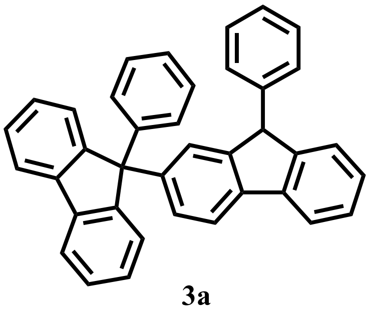

3a

Supplementary Supplementary Fig. 51 | MALDI-TOF-MS spectrum of 9-9'-diphenyl-9*H*,9'*H*-2,9'-bifluorene.

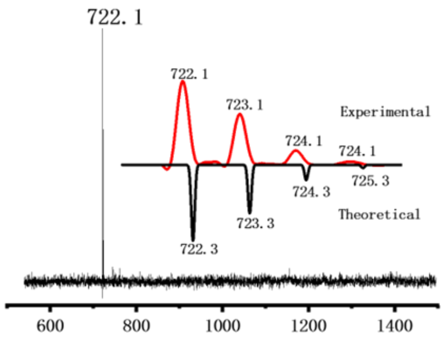

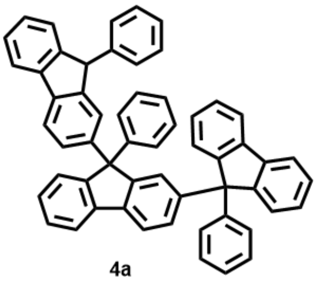

4a

Supplementary Supplementary Fig. 52 | MALDI-TOF spectrum of trimerized product (4a).

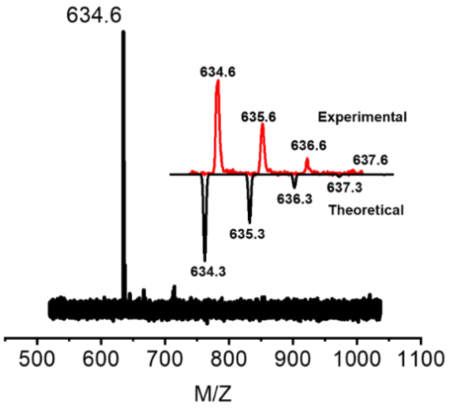

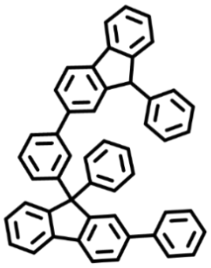

Supplementary Supplementary Fig. 53 | MALDI-TOF spectrum of trimerized product (3h).

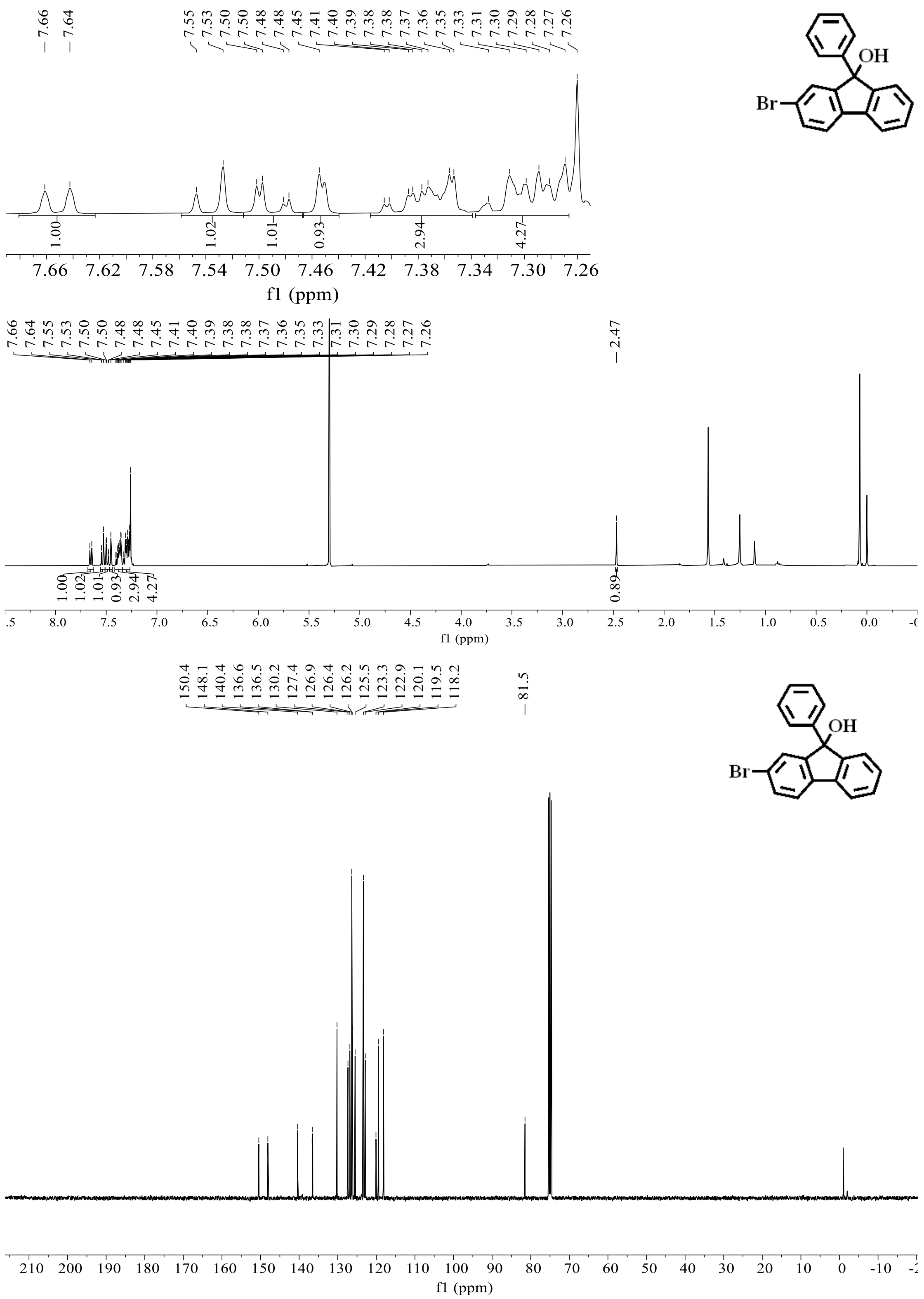

Supplementary Figure. 54 | ^{1}H and ^{13}C-NMR Spectra of BrFOH. The bruker 400 MHz Fourier Transform NMR spectrometer was used to obtain ^{1}H and ^{13}C NMR spectra at a frequency of 400 MHz and 100 MHz in $CDCl_3$ at 20 °C, respectively.

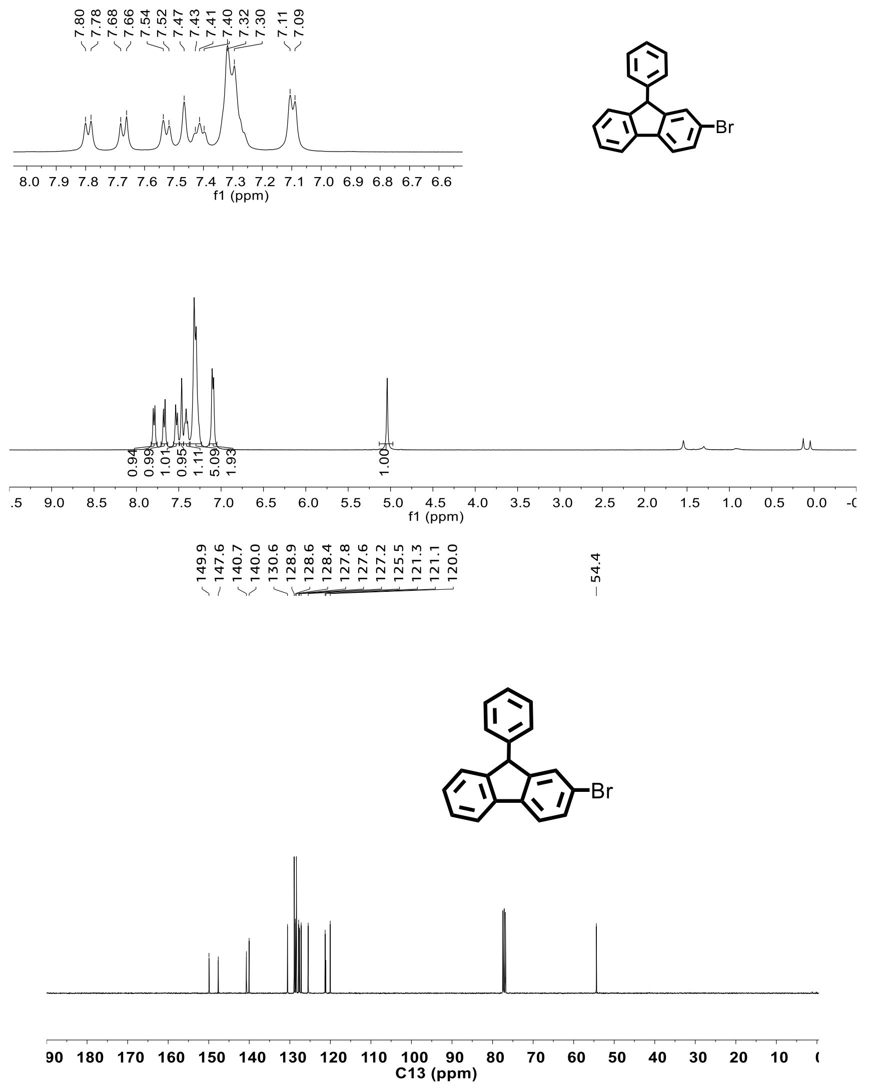

Supplementary Figure. 55 | ^{1}H and ^{13}C-NMR Spectra of 1a. The bruker 400 MHz Fourier Transform NMR spectrometer was used to obtain ^{1}H and ^{13}C NMR spectra at a frequency of 400 MHz and 100 MHz in $CDCl_3$ at 20 ^{o}C, respectively.

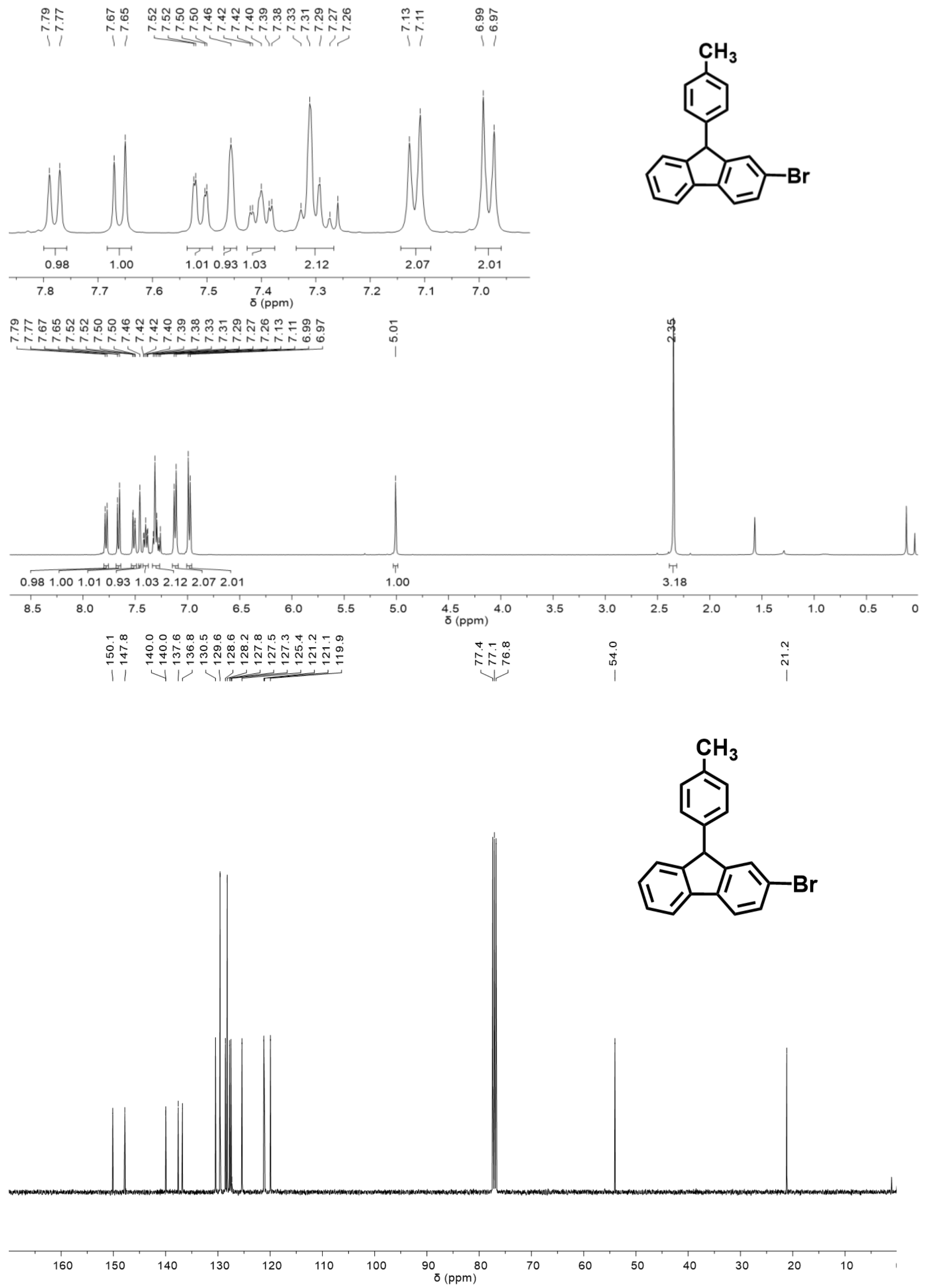

Supplementary Figure. 56 ｜ ^{1}H and ^{13}C-NMR Spectra of 1b. The bruker 400 MHz Fourier Transform NMR spectrometer was used to obtain ^{1}H and ^{13}C NMR spectra at a frequency of 400 MHz and 100 MHz in $CDCl_3$ at 20 ^{o}C, respectively.

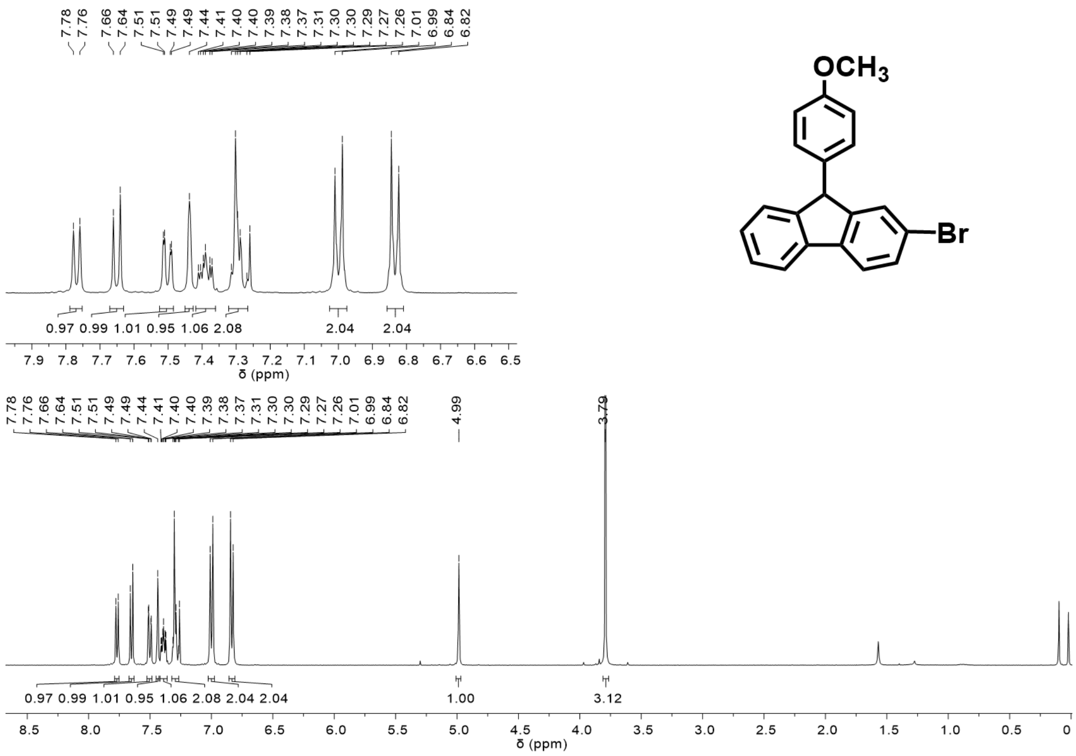

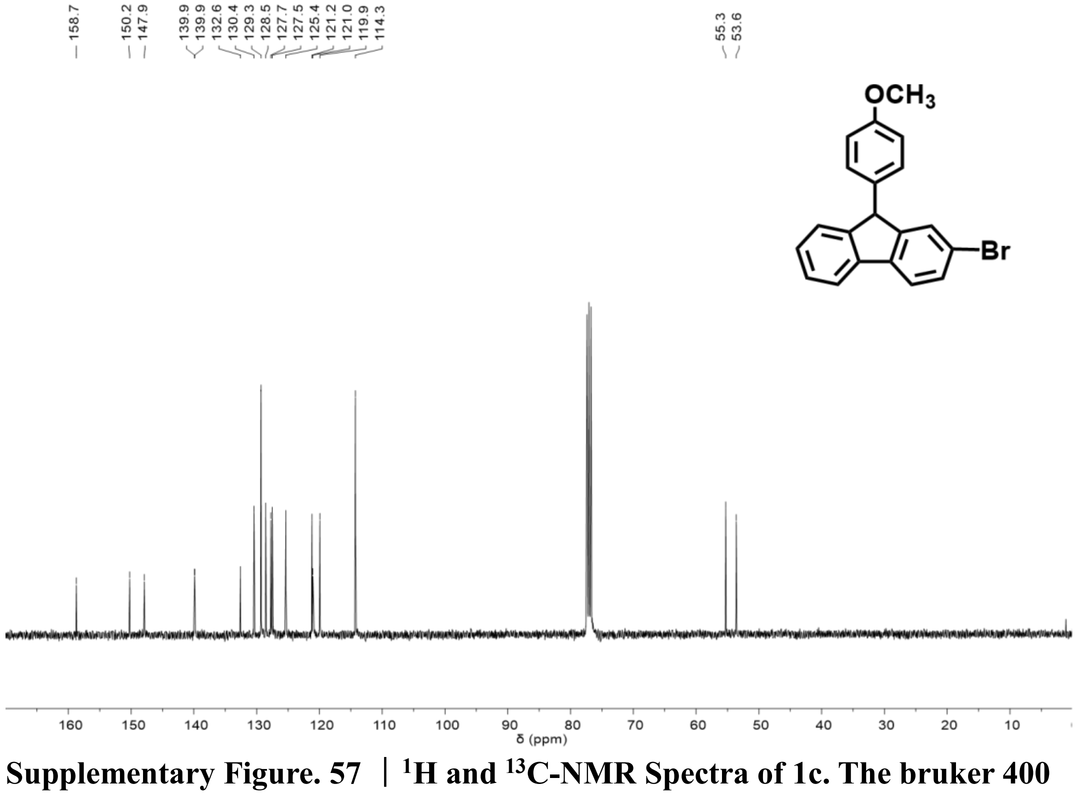

Supplementary Figure. 57 ｜ ^{1}H and ^{13}C-NMR Spectra of 1c. The bruker 400 MHz Fourier Transform NMR spectrometer was used to obtain ^{1}H and ^{13}C NMR spectra at a frequency of 400 MHz and 100 MHz in $CDCl_3$ at 20 ^{o}C, respectively.

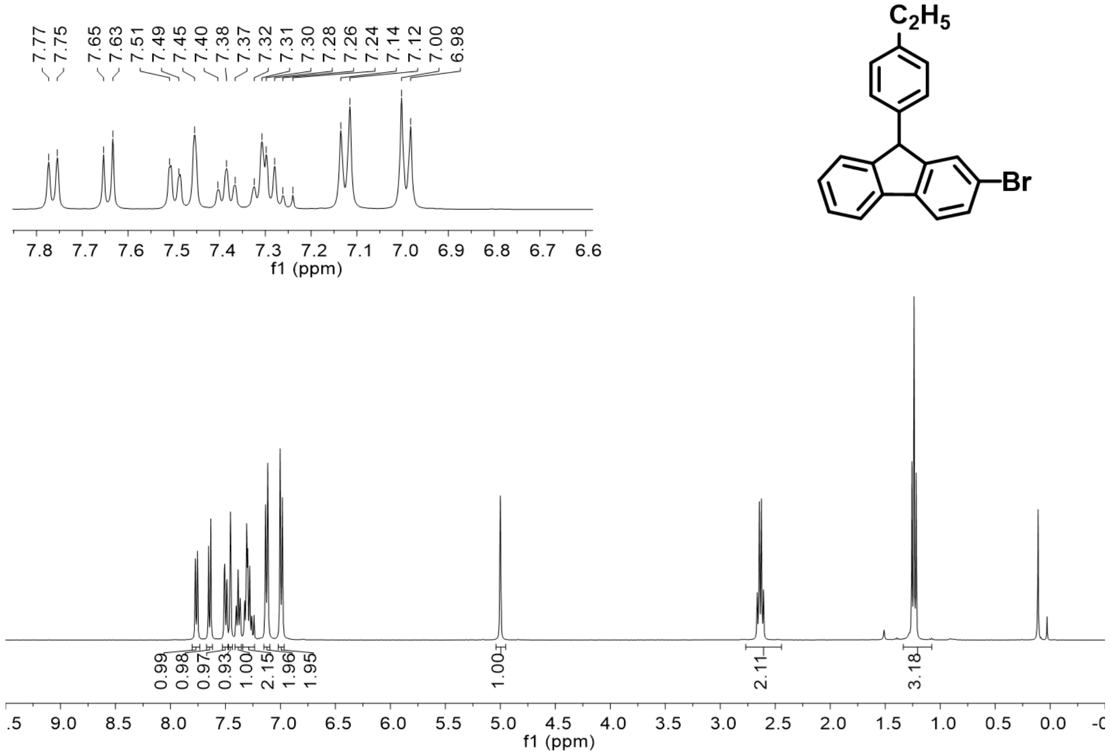

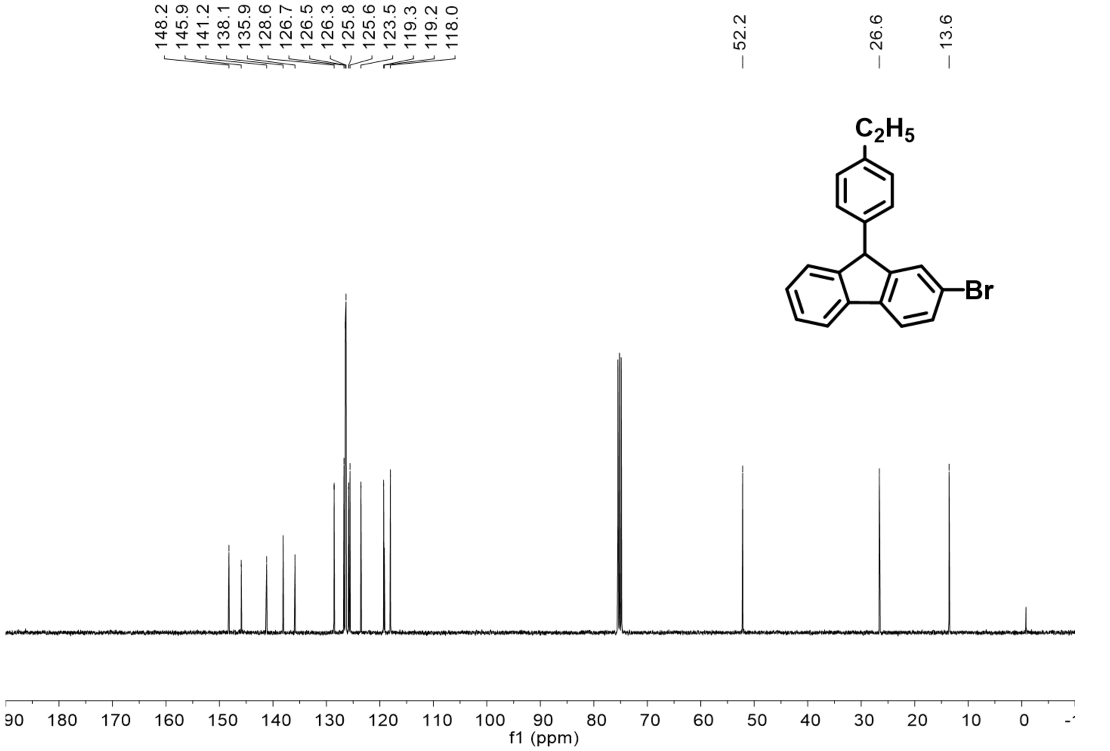

Supplementary Figure. 58 | ^{1}H and ^{13}C-NMR Spectra of 1d. The bruker 400 MHz Fourier Transform NMR spectrometer was used to obtain ^{1}H and ^{13}C NMR spectra at a frequency of 400 MHz and 100 MHz in $CDCl_3$ at 20 ^oC, respectively.

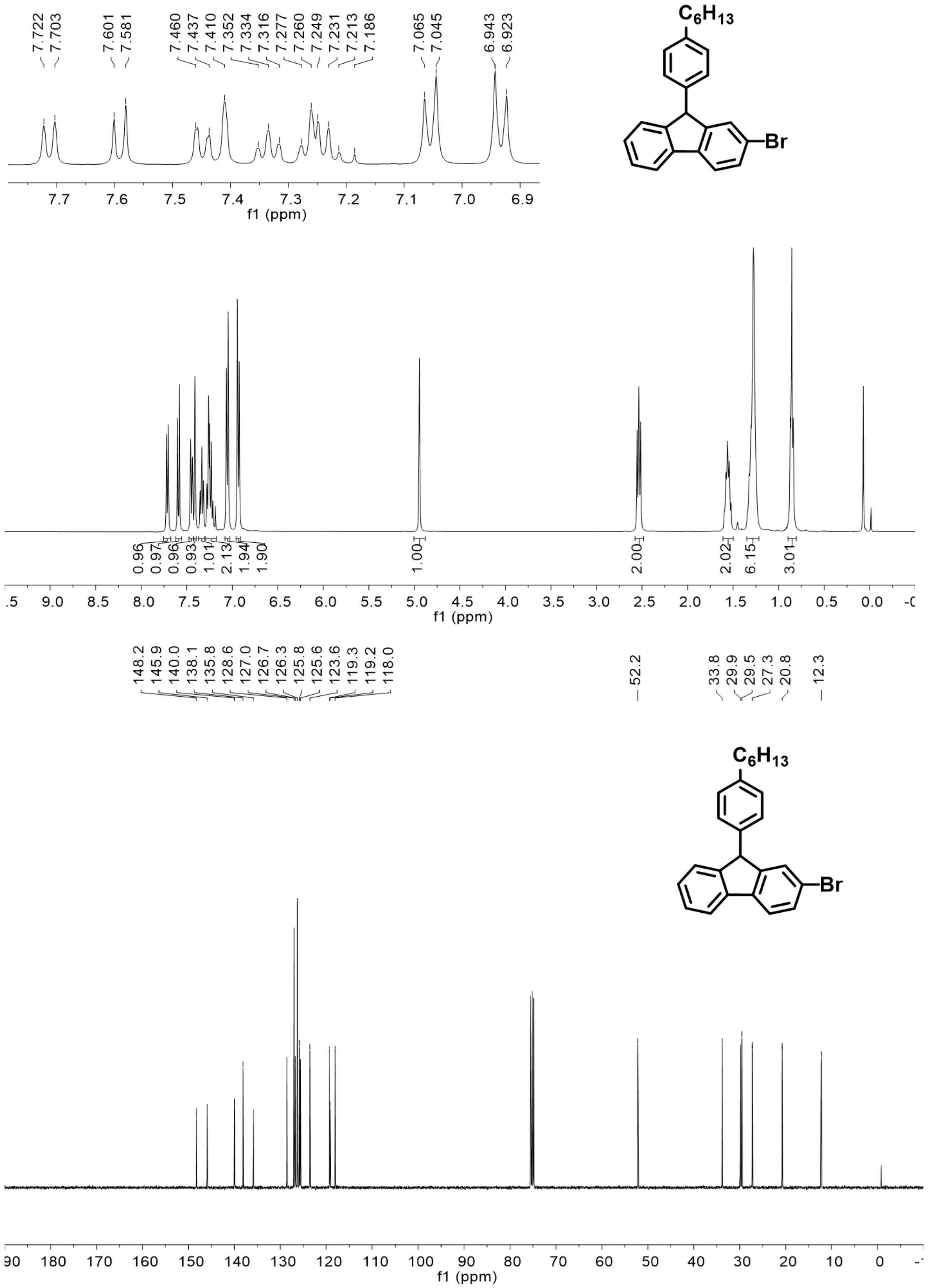

Supplementary Figure. 59 ｜ ^{1}H and ^{13}C-NMR Spectra of 1e. The bruker 400 MHz Fourier Transform NMR spectrometer was used to obtain ^{1}H and ^{13}C NMR spectra at a frequency of 400 MHz and 100 MHz in $CDCl_3$ at 20 ^oC, respectively.

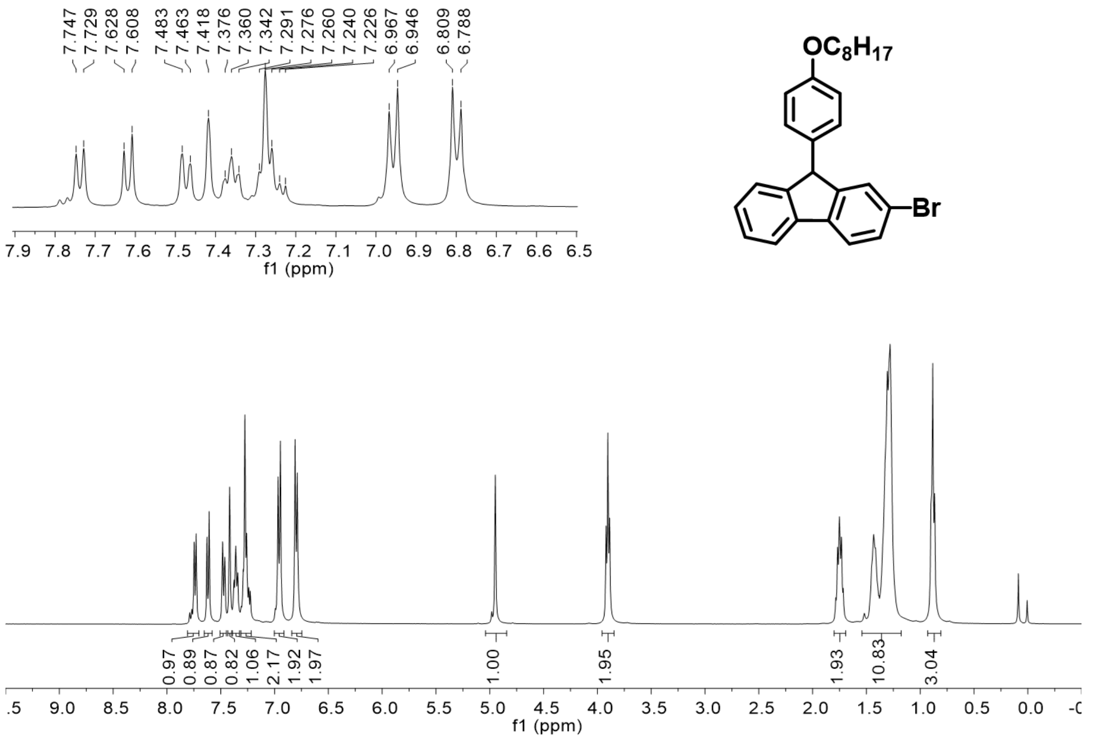

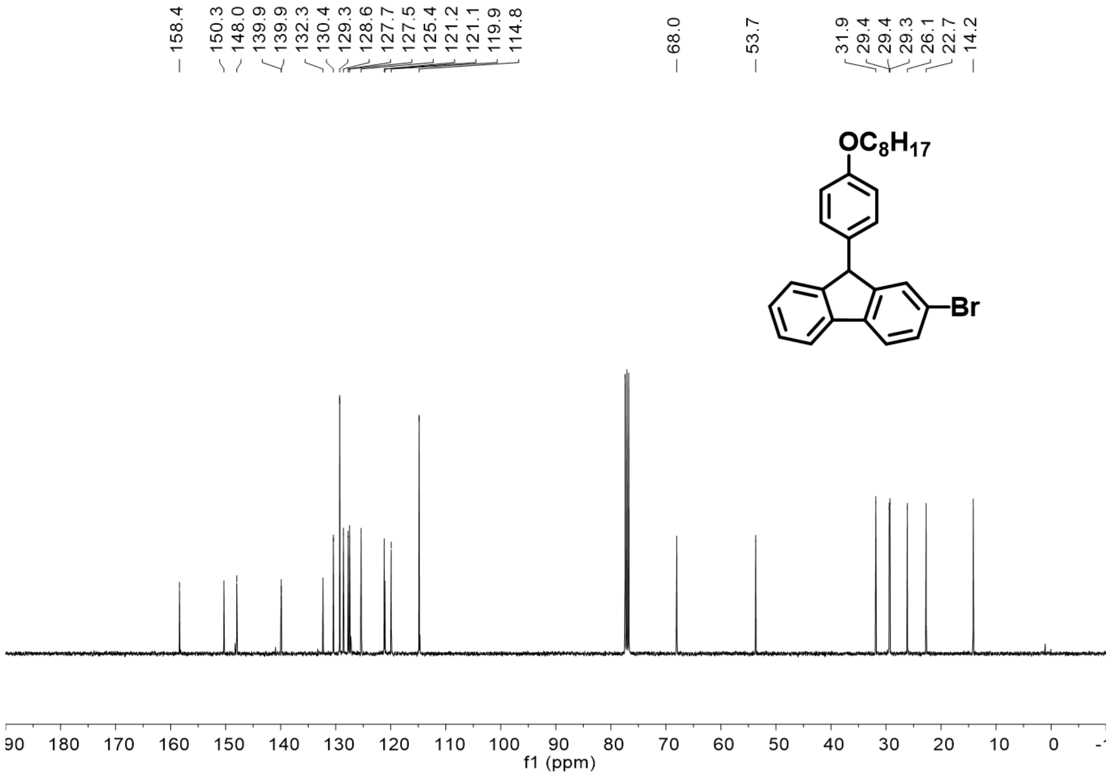

Supplementary Figure. 60 ｜ ^{1}H and ^{13}C-NMR Spectra of 1f. The bruker 400 MHz Fourier Transform NMR spectrometer was used to obtain ^{1}H and ^{13}C NMR spectra at a frequency of 400 MHz and 100 MHz in $CDCl_3$ at 20 °C, respectively.

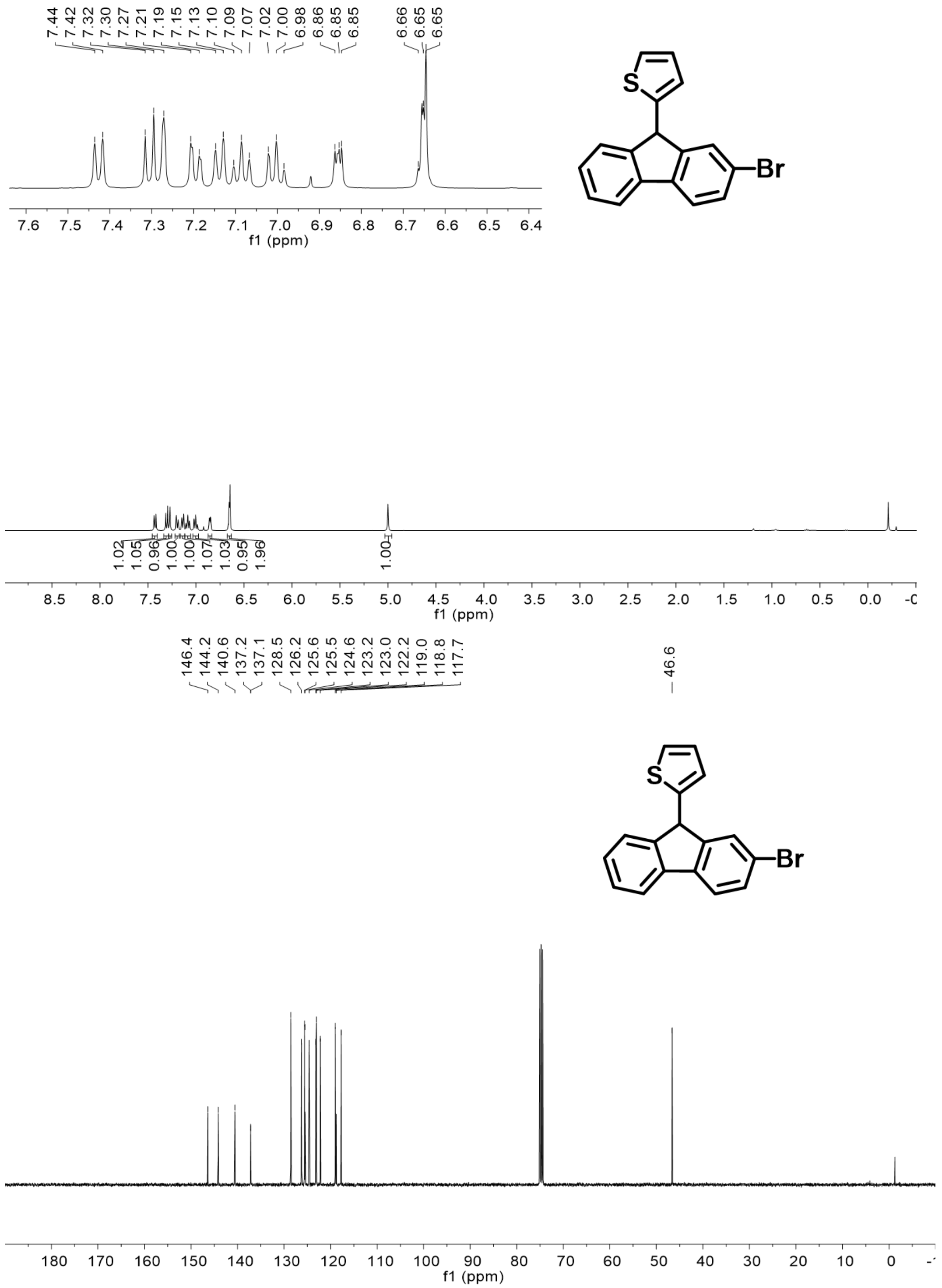

Supplementary Figure. 61 ｜ ^{1}H and ^{13}C-NMR Spectra of 1g. The bruker 400 MHz Fourier Transform NMR spectrometer was used to obtain ^{1}H and ^{13}C NMR spectra at a frequency of 400 MHz and 100 MHz in $CDCl_3$ at 20 °C, respectively.

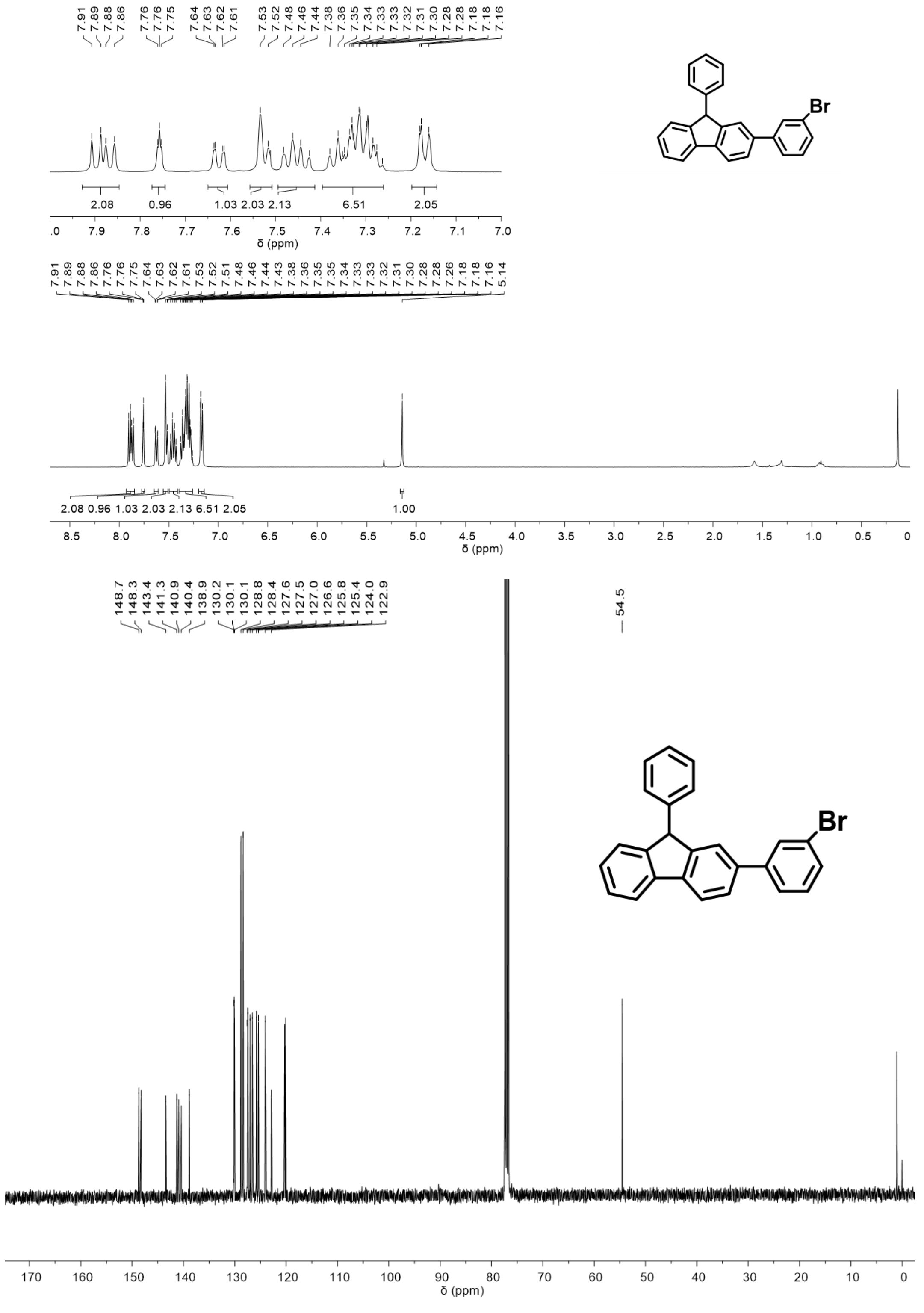

Supplementary Figure. 62 ｜ ^{1}H and ^{13}C-NMR Spectra of 1h. The bruker 400 MHz Fourier Transform NMR spectrometer was used to obtain ^{1}H and ^{13}C NMR spectra at a frequency of 400 MHz and 100 MHz in $CDCl_3$ at 20 ^{o}C, respectively.

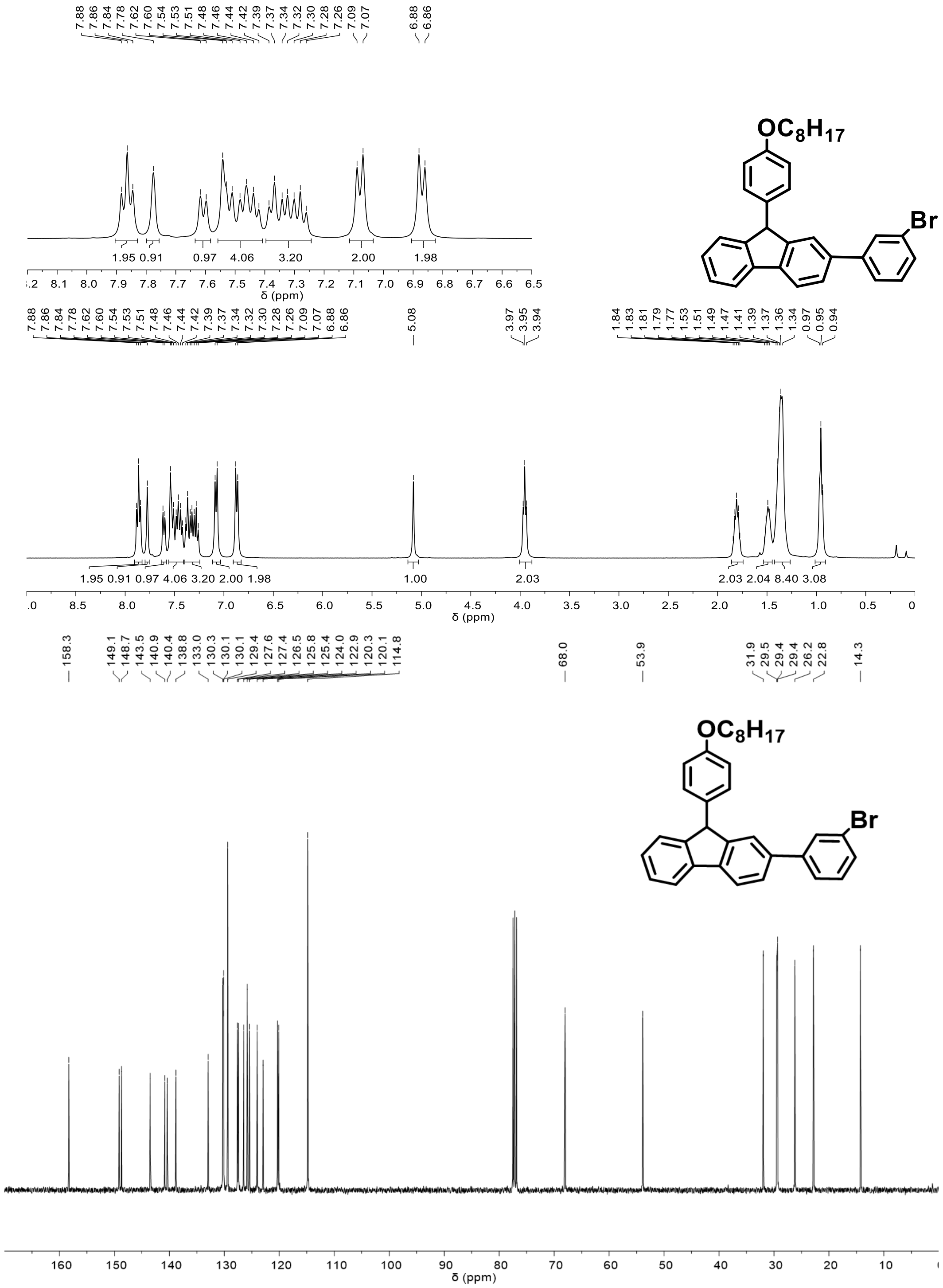

Supplementary Figure. 63 ｜ ^{1}H and ^{13}C-NMR Spectra of 1i. The bruker 400 MHz Fourier Transform NMR spectrometer was used to obtain ^{1}H and ^{13}C NMR spectra at a frequency of 400 MHz and 100 MHz in $CDCl_3$ at 20 ^{o}C, respectively.

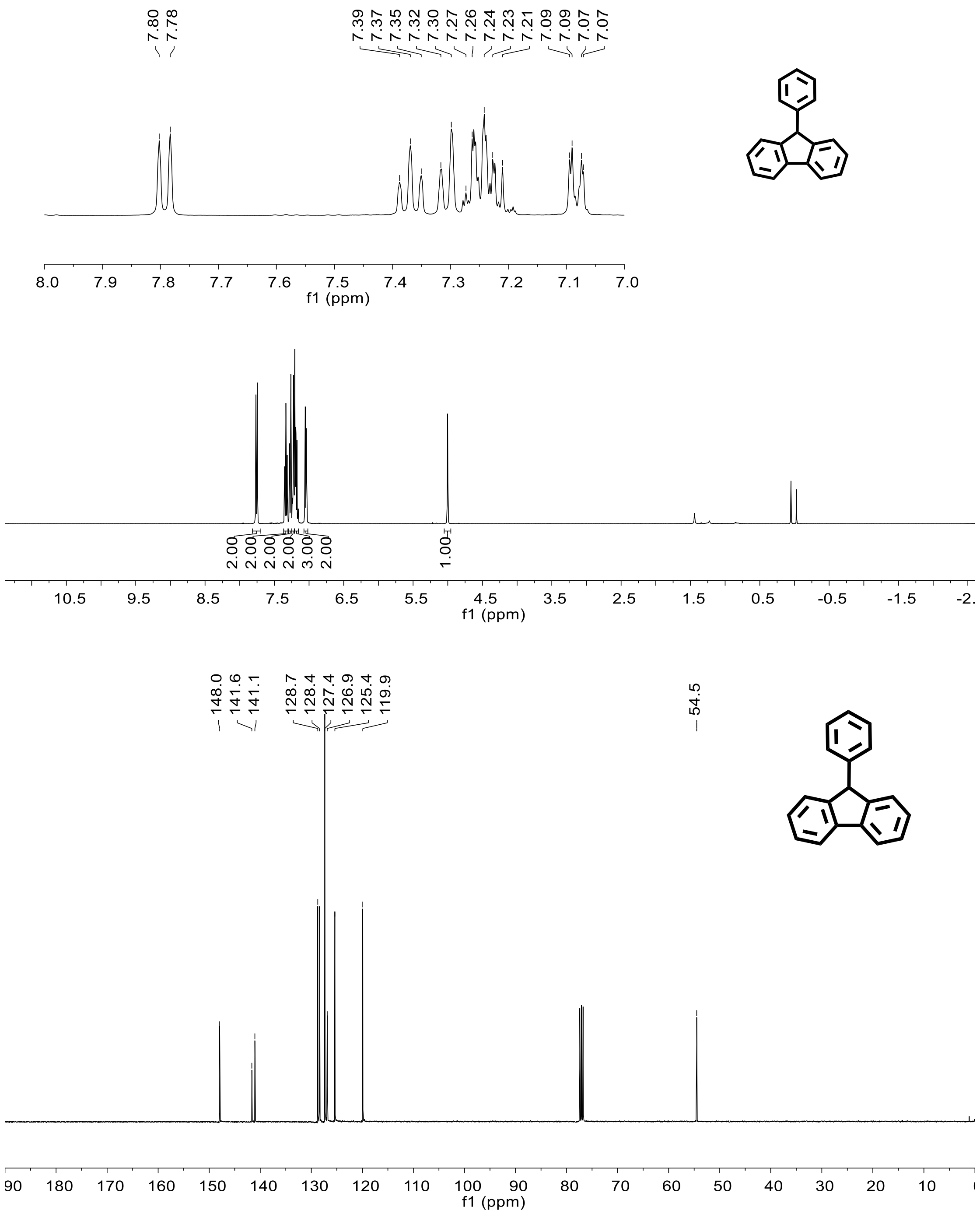

Supplementary Figure. 64 | ^{1}H and ^{13}C-NMR Spectra of 9 9-phenyl-*9H*-fluorene (2a). The bruker 400 MHz Fourier Transform NMR spectrometer was used to obtain ^{1}H and ^{13}C NMR spectra at a frequency of 400 MHz and 100 MHz in $CDCl_3$ at 20 °C, respectively.

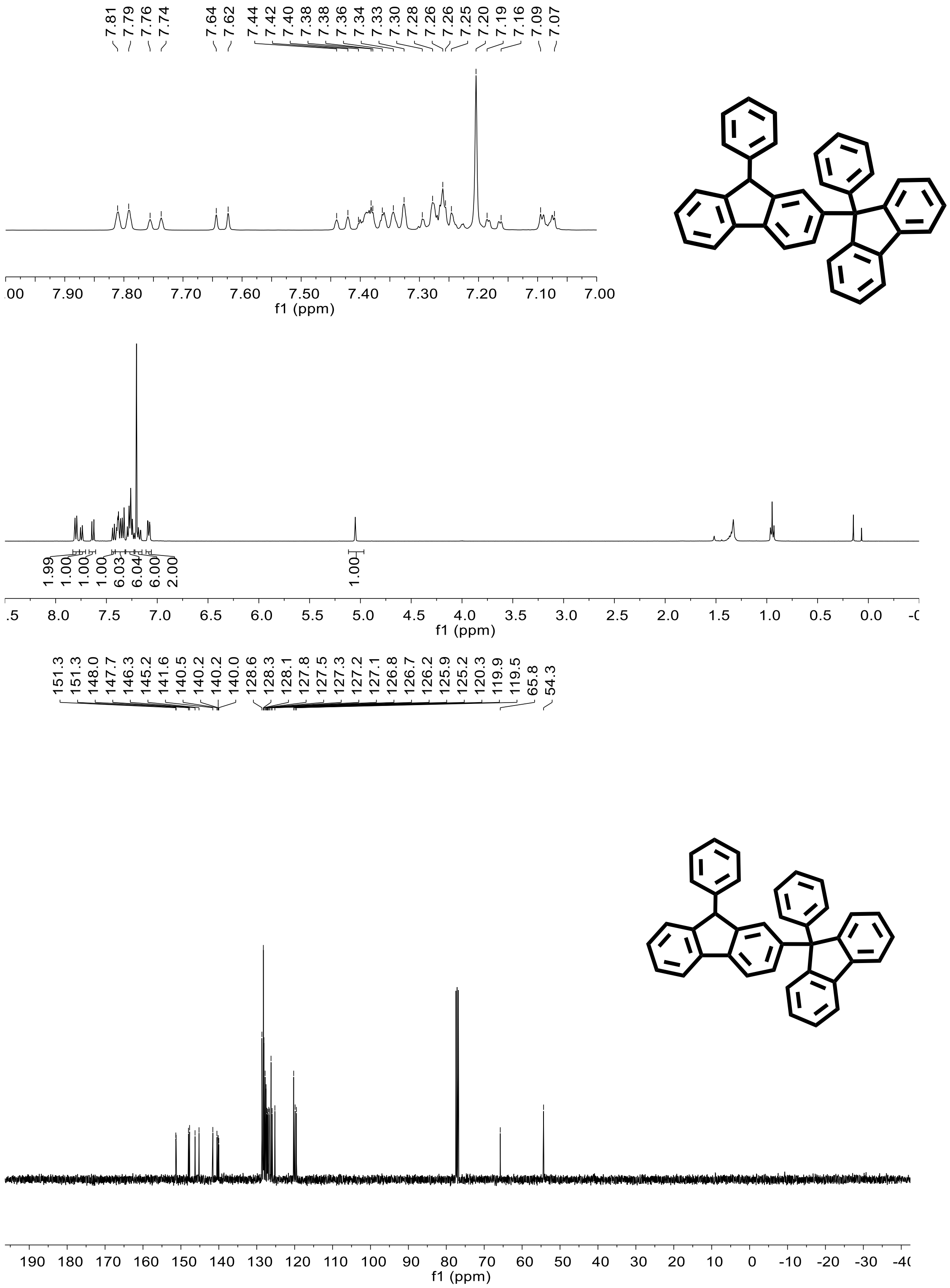

Supplementary Figurev. 65 ｜ ^{1}H and ^{13}C-NMR Spectra of 9-9'-diphenyl-9*H*,9'*H*-2,9'-bifluorene (3a).The bruker 400 MHz Fourier Transform NMR spectrometer was used to obtain ^{1}H and ^{13}C NMR spectra at a frequency of 400 MHz and 100 MHz in $CDCl_3$ at 20 °C, respectively.

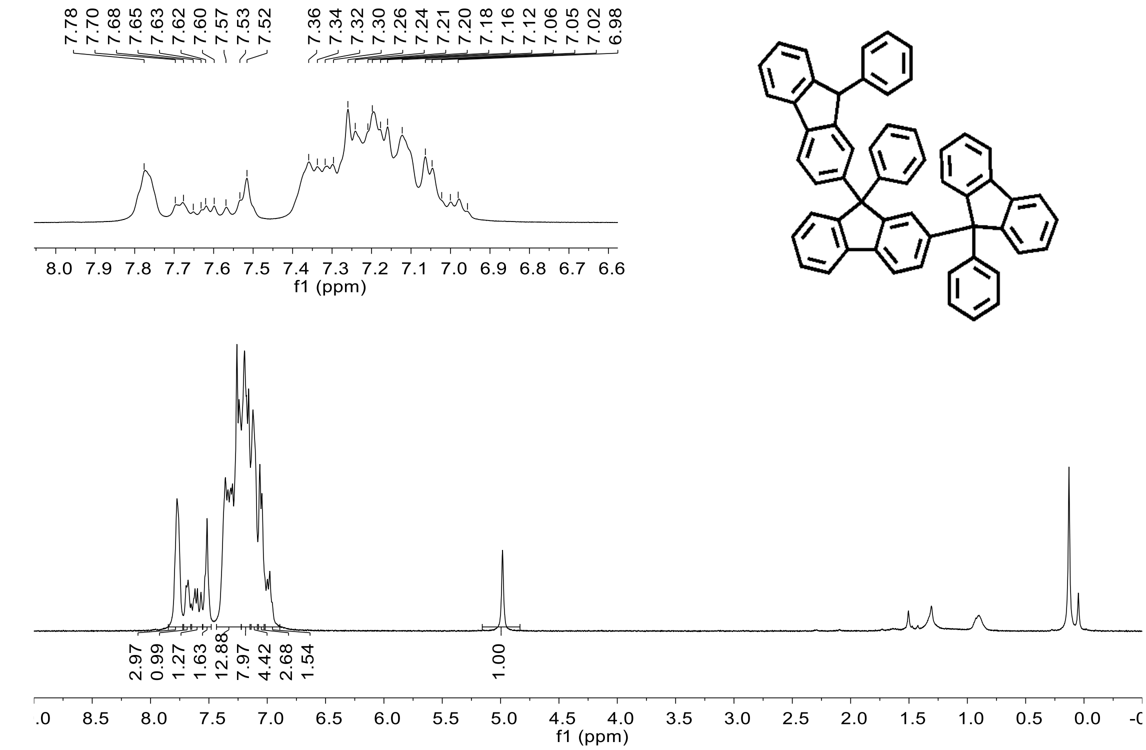

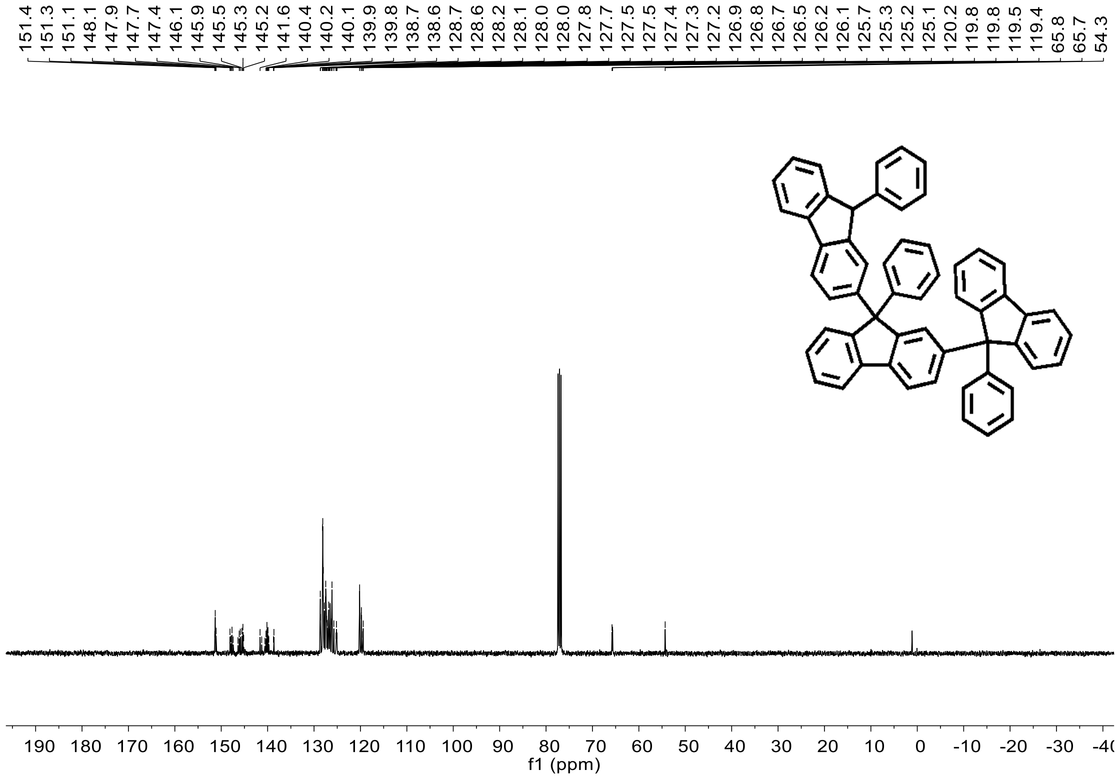

Supplementary Figure. 66 | ^{1}H and ^{13}C-NMR Spectra of 4a. The bruker 400 MHz Fourier Transform NMR spectrometer was used to obtain ^{1}H and ^{13}C NMR spectra at a frequency of 400 MHz and 100 MHz in $CDCl_3$ at 20 °C, respectively.

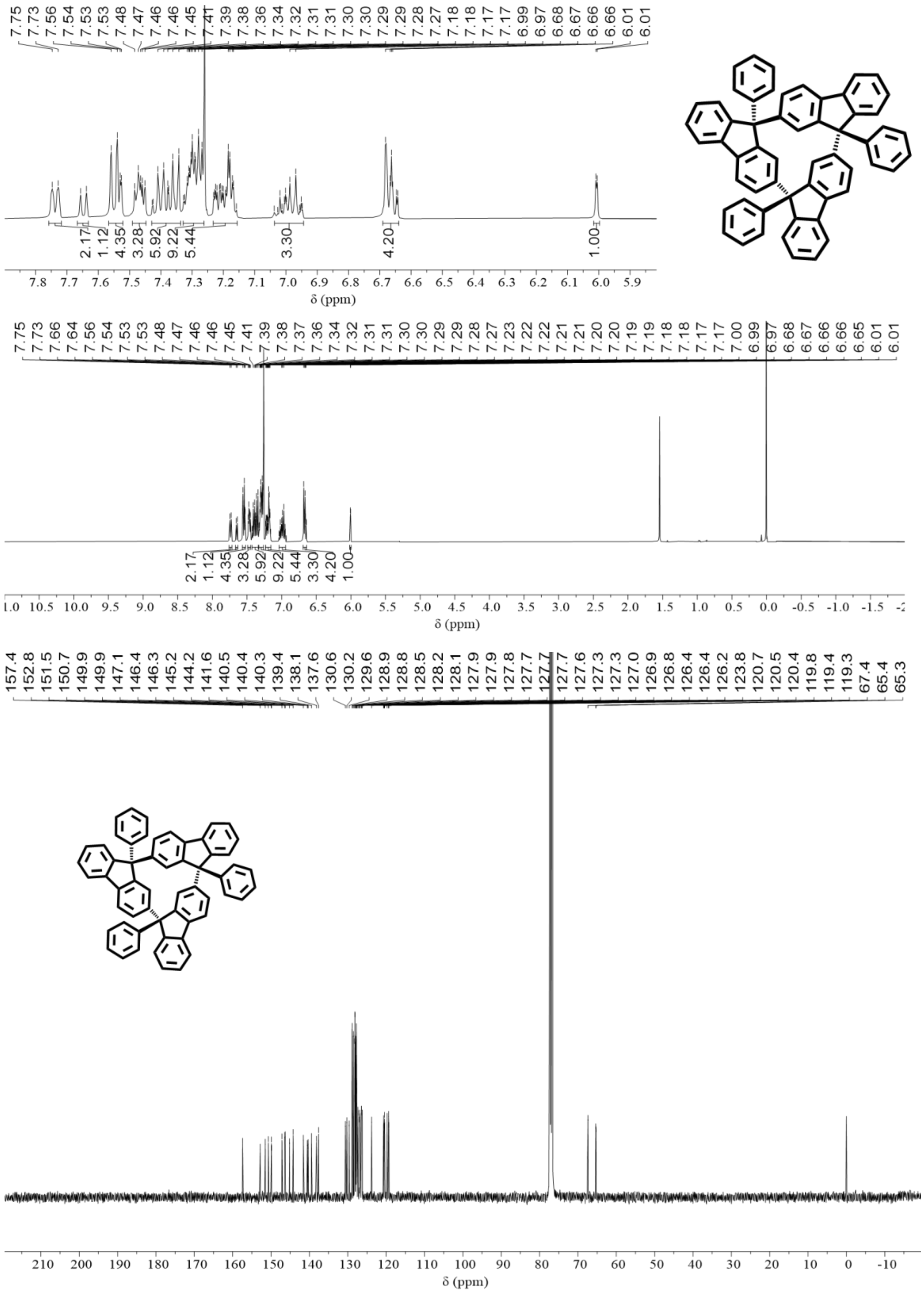

Supplementary Figure. 67 ｜^{1}H and ^{13}C-NMR Spectra of *cis-trans*-TWG1. The bruker 400 MHz Fourier Transform NMR spectrometer was used to obtain ^{1}H and ^{13}C NMR spectra at a frequency of 400 MHz and 100 MHz in $CDCl_3$ at 20 ^{o}C, respectively.

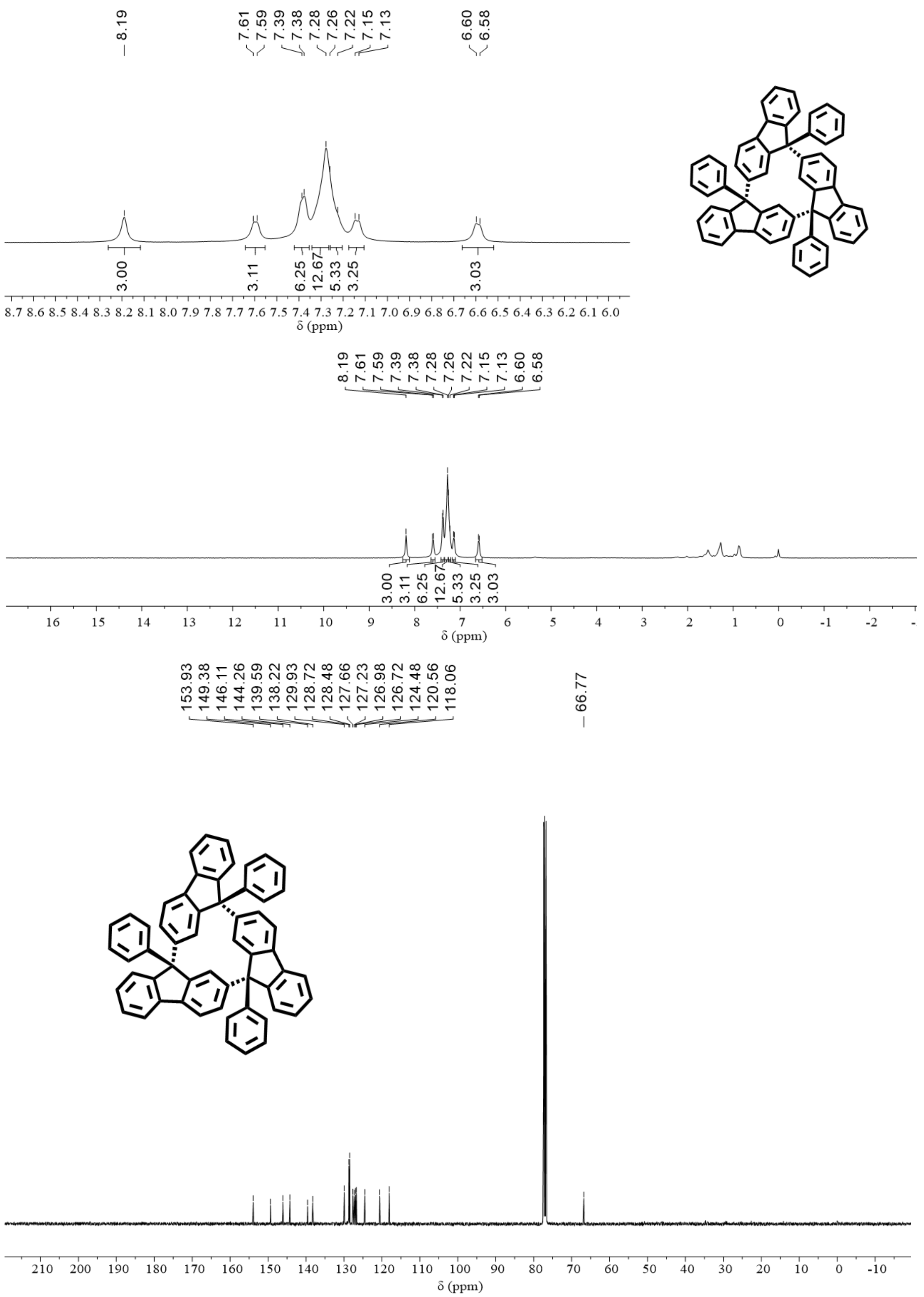

Supplementary Figure. 68 | ^{1}H and ^{13}C-NMR Spectra of *cis-cis*-TWG1. The bruker 400 MHz Fourier Transform NMR spectrometer was used to obtain ^{1}H and ^{13}C NMR spectra at a frequency of 400 MHz and 100 MHz in $CDCl_3$ at 20 °C, respectively.

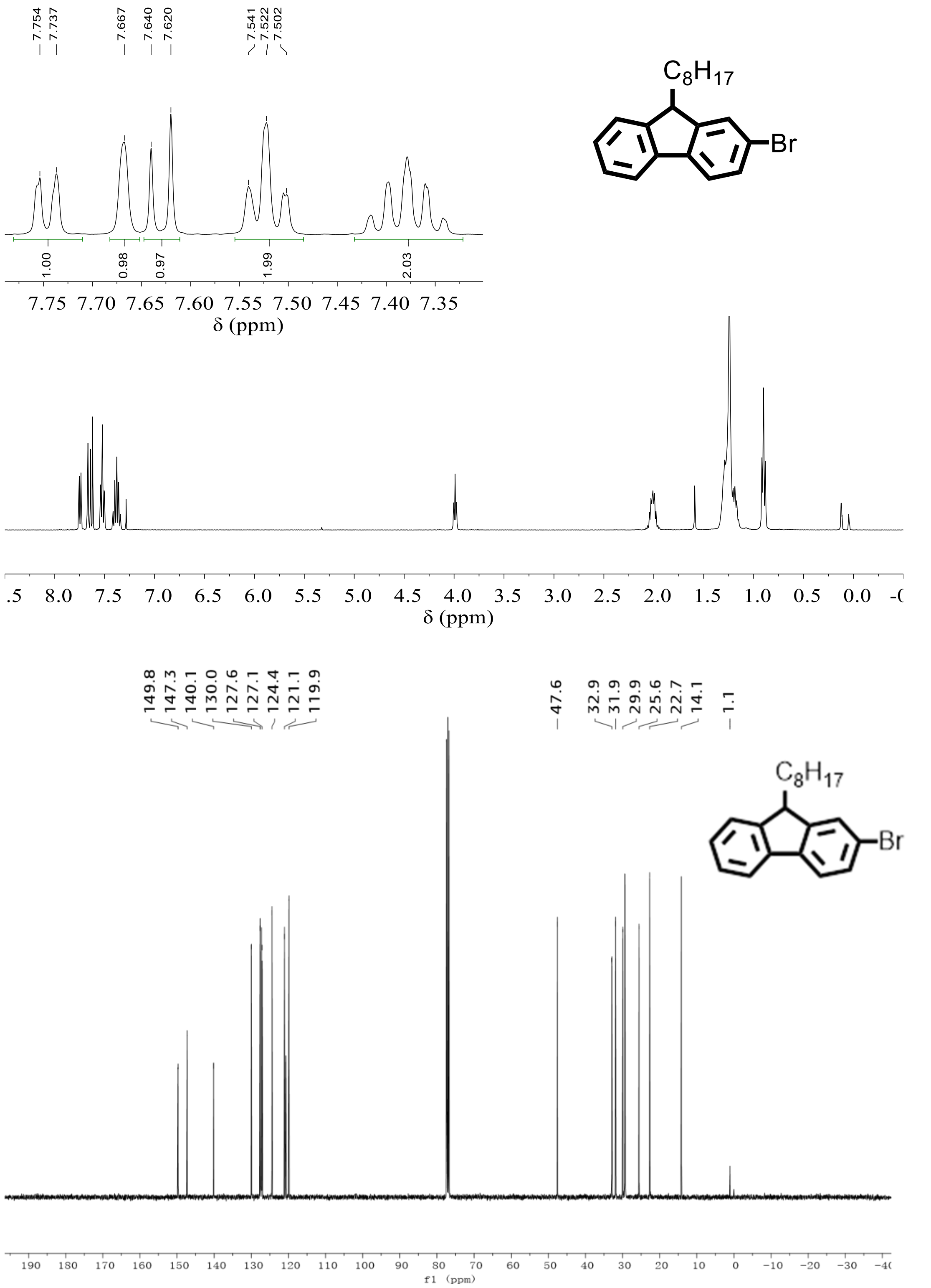

Supplementary Figure. 69 | ^{1}H and ^{13}C-NMR Spectra of 2-bromo-9-octyl-9H-fluorene. The bruker 400 MHz Fourier Transform NMR spectrometer was used to obtain ^{1}H and ^{13}C NMR spectra at a frequency of 400 MHz and 100 MHz in $CDCl_3$ at 20 °C, respectively.

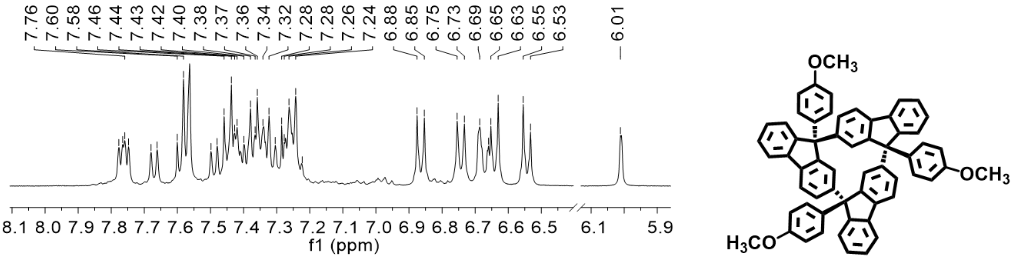

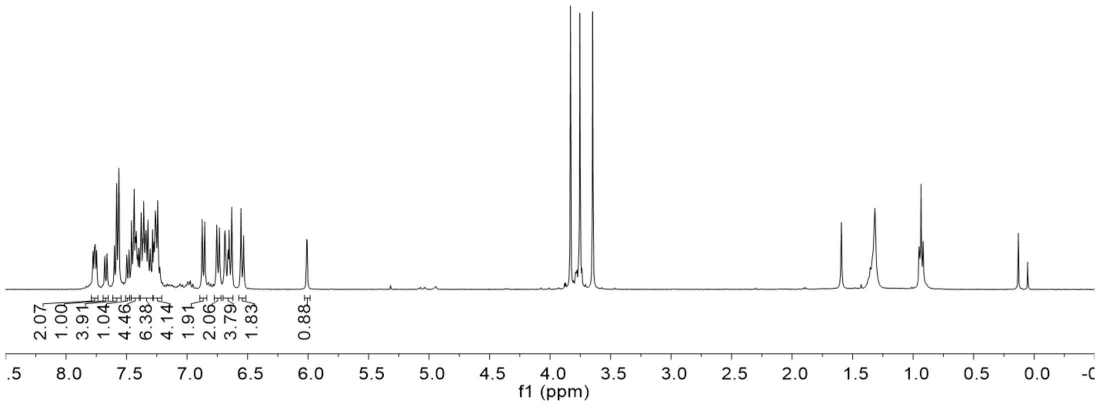

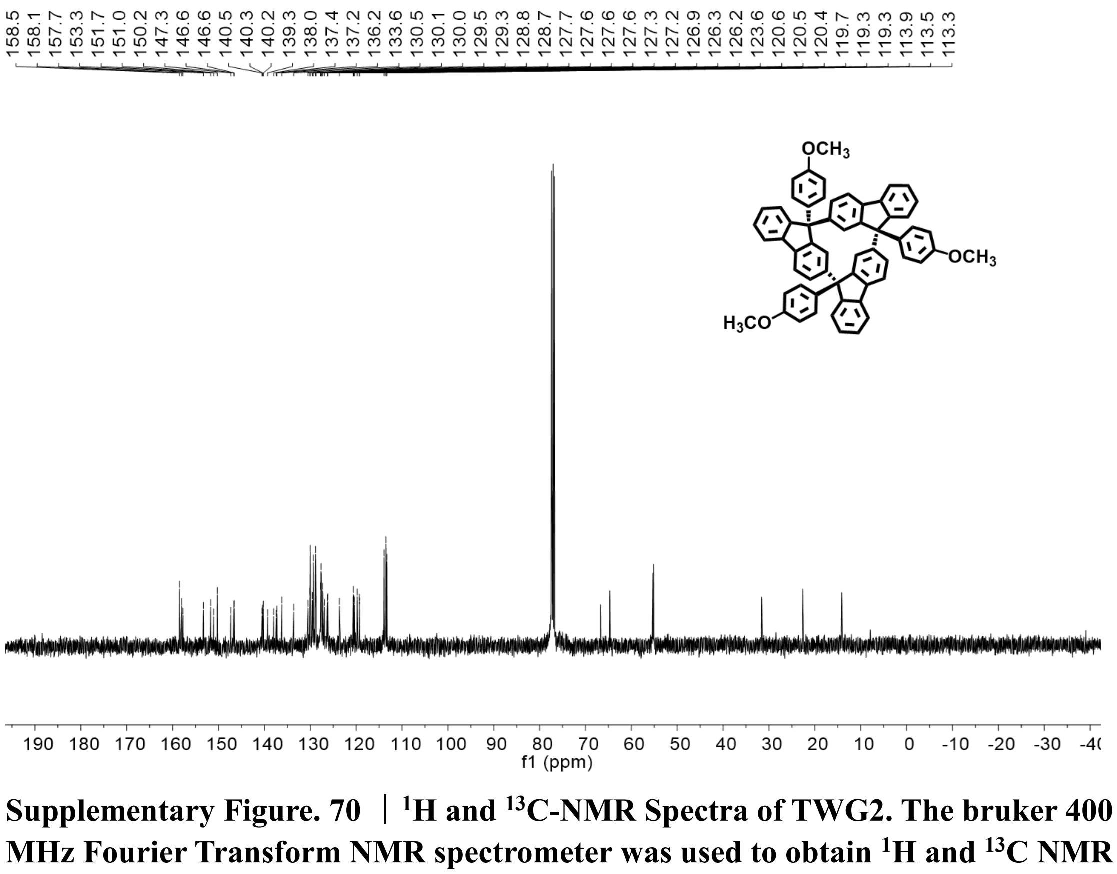

Supplementary Figure. 70 | ^{1}H and ^{13}C-NMR Spectra of TWG2. The bruker 400 MHz Fourier Transform NMR spectrometer was used to obtain ^{1}H and ^{13}C NMR spectra at a frequency of 400 MHz and 100 MHz in $CDCl_3$ at 20 ^{o}C, respectively.

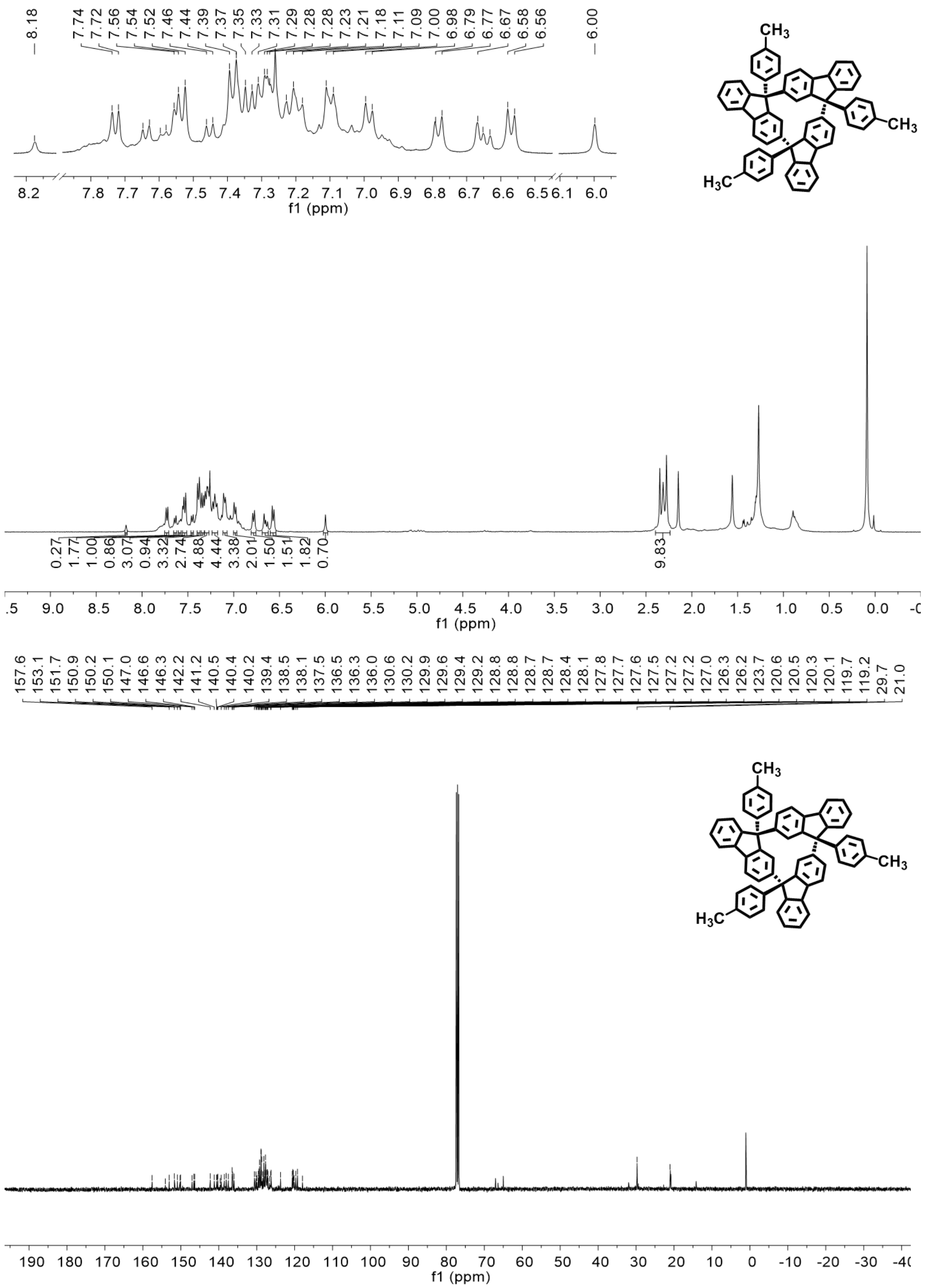

Supplementary Figure. 71 ｜ ^{1}H and ^{13}C-NMR Spectra of TWG3. The bruker 400 MHz Fourier Transform NMR spectrometer was used to obtain ^{1}H and ^{13}C NMR spectra at a frequency of 400 MHz and 100 MHz in $CDCl_3$ at 20 °C, respectively.

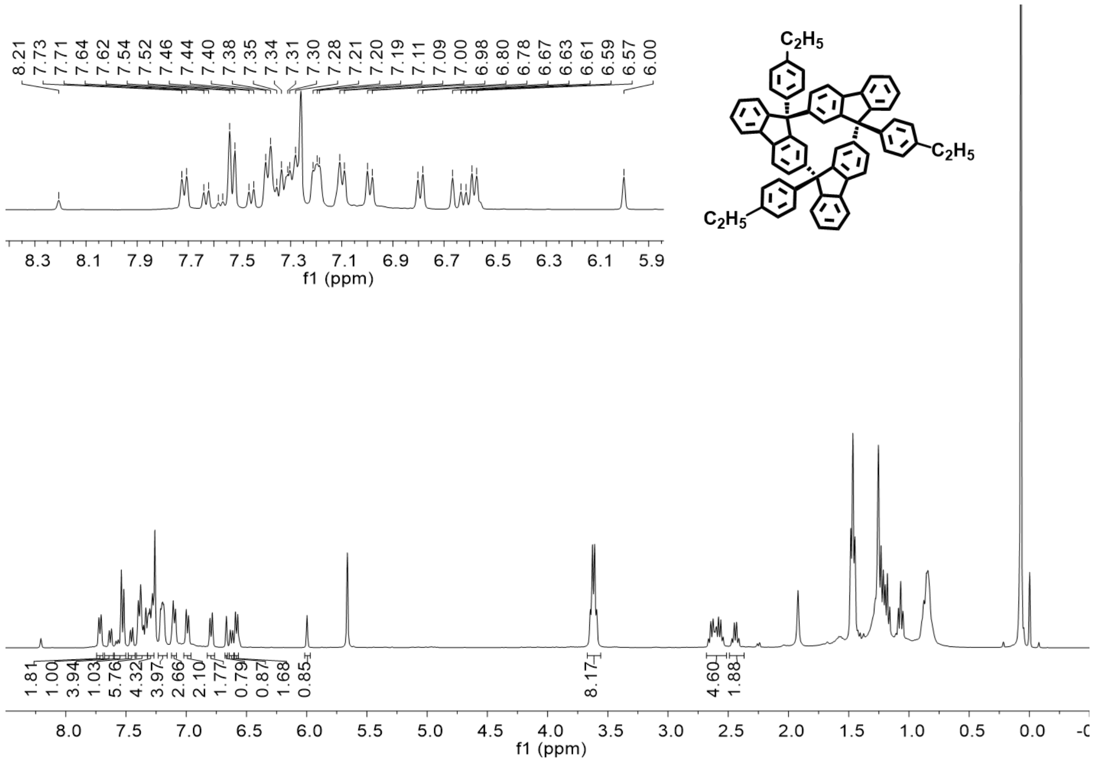

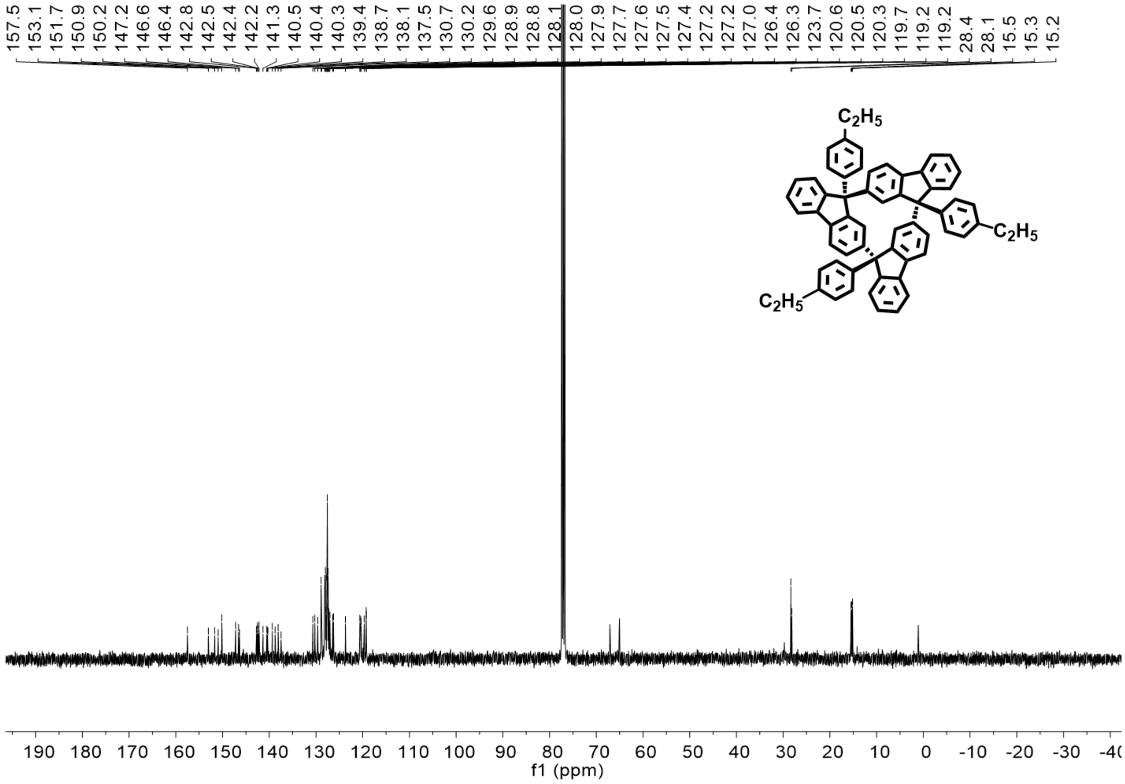

Supplementary Figure. 72 ｜ ^{1}H and ^{13}C-NMR Spectra of TWG4. The bruker 400 MHz Fourier Transform NMR spectrometer was used to obtain ^{1}H and ^{13}C NMR spectra at a frequency of 400 MHz and 100 MHz in $CDCl_3$ at 20 °C, respectively.

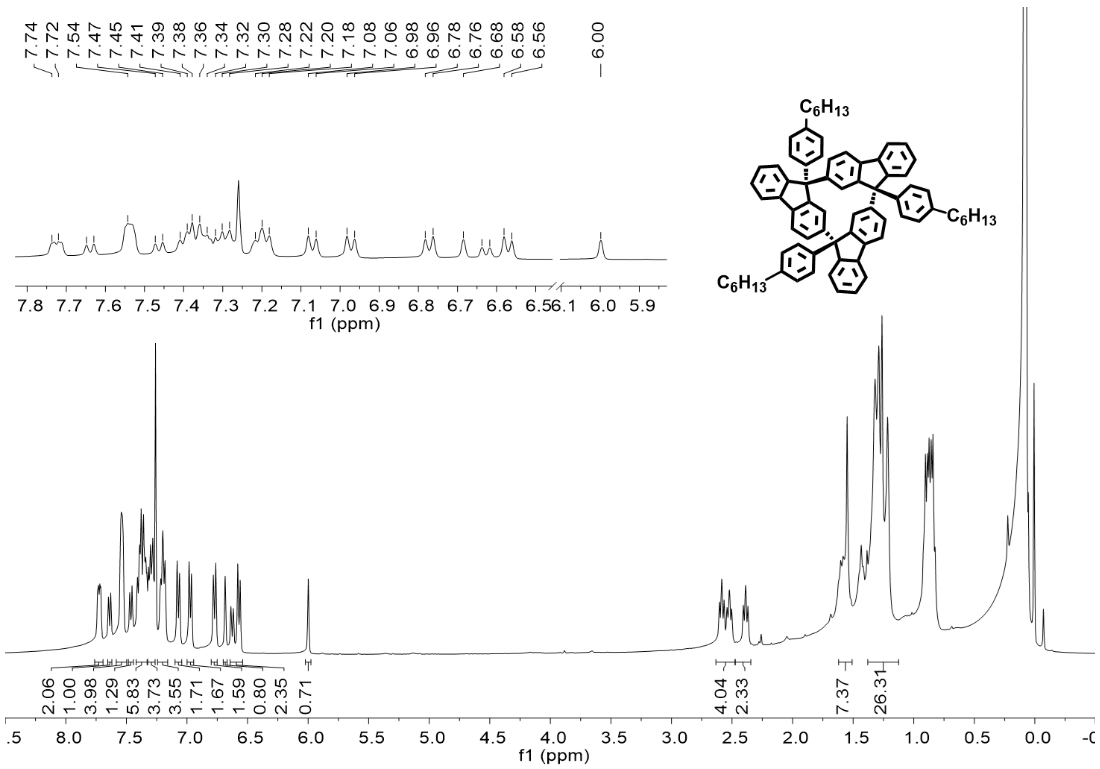

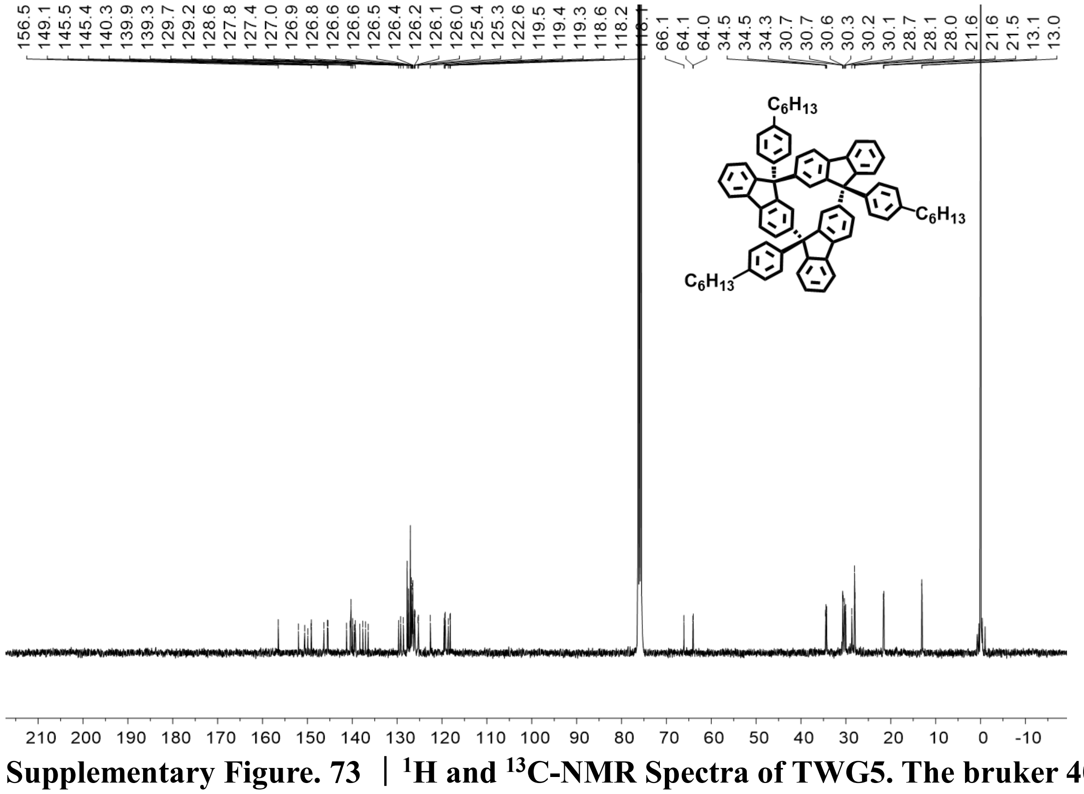

Supplementary Figure. 73 | ^{1}H and ^{13}C-NMR Spectra of TWG5. The bruker 400 MHz Fourier Transform NMR spectrometer was used to obtain ^{1}H and ^{13}C NMR spectra at a frequency of 400 MHz and 100 MHz in $CDCl_3$ at 20 °C, respectively.

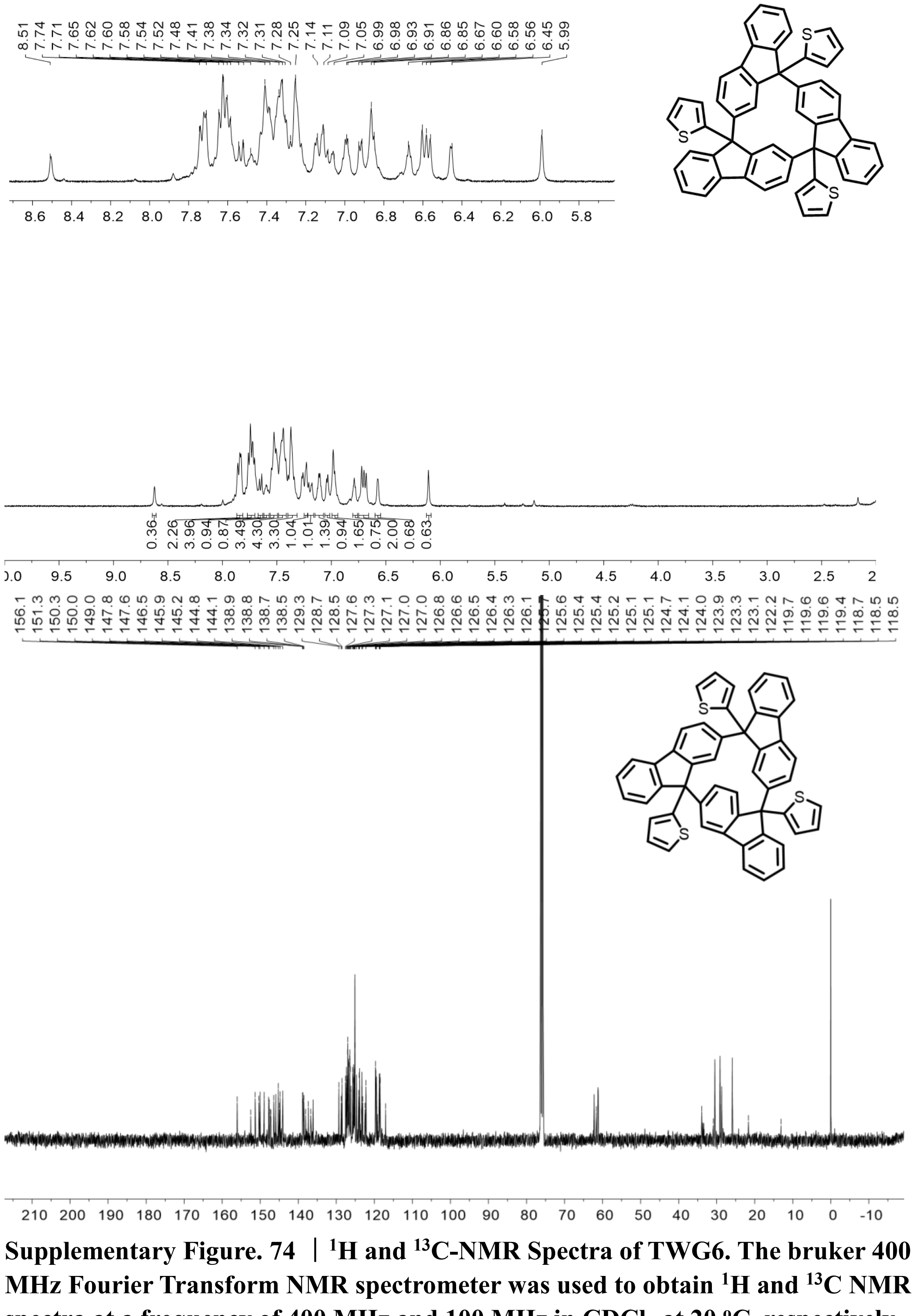

Supplementary Figure. 74 | ^{1}H and ^{13}C-NMR Spectra of TWG6. The bruker 400 MHz Fourier Transform NMR spectrometer was used to obtain ^{1}H and ^{13}C NMR spectra at a frequency of 400 MHz and 100 MHz in $CDCl_3$ at 20 ^{o}C, respectively.

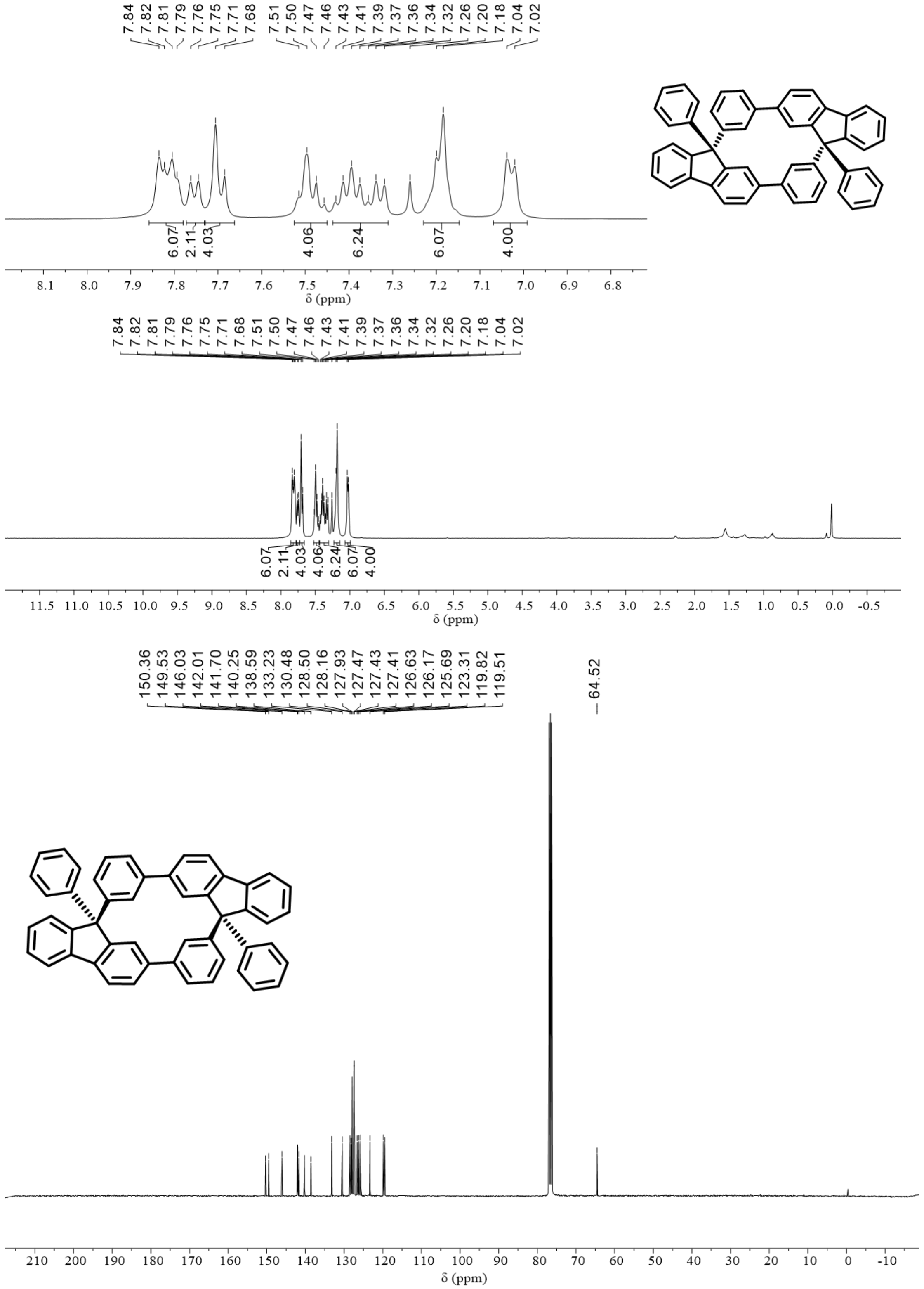

Supplementary Figure. 75 ｜ ^{1}H and ^{13}C-NMR Spectra of *rac*-DWG1. The bruker 400 MHz Fourier Transform NMR spectrometer was used to obtain ^{1}H and ^{13}C NMR spectra at a frequency of 400 MHz and 100 MHz in $CDCl_3$ at 20 ^{o}C, respectively.

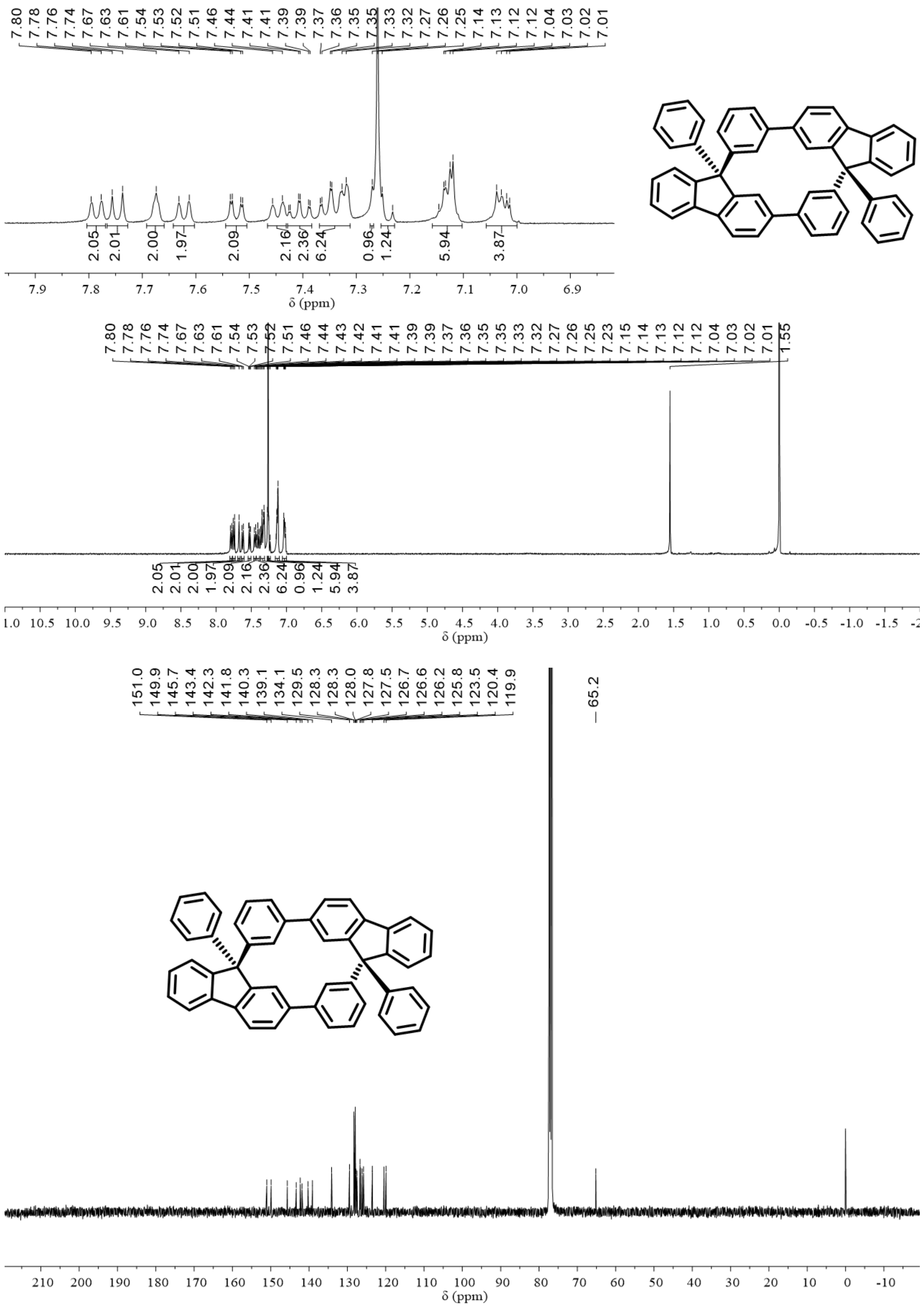

Supplementary Figure. 76 | ^{1}H and ^{13}C-NMR Spectra of *meso*-DWG1. The bruker 400 MHz Fourier Transform NMR spectrometer was used to obtain ^{1}H and ^{13}C NMR spectra at a frequency of 400 MHz and 100 MHz in $CDCl_3$ at 20 °C, respectively.

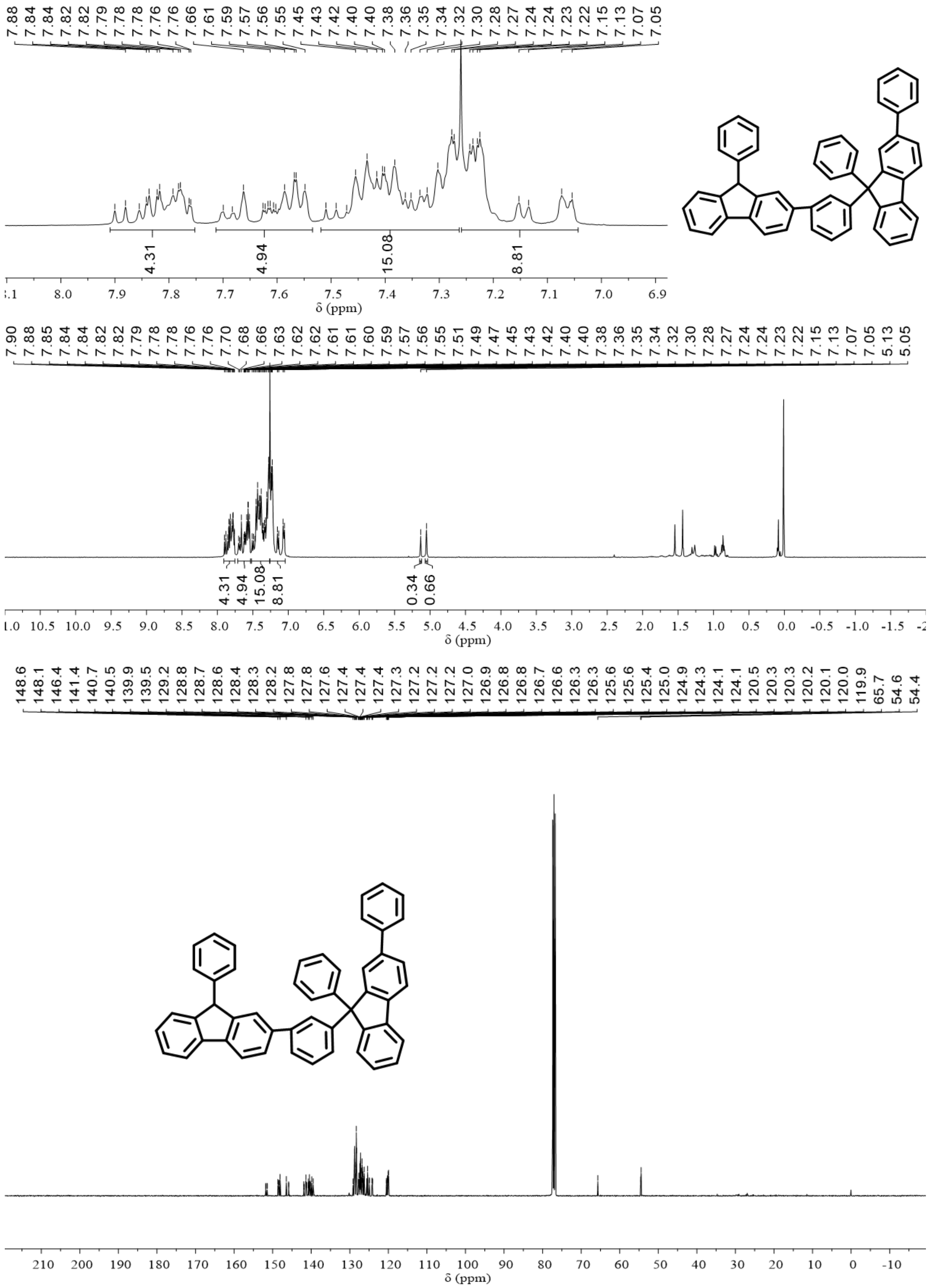

Supplementary Figure. 77 | ^{1}H and ^{13}C-NMR Spectra of 3h. The bruker 400 MHz Fourier Transform NMR spectrometer was used to obtain ^{1}H and ^{13}C NMR spectra at a frequency of 400 MHz and 100 MHz in $CDCl_3$ at 20 °C, respectively.

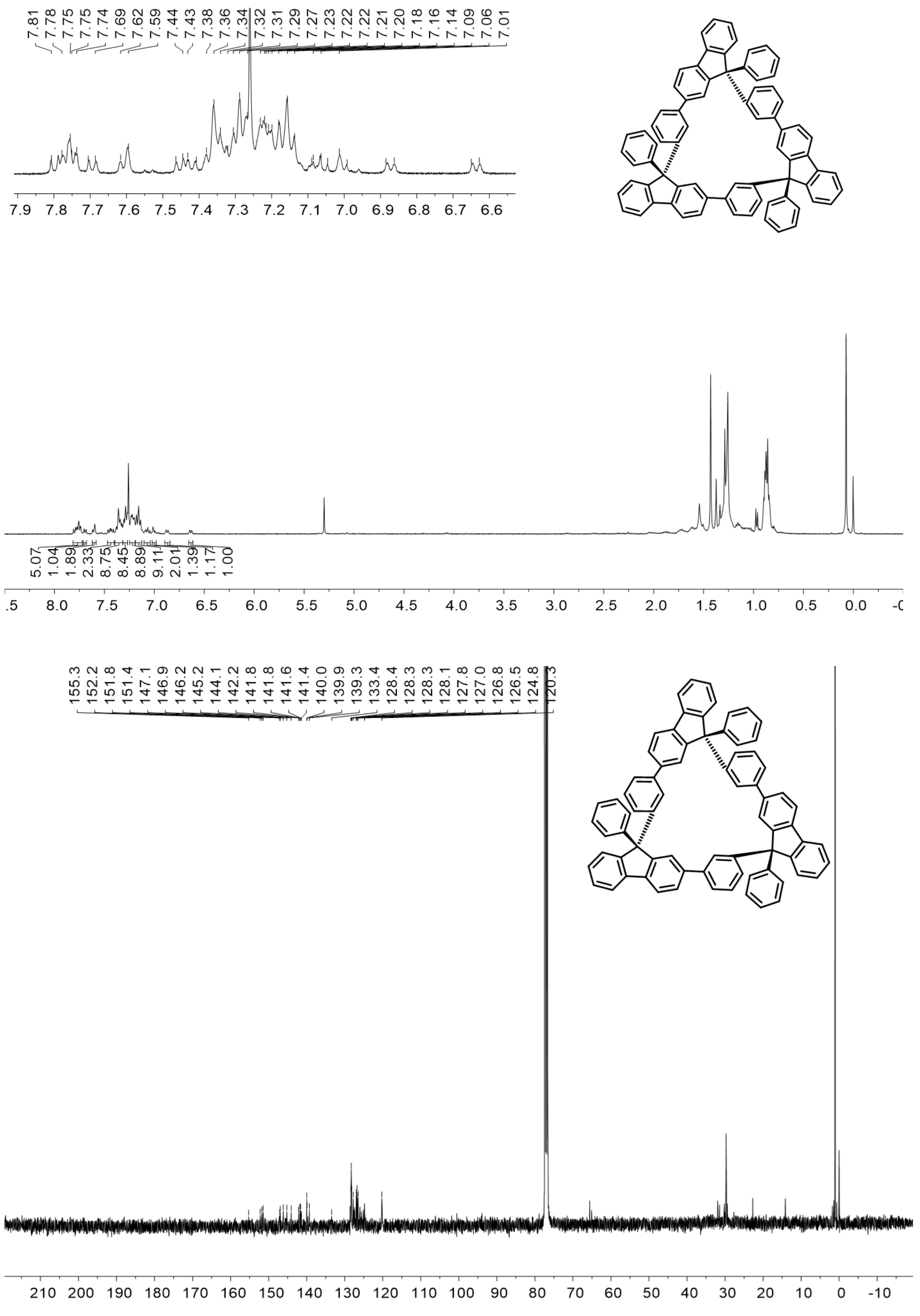

Supplementary Figure. 78 | ^{1}H and ^{13}C-NMR Spectra of *cis-cis*-TWG8. The bruker 400 MHz Fourier Transform NMR spectrometer was used to obtain ^{1}H and ^{13}C NMR spectra at a frequency of 400 MHz and 100 MHz in $CDCl_3$ at 20 ^{o}C, respectively.

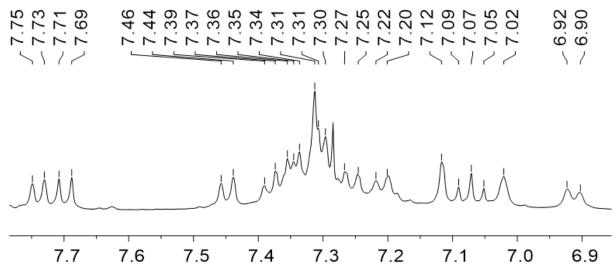

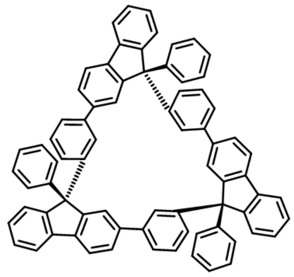

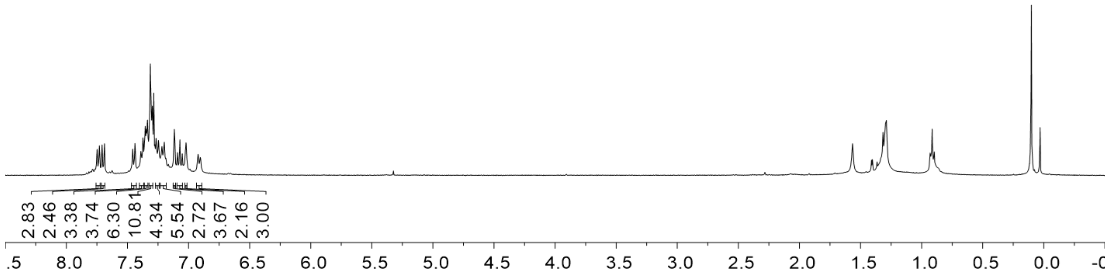

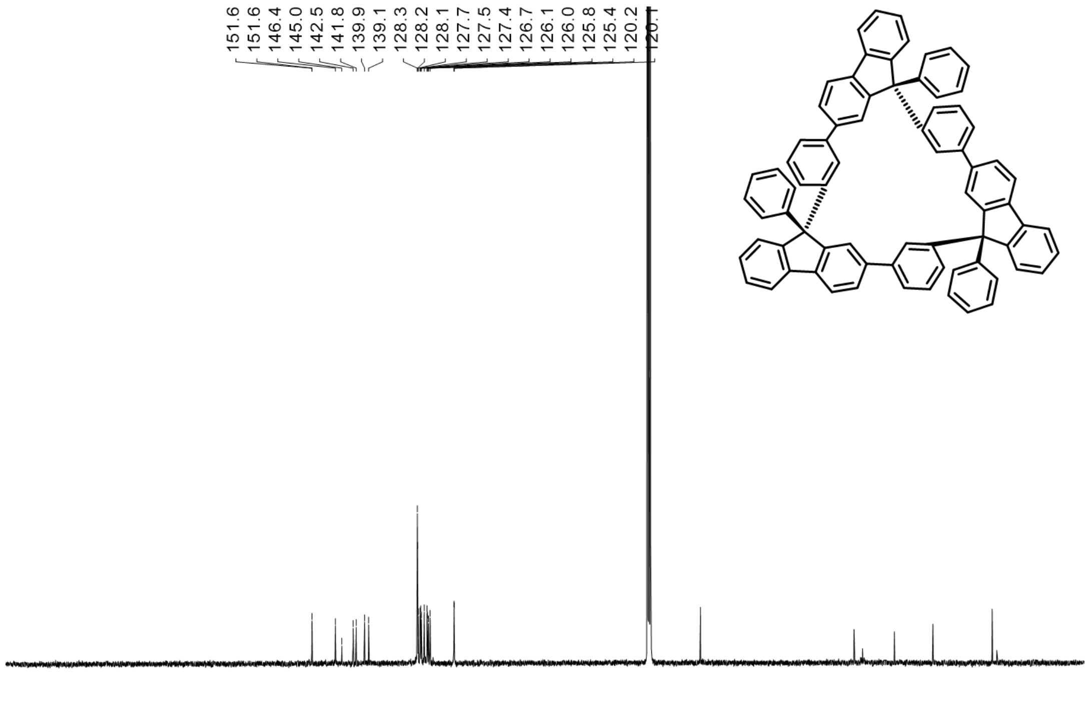

Supplementary Figure. 79 | ^{1}H and ^{13}C-NMR Spectra of *cis-trans*-TWG8. The bruker 400 MHz Fourier Transform NMR spectrometer was used to obtain ^{1}H and ^{13}C NMR spectra at a frequency of 400 MHz and 100 MHz in $CDCl_3$ at 20 ^{o}C, respectively.

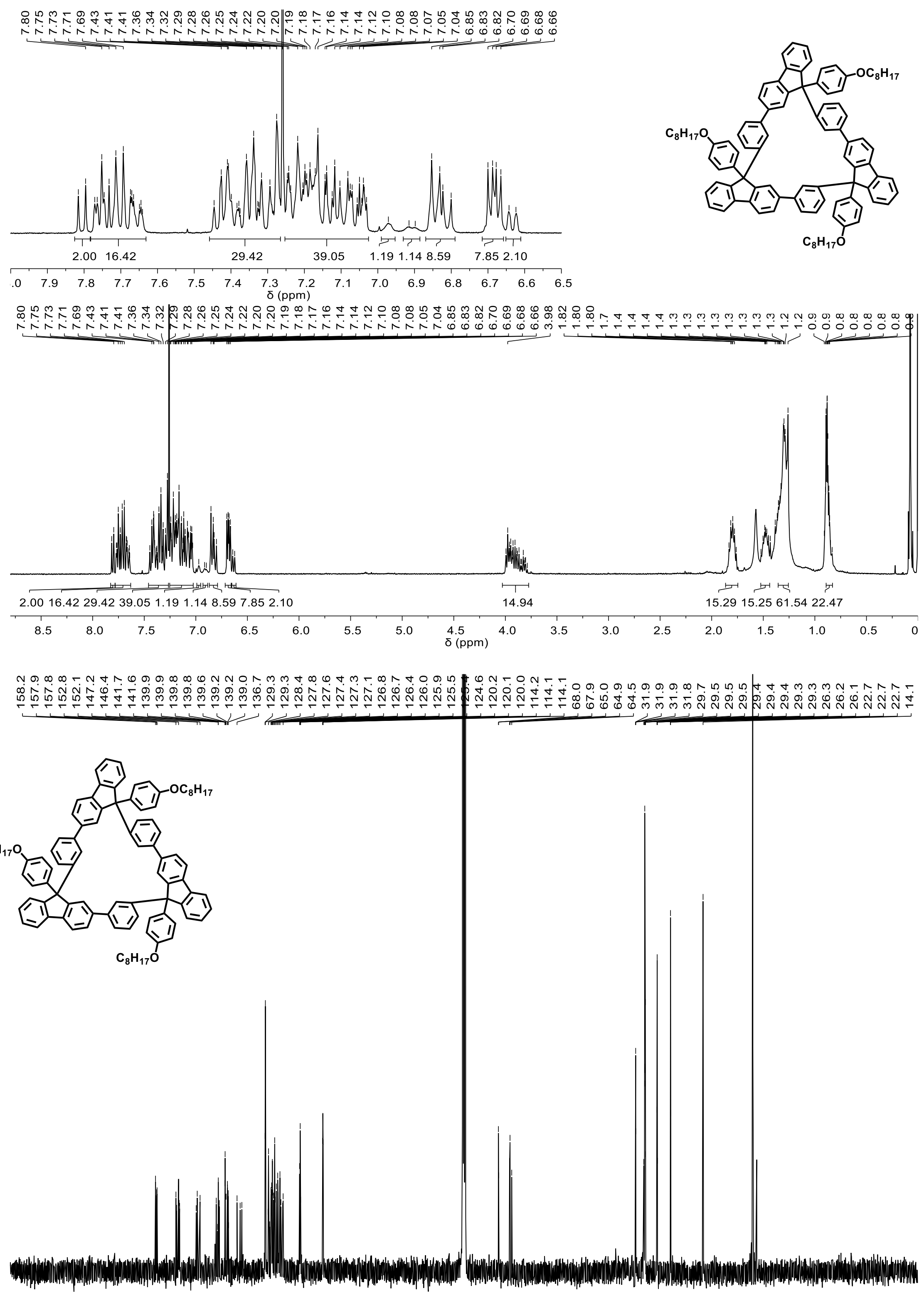

Supplementary Figure. 80 | ^{1}H and ^{13}C-NMR Spectra of TWGs9. The bruker 400 MHz Fourier Transform NMR spectrometer was used to obtain ^{1}H and ^{13}C NMR spectra at a frequency of 400 MHz and 100 MHz in $CDCl_3$ at 20 °C, respectively.

Supplementary References

1. Chen, S & Xu, H. Electroluminescent materials toward near ultraviolet region. *Chem. Soc. Rev.* **50**, 8639-8668 (2021).
2. Reddy, M. D. et al. Palladium-Catalyzed Direct Arylation of C(sp^3)-H Bonds of α-Cyano Aliphatic Amides. *J. Org. Chem.* **80**, 11447-11459 (2015).
3. Chao, T. C. et al. Efficient UV organic light-emitting devices based on bi(9,9-diarylfluorene)s. *Adv. Mater.* **34,** 992-996 (2005).
4. Cheeseman, J. R.; Scalmani, G.; Barone, V.; Mennucci, B.; Petersson, G. A.; Nakatsuji, H.; Caricato, M.; Li, X.; Hratchian, H. P.; Izmaylov, A. F.; Bloino, J.; Zheng, G.; Sonnenberg, J. L.; Hada, M.; Ehara, M.; Toyota, K.; Fukuda, R.; Hasegawa, J.; Ishida, M.; Nakajima, T.; Honda, Y.; Kitao, O.; Nakai, H.; Vreven, T.; Montgomery, J. A., Jr.; Peralta, J. E.; Ogliaro, F.; Bearpark, M.; Heyd, J. J.; Brothers, E.; Kudin, K. N.; Staroverov, V. N.; Kobayashi, R.; Normand, J.; Raghavachari, K.; Rendell, A.; Burant, J. C.; Iyengar, S. S.; Tomasi, J.; Cossi, M.; Rega, N.; Millam, J. M.; Klene, M.; Knox, J. E.; Cross, J. B.; Bakken, V.; Adamo, C.; Jaramillo, J.; Gomperts, R.; Stratmann, R. E.; Yazyev, O.; Austin, A. J.; Cammi, R.; Pomelli, C.; Ochterski, J. W.; Martin, R. L.; Morokuma, K.; Zakrzewski, V. G.; Voth, G. A.; Salvador, P.; Dannenberg, J. J.; Dapprich, S.; Daniels, A. D.; Farkas, O.; Foresman, J. B.; Ortiz, J. V.; Cioslowski, J.; Fox, D. J. Gaussian 09, C.1, Gaussian, Inc., Wallingford, CT, 2009.
5. Zou, S. N. et al. Efficient violet organic light-emitting diodes with CIEy of 0.02 based on spiro skeleton. *Advanced Optl Materials.* **8,** 2001074 (2020).
6. Tasch, S. et al Red-orange electroluminescence with new soluble and air-stable poly(naphthalene-vinylene). *Adv. Mater.***7,** 903-906 (1995).
7. Song, C. Y. et al. Sb_2O_3/Ag/Sb_2O_3 multilayer transparent conducting films for ultraviolet organic light-emitting diode. *Sci. Rep*. **7,** 41250 (2017).
8. Yuan, Y. F. et al. Tailoring hole injection of sol–gel processed WO_x and its doping in PEDOT:PSS for efficient ultraviolet organic light-emitting diodes. *Phys. Chem. Chem. Phys.* **22,** 13214-13222 (2020).
9. Zheng, Q. H. et al. Solution-processed aqueous composite hole injection layer of PEDOT:PSS+MoO_x for efficient ultraviolet organic light-emitting diode.*Org. Electron.* **46,** 7-13 (2017).
10. Zhang, X. W. et al. Facile synthesis of solution-processed MoS_2 nanosheets and their application in high-performance ultraviolet organic light-emitting diodes. *J. Mater. Chem. C.* **7,** 926-936 (2019).
11. Zhang, X. W. et al. Exceeding 4% external quantum efficiency in ultraviolet organic light-emitting diode using PEDOT:PSS/MoO_x double-stacked hole injection layer. *Appl. Phys. Lett.* **110,** 043301 (2017).
12. Na, J. et al. Achieving the hypsochromic electroluminescence of ultraviolet OLED by tuning excitons relaxation. *Org. Electron.* **82,** 105718 (2020).
13. Zhang, Q. et al. Highly efficient ultraviolet organic light-emitting diodes and interface study using impedance spectroscopy. *Optik.* **126,** 1595-1597 (2015).
14. Yang, Y. X. et al. Ultraviolet-violet electroluminescence from highly fluorescent purines. *J. Mater. Chem. C.* **1,** 2867-2874 (2013).
15. Sharma, A. et al. Room temperature ultraviolet emission at 357 nm from polysilane based organic light emitting diode. Appl. *Phys. Lett.* **88,** 143511 (2006).

16. Etori, H. et al. Spirobifluorene derivatives for ultraviolet organic light-emitting diodes. *Synth. Met.* **156,** 1090-1096 (2006).
17. Zou, L. et al. Combinatorial fabrication and studies of intense efficient ultraviolet--violet organic light-emitting device arrays. *Appl. Phys. Lett.* **79,** 2282–2284 (2001).
18. Wang, Z. Q. et al. Efficient violet non-doped organic light-emitting device based 4 on a pyrene derivative with novel molecular structure. *Org. Electron.* **23,** 179-185 (2015).
19. Wong, K. T. et al. Spiro-configured bifluorenes: highly efficient emitter for UV organic light-emitting device and host material for red electrophosphorescence. *Org. Lett.* **7,** 5131-5134 (2005).
20. Joseph, V. et al. Asymmetrically 2,7-difunctionalized carbazole-based donor-acceptor hybrids for deep blue electroluminescence applications. *Opt. Mater*. **108,** 110159 (2020).
21. Lin, J. et al. Highly efficient microcavity organic light-emitting devices with narrow-band pure UV emission. *ACS Appl. Mater. Interfaces*. **12,** 10717-10726 (2020).
22. Li, G. et al. Efficient ultraviolet organic light-emitting diodes with a CIEy of 0.04 and negligible-efficiency roll-off. *ACS Appl. Mater. Interfaces*. **14,** 10627–10636 (2022).
23. Zhong, Z. T. et al. High steric-hindrance windmill-type molecules for efficient ultraviolet to pure-blue organic light-emitting diodes via hybridized local and charge-transfer excited-state. *Adv. Funct. Mater.* **32,** 2112969 (2022).
24. Zhong, Z. T. et al. Highly twisted bipolar molecules for efficient near-ultraviolet organic light-emitting diodes via a hybridized local and charge-transfer mechanism. *J. Mater. Chem. C.* **11**, 1733-1741 (2023).
25. Hou, Y. M. et al. Structurally regulated carbazole–pyridine derivatives based on space-crowded theory for efficient narrowband ultraviolet nondoped organic light-emitting diodes from the high-lying reverse intersystem crossing process. *ACS Appl. Mater. Interfaces.* **14,** 57092-57101 (2022).
26. Zhang, H. et al. High-performance ultraviolet organic light-emitting diode enabled by high-lying reverse intersystem crossing. *Angew. Chem. Int. Ed.* **60,** 22241-22247 (2021).
27. Chen, J. K. et al. Robust luminescent molecules with high-level reverse intersystem crossing for efficient near ultraviolet organic light-emitting diodes. *Angew. Chem. Int. Ed.* **61,** 202116810 (2022).
28. Luo, Y. J. et al. An ultraviolet thermally activated delayed fluorescence OLED with total external quantum efficiency over 9%. *Adv. Mater.* **32,** 2001248 (2020).
29. Ma, P. et al. High-efficiency ultraviolet electroluminescence from multi-resonance phosphine oxide polycyclic aromatics. *Angew. Chem. Int. Ed.* 202316479 (2023).
